# How Phylogenies Inferred from 46 Mitochondrial Genomes Informed the Systematics of the Australasian Genus of Ticks, *Bothriocroton*, with the Description of Three New Species

**DOI:** 10.3390/biology15191733

**Published:** 2026-10-01

**Authors:** Samuel Kelava, Mingeun Cho, Finlay G. Mills, Dayana Barker, Yurie Taya, Alexander W. Gofton, Iffy Glendinning, Amber Gillett, Peggy Rismiller, Yukari Horio, Aiko Ohnuma, Ernest J. M. Teo, Ryo Nakao, Stephen C. Barker

**Affiliations:** 1 Laboratory of Parasitology, Graduate School of Infectious Diseases, Faculty of Veterinary Medicine, Hokkaido University, Sapporo 060-0818, Japan; samuelkelava@gmail.com (S.K.); yukarihorio@vetmed.hokudai.ac.jp (Y.H.); ernestjmteo@gmail.com (E.J.M.T.); ryo.nakao@vetmed.hokudai.ac.jp (R.N.); 2 Department of Parasitology, School of Chemistry and Molecular Biosciences, The University of Queensland, Brisbane, QLD 4072, Australia; mingeun.cho@student.uq.edu.au (M.C.); finlay.mills@student.uq.edu.au (F.G.M.); dayana.barker@uqconnect.edu.au (D.B.); 3 Division of Risk Analysis and Management, International Institute for Zoonosis Control, Hokkaido University, Sapporo 001-0020, Japan; yurietaya@gmail.com; 4 CSIRO Health and Biosecurity, Brisbane, QLD 4001, Australia; alexander.gofton@csiro.au; 5 School of Veterinary Science, The University of Queensland, Gatton, QLD 4343, Australia; i.glendinning@uq.edu.au; 6 Australia Zoo Wildlife Hospital (AZWH), Beerwah, QLD 4519, Australia; amber@wildlifewarriors.org.au; 7 Pelican Lagoon Research & Wildlife Centre, Penneshaw, SA 5222, Australia; rismac@internode.on.net; 8 Technical Office, International Institute for Zoonosis Control, Hokkaido University, Sapporo 001-0020, Japan; aikoh@czc.hokudai.ac.jp; 9 Division of Parasitology, Veterinary Research Unit, International Institute for Zoonosis Control, Hokkaido University, Sapporo 001-0020, Japan; 10 One Health Research Centre, Hokkaido University, Sapporo 060-0818, Japan

**Keywords:** Acari, Ixodida, taxonomy, phylogeny, *Bothriocroton*, mitochondrial genome, species delimitation, integrative taxonomy, Australia

## Abstract

Background: We revised the taxonomy and identification of the species of the genus *Bothriocroton* in light of entire mitochondrial genomes and nuclear rRNA genes, scanning electron microscopy, and light microscopy. Methods: (i) phylogenetic trees inferred from entire mitochondrial genomes and nuclear rRNA genes, (ii) species delimitation analyses (ABGD and bPTP), and (iii) the study of morphology by scanning electron microscopy (SEM) and light microscopy. Results: The most complete picture of the *Bothriocroton* ticks so far. Thirty-five new mitochondrial genomes and 27 partial 18S–28S rDNA sequences were generated. Phylogenetic analyses recovered seven well-supported clades corresponding to species-level lineages. Intraspecific genetic divergence ranged from 0.14 to 1.16%, whereas interspecific divergence ranged from 4.94 to 21.75%. Three new species were described, and the morphological diagnosis of other species was improved by redescribing two other species. In addition, we updated and improved the morphological dichotomous keys to male and female ticks. Conclusions and prospects: The routine genotyping of *Bothriocroton* ticks to identify species and to confirm provisional identifications of species from their morphology is an alternative to the morphological identification of closely related *Bothriocroton* ticks.

## Simple Summary

For some closely related species of ticks, the fundamental task of identifying the species may be difficult. We used the mitochondrial and nuclear genomes of *Bothriocroton* ticks to refine their taxonomy and simplify the identification of these ticks to species. The present manuscript shows how these genomes are now being used to revise the taxonomy of ticks and thus to redescribe species, and to discover and describe new species of ticks. Here, 35 mitochondrial genome sequences are presented for the first time. We describe three new species: *Bothriocroton bulli* n. sp., *Bothriocroton dahlbergi* n. sp. and *Bothriocroton spratti* n. sp. and redescribe *Bothriocroton concolor* (Neumann, 1899) and *Bothriocroton hydrosauri* (Denny, 1843).

## 1. Introduction

Finding the boundaries between some species of ticks, solely on the basis of their morphology, varies from difficult to nigh impossible, even for the experts. For example, Bob Roberts, the father of Australian tick taxonomy [1,2] was confounded by the morphology of *Ixodes confusus* Roberts, 1960 and *I. cordifer* Neumann, 1908 [3], and *B. hydrosauri* (Roberts 1953 [4]) (but see a later publication, Roberts 1970 [5]). With this in mind, a decade or so ago we began a revision of the ticks of Australasia; we treated the species recognised by Bob Roberts [5] and recent authors as initial taxonomic hypotheses [6,7,8,9,10,11,12] to be tested. So we had 73 or so hypotheses of species of ticks on mainland Australia [13]; then we sought specimens spanning the known geographic range of each species. Here, we revise the genus *Bothriocroton*. Here is our version of the iterative process of taxonomy advocated by Yeates [14]:

1. Establish *prima facie* (H_0_) hypotheses of species boundaries from one source of data. We accepted as hypotheses the species of Bob Roberts (1970) [5] and recent authors, which were from traditional qualitative considerations of the morphology of adult ticks;
2. Test these species boundaries with results from a different dataset (in our case, entire mitochondrial (mt) genomes and nuclear rRNA genes) to produce H_1_;
3. If H_0_ and H_1_ are the same, the current species boundaries have survived the test, so iteration can stop. When new data become available, return to step 2;
4. If H_0_ and H_1_ indicate different species boundaries, search for a biological or evolutionary explanation to explain the differences;
5. Based on the results of step 4, refine species boundaries to produce a new hypothesis of species boundaries H_2_. The H_2_ species boundaries have survived testing, and iteration can end. Redescribe species that have survived the testing and describe new species. When new data become available, return to step 2.

Before the present revision, the genus *Bothriocroton* comprised seven extant species, all endemic to Australasia: *B. auruginans* Schulze, 1936, the wombat tick; *B. concolor* (Neumann, 1899), the echidna tick; *B. glebopalma* Keirans, King & Sharrad, 1994, no common name; *B. hydrosauri* (Denny, 1843), the southern reptile tick; *B. oudemansi* Neumann, 1910, the New Guinea echidna *Bothriocroton*; *B. tachyglossi* Roberts, 1953, the Central Qld short-beaked echidna tick; and *B. undatum* Fabricius, 1775, the goanna tick [5,15,16,17,18]. *Bothriocroton* is the sole genus of the subfamily Bothriocrotoninae of the clade Haematobothrion (*Haemaphysalis*, *Bothriocroton*, *Archaeocroton*; [19]). There is apparently a morphological synapomorphy for *Bothriocroton*—three pairs of large wax glands on segment VIII of larvae [18,20]. One extinct species of *Bothriocroton* is known—*Bothriocroton muelleri* from Burmese amber [21].

**Figure 1:**
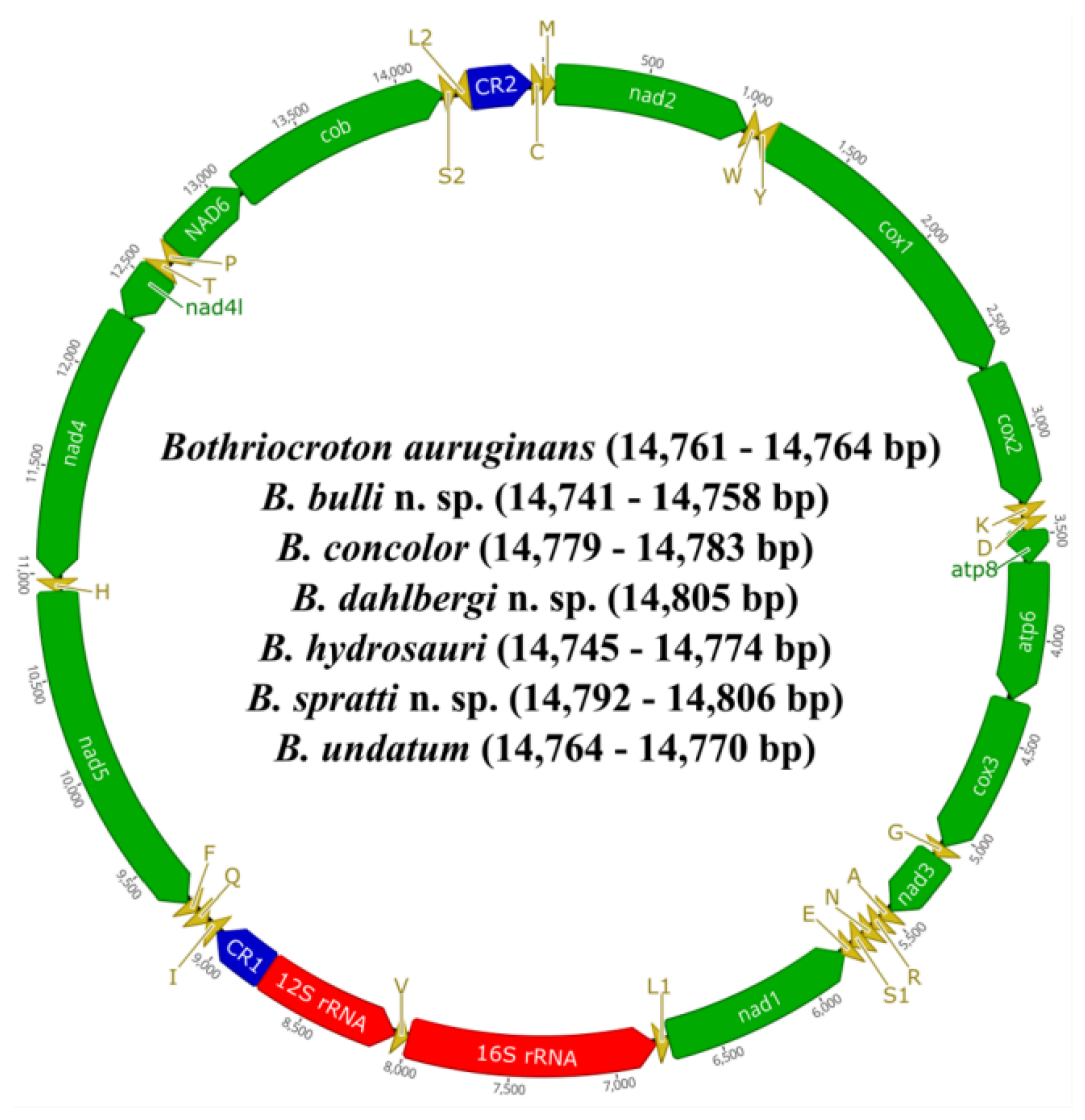
Mitochondrial genomes of the *Bothriocroton* species sequenced in the present project (refer to Table 1 for the NCBI GenBank numbers of the 35 genomes presented here for the first time together with the 11 genomes that were published previously, which we also studied in the present paper). Protein-coding genes are labelled with their four-character abbreviations, tRNAs are labelled with their one-letter amino-acid abbreviations whereas the control regions are labelled as CR1 and CR2. The range in size of the genomes of each species are indicated in brackets.

The present manuscript shows how entire mitochondrial genomes and nuclear rRNA sequences are now being used to revise the taxonomy of ticks and thus to redescribe species, and to discover and describe new species of ticks. Thirty-five new mitochondrial genome sequences are presented here for the first time (Figure 1). We describe three new species, *B. bulli* n. sp., *B. dahlbergi* n. sp. and *B. spratti* n. sp., and redescribe the sister species of these new species, *B. concolor* (Neumann, 1899) and *B. hydrosauri* (Denny, 1843).

**Table 1:** Material examined.

Genus	Species	Sample ID	Mitochondrion NCBI Accession	Nuclear rRNA NCBI Accession	QM Registration Number	Locality	State	Latitude	Longitude	Altitude (m)	Collector	Date Collected	Life Stage	Host Sci Name	MT Genome Mean Coverage	18S–28S Block Mean Coverage
*Bothriocroton*	*auruginans*	B7014.F1	PZ836780	PZ835940	na	Buffalo	Vic	−38.6460	146.0310	90	Claire O’Brien, Kate Johnson and colleagues	14.09.2022	Female	*Vombatus ursinus*	267.202	3012.514
*Bothriocroton*	*auruginans*	B7014.F2	PZ836781	PZ835941	na	Buffalo	Vic	−38.6460	146.0310	90	Claire O’Brien, Kate Johnson and colleagues	14.09.2022	Female	*Vombatus ursinus*	832.498	5995.908
*Bothriocroton*	*auruginans*	B7014.N1	PZ836782	PZ835942	na	Buffalo	Vic	−38.6460	146.0310	90	Claire O’Brien, Kate Johnson and colleagues	14.09.2022	Nymph	*Vombatus ursinus*	115.806	1736.771
*Bothriocroton*	*auruginans*	B7014.N3	PZ836783	PZ835943	na	Buffalo	Vic	−38.6460	146.0310	90	Claire O’Brien, Kate Johnson and colleagues	14.09.2022	Nymph	*Vombatus ursinus*	30.374	413.441
*Bothriocroton*	*auruginans*	B7285.F2	PZ836784	PZ835944	na	Waratah Bay	Vic	−38.8030	146.0370	8	Heidi Farrer, Calypso Tuttle and colleagues	28.05.2023	Female	*Vombatus ursinus*	288.167	6830.551
*Bothriocroton*	*auruginans*	B6841	PZ836779	PZ835939	na	St Helens (East Coast Veterinary Clinic)	Tas	na	na	na	Andrew Byrne	22.11.2021	Female	*Vombatus ursinus*	16.17	133.58
*Bothriocroton*	*auruginans*	B3401	na	na	na	Kalimna West	Vic	−37.847469	147.9564	68	Sean Redd	na	Male and Female	*Canis familiaris*	na	na
*Bothriocroton*	*auruginans*	B3349	na	na	na	Mansfield	Vic	−37.052094	146.087744	320	na	20.02.2015	Male and Female	*Vombatus ursinus*	na	na
*Bothriocroton*	*bulli*	B9105	na	na	na	Young River	WA	−33.6847	121.0798	51	Stephen and Dayana Barker	12.10.2015	Male	*Tiliqua rugosa*	na	na
*Bothriocroton*	*bulli*	B4931	PZ836787	PZ835946	S124262	Boxwood Hill, Bremer Bay	WA	−34.361	118.745	75	Stephen and Dayana Barker	10.2015	Female	*Tiliqua rugosa*	252.862	653.844
*Bothriocroton*	*bulli*	B4922.M	PZ836786	na	S124260	Pemberton	WA	−34.4430	116.0350	175	Stephen and Dayana Barker	01.10.2015	Male	*Tiliqua rugosa*	11.671	na
*Bothriocroton*	*bulli*	B4922	na	na	S124261	Pemberton	WA	−34.4430	116.0350	175	Stephen and Dayana Barker	01.10.2015	Male and Female	*Tiliqua rugosa*	11.671	na
*Bothriocroton*	*bulli*	B4918	PZ836785	PZ835945	S124263	Margaret River	WA	−33.9530	115.0730	90	Stephen and Dayana Barker	10.10.2015	Female	*Tiliqua rugosa*	1168.568	955.819
*Bothriocroton*	*concolor*	B8484	PZ836793	PZ835951	na	Gatton	Qld	−27.55435	152.2393	94	Iffy Glendinning	19.08.2024	Female	*Tachyglossus aculeatus*	28.811	135.558
*Bothriocroton*	*concolor*	B8145.1F	PZ836792	PZ835950	na	Nutgrove, via Dalby	Qld	−27.04994	151.7492	470	Craig J. Tan	16.09.2023	Female	*Tachyglossus aculeatus*	990.946	5048.539
*Bothriocroton*	*concolor*	B8060	na	na	na	Beerwah (AZWH)	Qld	na	na	na	Amber Gillett and Ludovica Valenza	21.07.2023	Male and Female	*Tachyglossus aculeatus*	na	na
*Bothriocroton*	*concolor*	B7019.F10	PZ836791	na	na	Beerwah (AZWH)	Qld	na	na	na	Amber Gillett and Ludovica Valenza	2021	Female	*Tachyglossus aculeatus*	2.005	na
*Bothriocroton*	*concolor*	B6868.3F	PZ836790	PZ835949	na	Pinkett via Glen Innes	NSW	−29.9600	151.9800	1060	Heather and Tristan Constantini	11.01.2019	Female	*Tachyglossus aculeatus*	930.245	4176.993
*Bothriocroton*	*concolor*	B3095	PZ836789	PZ835948	na	Minden	Qld	−27.5590	152.5450	95	Stephen and Dayana Barker	06.10.2014	Male	*Tachyglossus aculeatus*	156.279	1843.358
*Bothriocroton*	*concolor*	B3007	na	na	na	Mt Ommaney	Qld	−27.54911	152.918	35	unknown	18.08.2002	Male and Female	*Tachyglossus aculeatus*	na	na
*Bothriocroton*	*concolor*	B0895	PZ836788	PZ835947	na	PNG	PNG	na	na	na	Geordie Torr	03.12.1996	Male	*Varanus chlorostigma*	218.343	1097.006
*Bothriocroton*	*dahlbergi*	B7139.F1	PZ836794	PZ835952	S124264	Mt Molloy (Mulligan Hwy 4 km Sth of Mt Molloy)	Qld	−16.7030	145.3310	360	Amber Dahlberg	19.01.2023	Female	*Tachyglossus aculeatus*	320.109	958.618
*Bothriocroton*	*dahlbergi*	B7139	na	na	S124265	Mt Molloy (Mulligan Hwy 4 km Sth of Mt Molloy)	Qld	−16.7030	145.3310	360	Amber Dahlberg	19.01.2023	Female	*Tachyglossus aculeatus*	320.109	958.618
*Bothriocroton*	*dahlbergi*	B3148	na	na	na	Captain Billy Ck	Qld	−11.66	142.85	8	unknown	09.07.1975	Male	*Tachyglossus aculeatus*	na	na
*Bothriocroton*	*hydrosauri*	B9104	na	na	na	Emu Park	Qld	−23.25	150.8167	17	N. Chilton and Ian Beveridge	04.07.1992	Male and Female	*Tachyglossus aculeatus*	na	na
*Bothriocroton*	*hydrosauri*	B8473	na	na	na	St Helens (East Coast Veterinary Clinic)	Tas	na	na	na	Andrew Byrne	08.12.2023	Male, Nymph and Larvae	*Tiliqua nigrolutea*	na	na
*Bothriocroton*	*hydrosauri*	B8405.1F	PZ836802	PZ835959	na	Wattle Bank	Vic	−38.57637	145.69683	73	Heidi Farrer, Calypso Tuttle and colleagues	25.03.2024	Female	*Tiliqua scincoides*	810.213	2219.436
*Bothriocroton*	*hydrosauri*	B8395.M2	PZ836801	PZ835958	na	Damascus via Gin Gin	Qld	−24.94055	151.93388	90	Stephen Barker and Andy Sides	14.04.2024	Male	*Tachyglossus aculeatus*	655.794	4246.255
*Bothriocroton*	*hydrosauri*	B8394.M2	PZ836800	PZ835957	na	East End (via Calliope)	Qld	−23.87889	151.015	120	Stephen Barker and Andy Sides	14.04.2024	Male	*Tachyglossus aculeatus*	1224.552	9755.241
*Bothriocroton*	*hydrosauri*	B7125.M1	PZ836798	na	na	Phillip Is	Vic	−38.48418	145.26056	40	Katie Moon	2016	Male	*Tiliqua scincoides*	209.774	na
*Bothriocroton*	*hydrosauri*	B7125.M2	PZ836799	PZ835956	na	Phillip Is	Vic	−38.48418	145.26056	40	Katie Moon	2016	Male	*Tiliqua scincoides*	53.113	673.632
*Bothriocroton*	*hydrosauri*	B6930	na	na	na	Adelaide	SA	−35.07364	138.49695	na	Gerrut Norval	10.10.2021	Male and Female	*Tiliqua scincoides*	na	na
*Bothriocroton*	*hydrosauri*	B6695	OR350529	PZ835955	na	Salford Rd, Bundey	SA	−33.92963	139.28253	90	Mike Gardner and Sophie Hammond	01.03.2021	Male	*Tiliqua rugosa*	na	927.873
*Bothriocroton*	*hydrosauri*	B4210C	na	na	na	Woodend (Wombat State Forest)	Vic	−37.405336	144.497503	670	Devina Zoekita	05.02.2016	Female	*Tiliqua scincoides*	na	na
*Bothriocroton*	*hydrosauri*	B3165	PZ836796	PZ835954	na	West Launceston	Tas	−41.4490	147.1320	95	Rachel Bartlett, Amanda Chong and Colleagues	26.11.2014	Female	*Tiliqua nigrolutea*	65.528	84.939
*Bothriocroton*	*hydrosauri*	B0643.M1	PZ836795	PZ835953	na	Flinders Is	Tas	na	na	na	Ashley Edwards	21.02.1996	Male	*Tiliqua nigrolutea*	878.59	382.599
*Bothriocroton*	*hydrosauri*	B7113.F1	PZ836797	na	na	Little Desert Ntn Pk	Vic	−36.5350	141.7520	135	Stephen and Dayana Barker	2020	Female	*Tiliqua rugosa*	164.623	na
*Bothriocroton*	*spratti*	B8632	PZ836809	PZ835961	na	Albury/Wodonga	NSW	na	na	na	Leo Lee, Sancha Barrett and Co.	23.09.2024	Female	*Tachyglossus aculeatus*	20.765	423.403
*Bothriocroton*	*spratti*	B8215	na	na	na	Wonthaggi	Vic	−38.60484	145.59096	42	Heidi Farrer, Calypso Tuttle and colleagues	13.11.2023	Male and Female	*Tachyglossus aculeatus*	na	na
*Bothriocroton*	*spratti*	B8141	PZ836808	PZ835960	S124268	Phillip Is	Vic	−38.48418	145.26056	40	Heidi Farrer, Calypso Tuttle and colleagues	9.2023	Female	*Tachyglossus aculeatus*	783.511	2421.513
*Bothriocroton*	*spratti*	B6897	PZ836807	na	Na	Hindmarsh Valley (vicinity of Adelaide)	SA	−35.46813	138.64245	80	Mathew Shaw	10.01.2019	Female	*Tachyglossus a. multiaculeatus*	5771.732	na
*Bothriocroton*	*spratti*	B5485B	PZ836806	na	Na	Kangaroo Is	SA	na	na	na	Peggy Rismiller	26.03.2020	Male and Female	*Tachyglossus a. multiaculeatus*	453.943	na
*Bothriocroton*	*spratti*	B5481	PZ836805	na	Na	Kangaroo Is	SA	na	na	na	Peggy Rismiller	04.08.2018	Female	*Tachyglossus a. multiaculeatus*	91.017	na
*Bothriocroton*	*spratti*	B5478	PZ836804	na	Na	Kangaroo Is	SA	na	na	na	Peggy Rismiller	07.01.2019	Female	*Tachyglossus a. multiaculeatus*	91.589	na
*Bothriocroton*	*spratti*	B5461.M	PZ836803	na	S124266	Woodend	Vic	−37.35866	144.52747	570	James, Dyer, Luxmore, Robson and colleagues	26.08.2019	Male	*Tachyglossus aculeatus*	932.96	na
*Bothriocroton*	*spratti*	B5461	na	na	S124267	Woodend	Vic	−37.35866	144.52747	570	James, Dyer, Luxmore, Robson and colleagues	26.08.2019	Male	*Tachyglossus aculeatus*	932.96	na
*Bothriocroton*	*spratti*	B2296	NC_017756	na	na	Kangaroo Is	SA	na	na	na	Ian Beveridge	06.07.1993	Female	*Tachyglossus a. multiaculeatus*	na	na
*Bothriocroton*	*undatum*	B8356B	PZ836813	PZ835965	na	Happy Valley Dr, K’gari (Fraser Is)	Qld	−25.33831	153.2010	5	Amber Gillett and Ludovica Valenza	02.09.2023	Female	*Varanus varius*	587.873	3897.243
*Bothriocroton*	*undatum*	B8140B.M1	PZ836812	PZ835964	na	Gatton (UQ-VSH)	Qld	na	na	na	Iffy Glendinning	9.2023	Male	*Varanus varius*	23.05	1129.424
*Bothriocroton*	*undatum*	B5053A.N1	PZ836811	PZ835963	na	Sandringham	Vic	−37.9520	145.0120	17	unknown	05.12.2018	Nymph	*Varanus varius*	33.796	135.09
*Bothriocroton*	*undatum*	B4026a	na	na	na	Mudgee	NSW	−32.5989	149.5872	467	unknown	21.12.2015	Female	*Varanus varius*	na	na
*Bothriocroton*	*undatum*	B3263	PZ836810	PZ835962	na	Genoa	Vic	−37.45	149.583	35	Stephen and Dayana Barker	09.02.2015	Male	*Varanus varius*	539.606	2669.918
*Bothriocroton*	*undatum*	B2313	NC_017757	na	na	Fraser Is (Dundubara camping area)	Qld	−25.1644	153.2819	10	Stephen, Maxwell and Thomas Barker	12.04.2009	Male	*Varanus gouldi*	na	na

## 2. Materials and Methods

### 2.1. Material Studied

Only field-collected ticks were available for study (Table 1).

### 2.2. Microscopy Methods

Ticks were examined using a stereoscopic microscope (Nikon SMZ800N, Nikon Corp., Tokyo, Japan, and Olympus SZX16, Olympus Corp., Tokyo, Japan), compound microscope (Olympus BX53, Olympus Corp., Tokyo, Japan) and scanning electron microscope (Hitachi TM4000, Hitachi High-Tech Corp., Tokyo, Japan). An ocular micro-meter was used to measure ticks. Measurements are in millimetres. Colour digital images were taken with an Olympus DSX1000 (Olympus Corp., Tokyo, Japan). Adobe Photoshop^®^ software v27.10.0 was used to polish and sharpen images. Measurements were taken from holotype or paratype specimens where applicable.

### 2.3. Sequencing and Assembly of Mitochondrial Genomes and Nuclear rRNA

Ticks were sequenced by three approaches. First, DNA was prepared at CSIRO, Australia, where 24 ticks were sequenced. DNA was extracted with the QIAGEN DNeasy Blood and Tissue Kit (Qiagen, Hilden, Germany), following initial digestion with Proteinase K at 56 °C for 16 h. DNA concentration was measured with the Qubit dsDNA Broad Range Kit. Paired-end sequencing libraries were prepared from genomic DNA with the Illumina DNA Prep Kit (Illumina, Hayward, CA, USA) and sequenced on an Illumina NovaSeq 6000 platform at the Genome Discovery Unit—ACRF Biomolecular Resource Facility, John Curtin School of Medical Research, Australian National University.

Second, seven ticks were sequenced at Hokkaido University, Japan. DNA was extracted by the blackPREP Tick DNA/RNA kit (Analytik Jena, Germany). The DNA was normalized to a concentration of 0.2 ng/µL before library preparation. Indexed paired-end shotgun libraries were constructed using the Nextera XT DNA Library Prep Kit (Illumina, Hayward, CA, USA) and sequenced on an Illumina MiSeq platform with the MiSeq Reagent Kit v3 (600 cycles).

Third, DNA from four ticks was prepared at the University of the Sunshine Coast, Australia. Genomic DNA was extracted with the QIAGEN DNeasy Blood and Tissue kit. Ticks were bisected and lysed for 24 h with Proteinase K at 56 °C. The quantity of DNA was measured with Nanodrop and Qubit instruments; only samples with at least 200 ng of DNA were sequenced. DNA was sent to Novogene Singapore for library preparation and sequencing on the Illumina Nova-seq 6000 platform.

Illumina reads underwent standard platform-based quality filtering prior to assembly, and residual adapter sequences were removed using Trimmomatic with the TruSeq3 paired-end adapter file. Geneious Prime build 2022-11-28 (Geneious assembler) was used to assemble mt genome and nuclear rRNA genes [22].

### 2.4. Annotation of Mitochondrial Genomes

Mitochondrial genomes were annotated with Geneious Prime. Protein-coding genes were identified by searches with BLAST v2.12 [23] for open reading frames. Regions between protein-coding genes were searched with BLAST [23] to find rRNA genes, tRNA genes and control regions. The tRNA annotations were checked with the aid of the tRNAscan-SE Search Server v1.21 [24] and the MITOS Web Server [25]. The nucleotide sequences of tRNA genes were confirmed by studying the putative secondary structures of transcripts, as implemented in Geneious Prime [22].

### 2.5. Phylogenetic Methods

The 13 mitochondrial protein-coding genes and nuclear rRNA genes (18S, ITS1, ITS2, and 28S) were aligned separately using MAFFT with the FFT-NS-i ×1000 algorithm, a 100PAM/K2 scoring matrix, a gap opening penalty of 1.57, and an offset value of 0.123 [26]. Mitochondrial protein-coding genes were aligned according to an amino acid translation and then back-translated to nucleotides. All alignments were trimmed using TrimAl with the automated1 algorithm [27]. For the ITS1 and ITS2 alignments, nucleotide positions that had alignment gaps in more than 40% of sequences were removed. Regions of ambiguous alignment were manually inspected and excluded prior to analysis. No evidence of intragenomic polymorphism or paralogous sequence variants was detected in the assembled nuclear rRNA sequences. The optimal partitioning scheme and substitution models for each dataset were determined with Partition Finder v2.1.1 [28]. For the mitochondrial nucleotide dataset, codon positions were initially partitioned separately for each gene and subsequently merged into 20 subsets according to the PartitionFinder results (Table S1). The mitochondrial amino acid dataset was partitioned into five subsets (Table S1). The nuclear dataset was partitioned by locus (18S, ITS1, ITS2, and 28S); each locus was assigned a substitution model (Table S1).

Phylogenetic relationships were inferred by both Maximum Likelihood (ML) and Bayesian Inference (BI) approaches. ML analyses were undertaken with IQ-TREE v2.1.3 [29,30] whereas BI analyses were undertaken with MrBayes v3.2.6 [31]. The mitochondrial genome phylogeny was inferred from the concatenated 13 mitochondrial protein-coding genes (ca. 10,553 bp), whereas the nuclear phylogeny was inferred from the concatenated 18S rRNA, ITS1, ITS2 and 28S rRNA genes (ca. 7510 bp). The 18S, ITS1, ITS2, and 28S regions were analysed as a single nuclear rDNA dataset because they are a contiguous ribosomal DNA cistron, and they were obtained from the same genomic region. To accommodate differences in evolutionary rate and substitution patterns among regions, the nuclear rRNA dataset was partitioned by locus and independent substitution models were applied to each partition. Separate phylogenetic analyses were conducted for the partial *cox1* (515 bp), 16S rRNA (425 bp), 12S rRNA (378 bp), and combined ITS1 + ITS2 (3003 bp) genes in addition to the concatenated datasets. For each marker, alignments were trimmed to lengths corresponding to those of previously published sequences to maximize taxon inclusion. For ML analyses, branch support was assessed by 1000 ultrafast bootstrap replicates [32]. BI analyses were run with one cold and three heated chains for 10 million generations or until the average standard deviation of split frequencies was below 0.01, sampling every 1000 generations, with the first 25% of samples discarded as burn-in (Table S2). Convergence and effective sample sizes were evaluated in Tracer v1.7 [33]. Phylogenetic trees and associated support values (bootstrap and posterior probabilities) were visualized in Fig Tree v1.4.4 [34]. Sequences from the *Alloceraea, Archaeocroton* and *Haemaphysalis* genera were designated as the outgroup (sensu [19]).

### 2.6. Pairwise Genetic Differences and Species Delineation Methods (Gap Analyses)

The mitochondrial genomes were aligned to generate matrices of pairwise genetic differences (%) and Tamura–Nei genetic distances, the latter calculated in MEGA11 [35]. Species delimitation was from the mitochondrial genome dataset by two approaches: (i) Automatic Barcode Gap Discovery [36] and (ii) Poisson Tree Processes [37]. The species delimitation analyses were undertaken with the implementation available through iTaxoTools [38], applying default parameters (Pmin = 0.001, Pmax = 0.1, steps = 10, X = 1.5). Genetic distances for this analysis were computed with the Kimura two-parameter (K80) model (TS/TV = 2.0). The Poisson Tree Processes method was also implemented with iTaxoTools, using its Bayesian formulation (bPTP), which was applied to the phylogenetic tree previously inferred with IQ-TREE. To evaluate the potential influence of outgroup species on branching-rate estimates, bPTP analyses were conducted both with and without outgroup sequences; both analyses recovered identical species delimitation results.

Since ABGD and bPTP were both based on mitochondrial genome data, multilocus analyses were subsequently used to assess whether the mtDNA-derived species hypotheses were supported by nuclear evidence. Species relationships were inferred under the multispecies coalescent using StarBEAST2 implemented in BEAST 2 vers 2.7.7 [39,40]. Independent loci were defined for the mitochondrial genome (13 concatenated protein-coding genes) and the nuclear rRNA region (18S, ITS1, ITS2 and 28S), each assigned a separate substitution and clock model. Sequence evolution for both loci was modelled using the General Time Reversible (GTR) substitution model with gamma-distributed among-site rate heterogeneity (four discrete gamma categories), while the proportion of invariant sites was fixed at zero. The gamma shape parameter was estimated independently for each locus. Uncorrelated lognormal relaxed clocks were applied to each locus with independent rate parameters. Species tree estimation employed a Yule speciation prior, with the speciation rate assigned a uniform prior. Population sizes were modelled using the Linear-with-Constant-Root (LWCR) population model implemented in StarBEAST2, with tip population sizes assigned lognormal priors and ancestral population sizes assigned gamma-distributed priors. Two independent runs were executed to assess convergence. Convergence and mixing were evaluated in Tracer v1.7 [33], ensuring effective sample sizes (ESS) exceeded 200 for all parameters. Log and tree files from independent runs were combined after the removal of 10–20% burn-in using LogCombiner 2.7.7 [40], and a maximum clade credibility tree was generated using TreeAnnotator 2.7.7 [40].

To evaluate species boundaries under the multispecies coalescent, Bayesian species delimitation analyses were executed in the Bayesian Phylogenetics and Phylogeography program (BPP v4.8.7) under the multispecies coalescent model [41,42]. Analyses were executed using the same two loci employed in the StarBEAST2 analyses: the concatenated mitochondrial protein-coding genes (10,553 bp) and the nuclear rRNA dataset (7510 bp). A fixed guide tree corresponding to the StarBEAST2 topology was specified, and species delimitation was executed using the A11 analysis (joint species delimitation and species tree estimation with a fixed guide tree). Prior distributions for population size parameters (θ) and divergence times (τ) were initially estimated using A00 analyses. Two independent A00 runs were conducted using different random seeds (1234 and 5678) to assess the reproducibility of parameter estimates. The analyses were executed using inverse-gamma priors of θ ~ IG(3, 0.002) and τ ~ IG(3, 0.2), with fine-tuning parameters set to Gage:0.02, Gspr:0.02, tau:0.02, mix:0.02, lrht:0.02, phis:0.02, and pi:0.02. Both runs produced highly similar posterior estimates and were used to guide the selection of biologically realistic prior distributions for subsequent species delimitation analyses. To evaluate the sensitivity of species delimitation, six A11 analyses were executed with combinations of inverse-gamma priors for θ [IG(3,0.002), IG(3,0.005), and IG(3,0.010)] and τ [IG(3,0.02) and IG(3,0.05)]. Each analysis had 500,000 MCMC samples following a burn-in of 20,000 samples, with parameters recorded every five iterations. The species model prior was set to speciesmodelprior = 1, and all other parameters were held constant in our analyses. Posterior probabilities of alternative species-delimitation models were compared among prior combinations to assess the robustness of species boundaries to prior assumptions.

## 3. Results

### 3.1. Mitochondrial Genomes

Here, 35 entire mitochondrial genomes are presented for the first time. These mitochondrial genomes had the typical gene arrangement of 37 genes of the mt genomes of metastriate ticks (Figure 1).

### 3.2. Phylogenies Inferred from Mitochondrial Genomes and Nuclear rRNA

**Figure 2:**
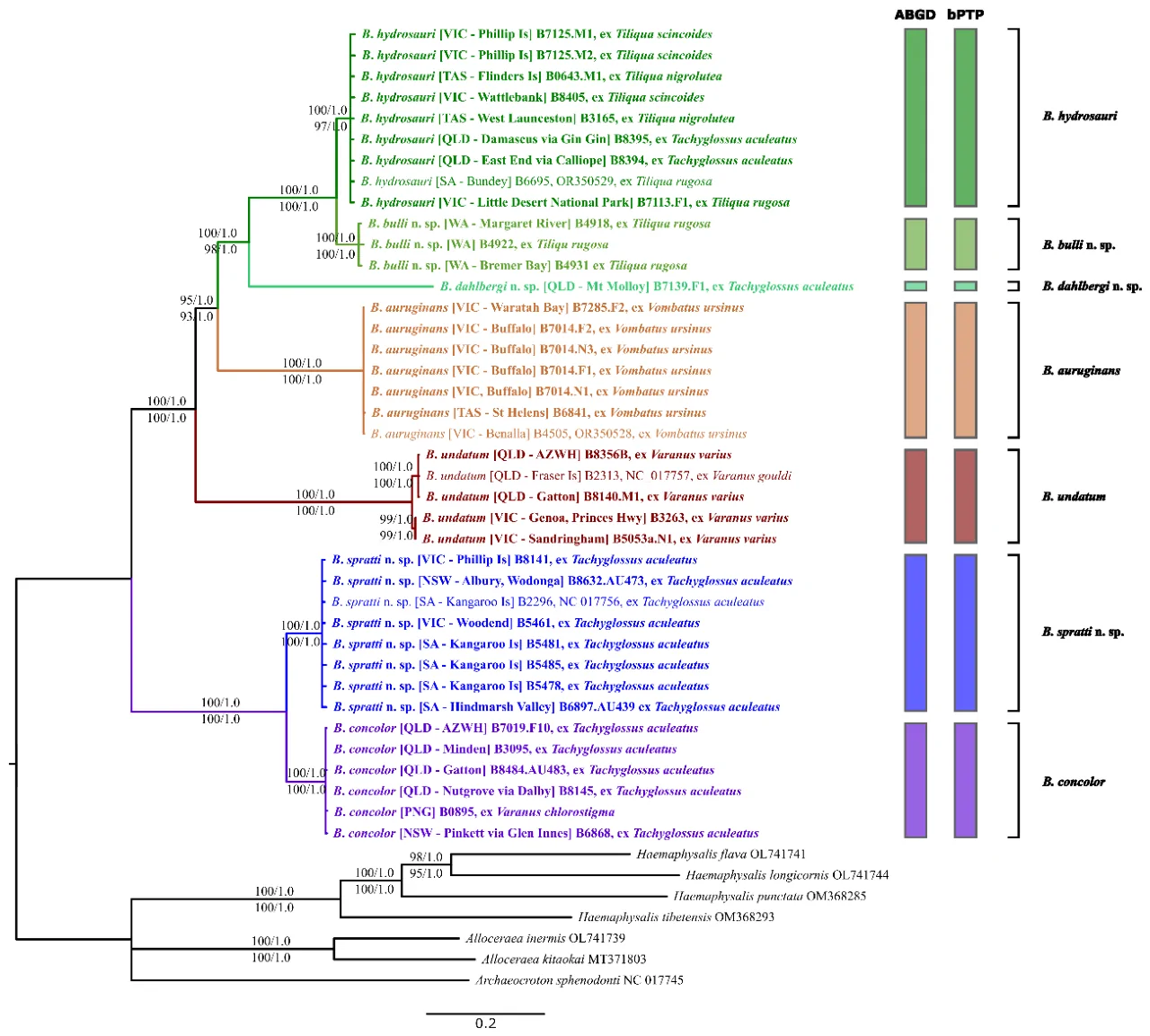
Phylogenetic tree inferred from 46 nucleotide and amino acid sequences (39 *Bothriocroton* sequences plus 7 *Haemaphysalis*, 2 *Alloceraea* and 1 *Archaeocroton* sequences) derived from the 13 protein-coding genes of the mitochondrial (mt) genome: *atp6*, *atp8*, *cox1*, *cox2*, *cox3*, *cytb*, *nad1*, *nad2*, *nad3*, *nad4*, *nad4l*, *nad5* and *nad6* These sequences were concatenated into alignments of 10,553 bp and 3512 amino acids, respectively. Maximum Likelihood (ML) bootstrap support and Bayesian Inference (BI) posterior probabilities for the nucleotide dataset, respectively, are shown above branches, whereas bootstrap and posterior probability support, respectively, for the amino acid dataset, are shown below branches. The species predicted by the ABGD and bPTP species-delimitation models are indicated by the coloured columns on the right-hand side. Bold font indicates the 35 mt genome sequences published for the first time in the present paper. Abbreviations: Qld, Queensland; NSW, New South Wales; Vic, Victoria; WA, Western Australia; SA, South Australia; Tas, Tasmania; PNG, Papua New Guinea.

Phylogenies from the mt 13 protein-coding genes of the mitochondrial genome had seven main clades in both the DNA and amino-acid trees (Figure 2). All seven clades had >95% bootstrap support and 1.0 posterior probabilities in ML and BI trees, respectively (Figure 2). Ticks initially identified by their morphology as *B. hydrosauri* (Denny, 1843), from WA (Margaret River, B4918; Pemberton, B4922; Bremer Bay, B4931), formed a distinct clade separate from all other *B. hydrosauri*, which were in a clade comprising ticks from Vic, Tas, SA and Qld (Figure 2). In a similar manner, ticks initially identified as *B. hydrosauri* (Denny, 1843), from Far North Qld (Mt Molloy, B7139), did not cluster with any other ticks, instead forming a sister lineage to all of the other *B. hydrosauri* (the *B. hydrosauri* group, Figure 2). Ticks initially identified by their morphology as *B. concolor* (Neumann, 1899) also resolved into more than one well-supported clade: one clade comprising eight samples from SA, NSW, and Vic, and a second comprising six samples from Qld, NSW, and Papua New Guinea (PNG) (the *B. concolor* group, Figure 2).

**Figure 3:**
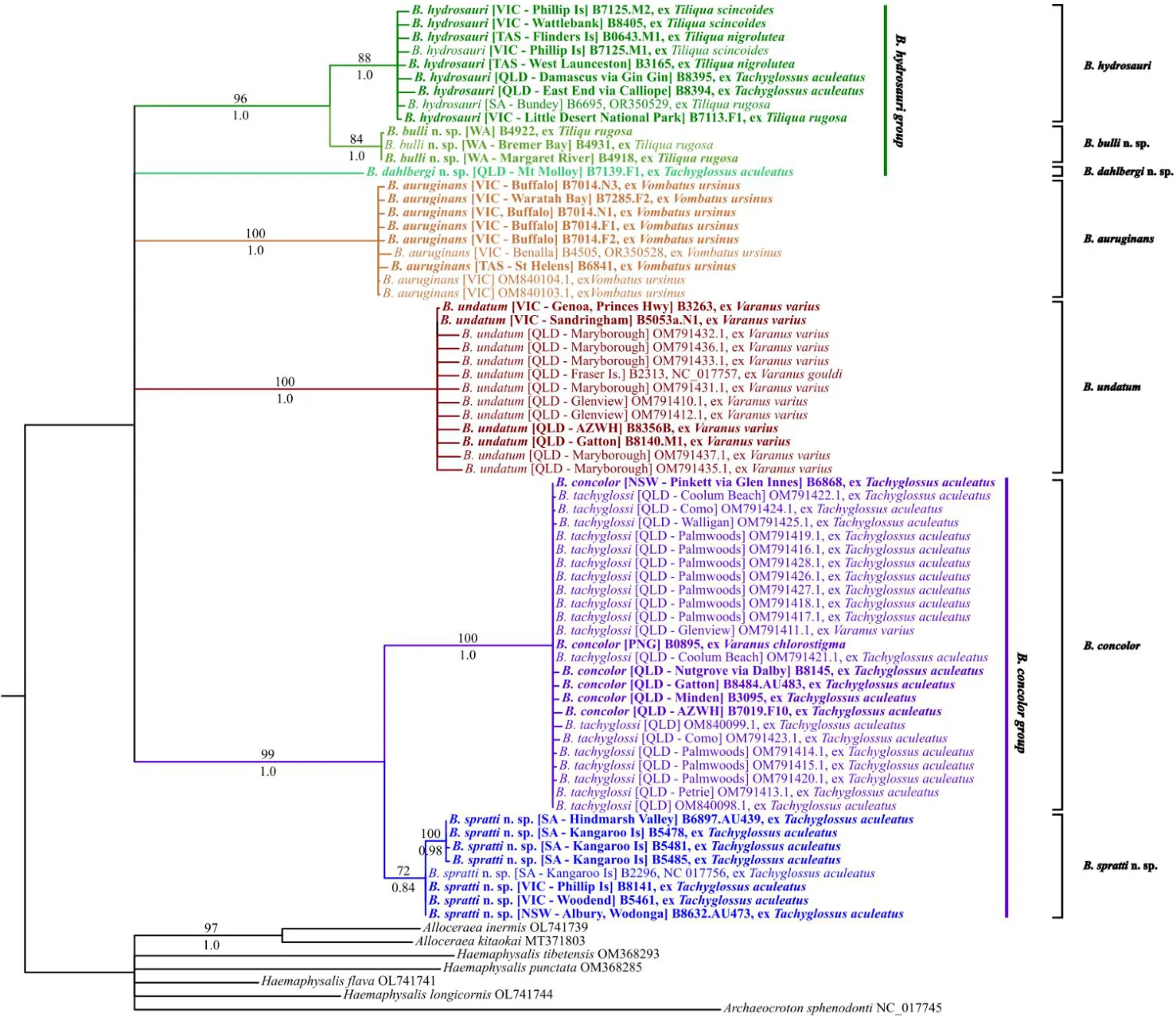
Phylogenetic tree inferred from 75 partial nucleotide sequences of mitochondrial *cox1*. The final alignment was 515 bp. Maximum Likelihood (ML) bootstrap support is shown above branches, whereas Bayesian Inference (BI) posterior probability supports are below branches. Coloured fonts and labels indicate the species-level clades that were inferred from the mitochondrial genome tree (Figure 2). Bold font indicates the 35 mitochondrial genome sequences published in the present paper. Abbreviations: Qld, Queensland; NSW, New South Wales; Vic, Victoria; WA, Western Australia; SA, South Australia; Tas, Tasmania; PNG, Papua New Guinea.

The tree inferred from partial *cox1* nucleotides (515 bp) had moderate to high bootstrap and posterior probability supports (>83% ML bootstrap, 1.0 BI posterior probability) for six of the seven clades (Figure 3). Only the support for the *B. concolor* clade that had ticks from NSW, SA and Vic was weak (72% ML, 0.84 BI). Phylogenetic relationships among the species-level clades were not resolved. The sequences from NCBI GenBank labelled as *B. tachyglossi* from Qld (NCBI GenBank accession numbers: OM791411, OM791413-OM791428, OM840098 and OM840099) were all in a clade with the *B. concolor* sequences from Qld, NSW and PNG. These sequences should be relabelled in GenBank as *B. concolor* (Neumann, 1899). Trees inferred from partial 12S and 16S rRNA sequences had nine well-supported species-level clades (ML bootstrap > 94%; BI posterior probability > 0.94), with the exception of the *B. hydrosauri* clade, which had weak support in the 12S tree (67% ML; 0.54 BI) and moderate to high support in the 16S tree (100% ML; 0.73 BI) (Figures S1 and S2). In both of these trees, *B. glebopalma* was the sister to *B. undatum*. In the 12S rRNA phylogeny, *B. oudemansi* was the sister to the *B. glebopalma* plus *B. undatum* clade (Figure S2).

**Figure 4:**
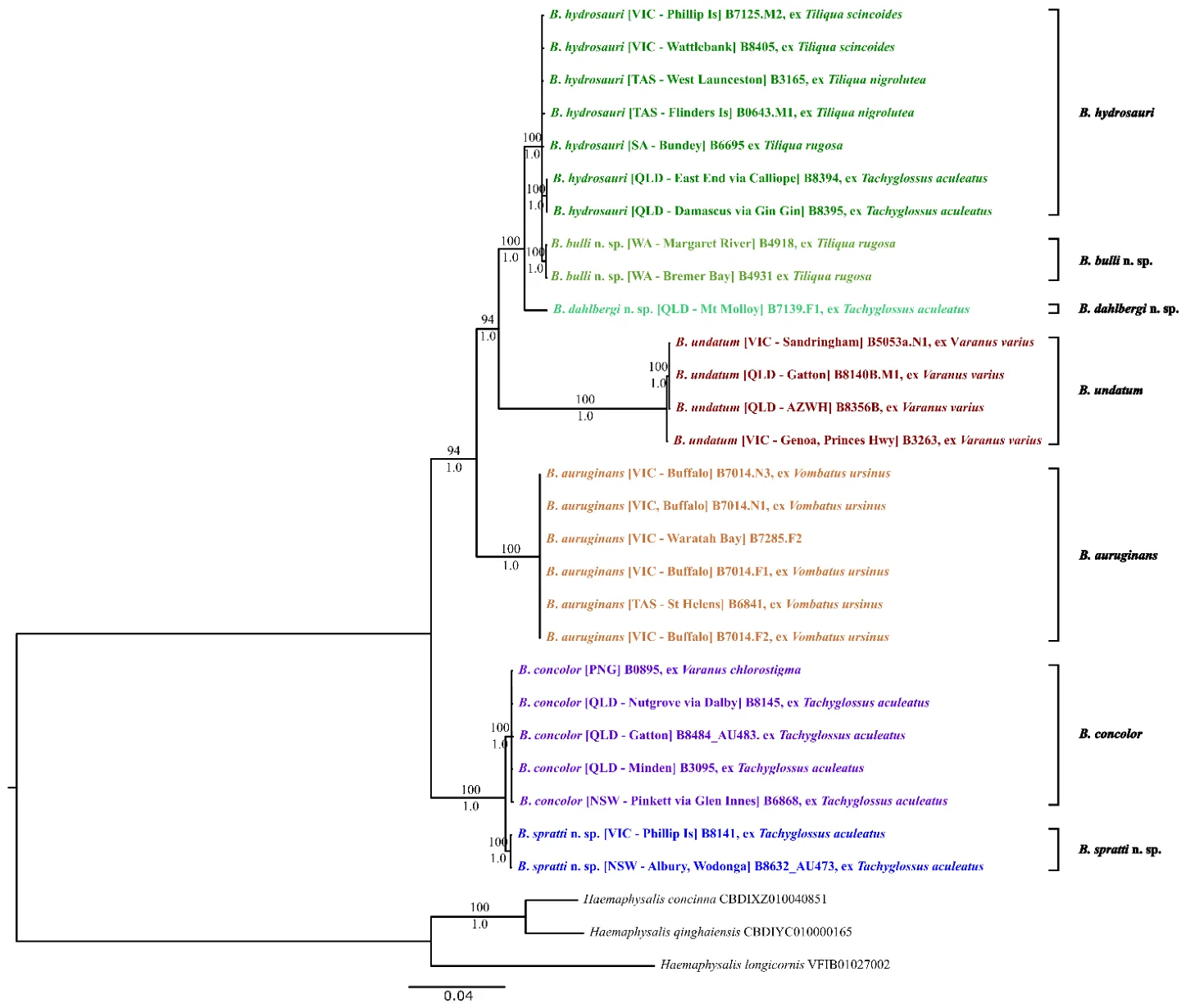
Phylogenetic tree inferred from 30 nucleotide sequences of 18S rRNA, ITS1, ITS2 and 28S rRNA. These DNA sequences were concatenated into an alignment of 7510 bp. Maximum Likelihood (ML) bootstrap support is above branches whereas Bayesian Inference (BI) posterior probability support is shown below branches. Coloured fonts and labels indicate the clades inferred from the mt genome tree (Figure 2). The bold font indicates the 27 rRNA sequences published for the first time in the present paper. Abbreviations: Qld, Queensland; NSW, New South Wales; Vic, Victoria; WA, Western Australia; SA, South Australia; Tas, Tasmania; PNG, Papua New Guinea.

Phylogenetic analyses of the concatenated 18S, ITS1, ITS2, and 28S nuclear genes (7510 bp) yielded six well-supported clades (100% ML bootstrap, 1.0 BI posterior probability) (Figure 4). Ticks identified initially by their morphology as *B. hydrosauri* from Far North Qld (Mt Molloy, B7139) formed a clade separate to all other *B. hydrosauri* (*B. hydrosauri* group, Figure 4). Ticks identified initially by their morphology as *B. hydrosauri* from WA (Margaret River, B4918; Bremner Bay, B4931) clustered together in a strongly supported clade (100% ML, 1.0 BI) (Figure 4). As for the mt 13 protein-coding gene tree, the tree inferred from 18S, ITS1, ITS2, and 28S also had two main clades of *B. concolor* (The B. concolor group) (Figure 4). *B. undatum* was the sister to the *B. hydrosauri* group (94% ML, 1.0 BI) in the 18S rRNA, ITS1, ITS2 and 28S rRNA tree, whereas *B. auruginans* was the sister to the *B. hydrosauri* group (93–95% ML, 1.0 BI) in the 13 mt protein-coding gene tree (Figure 2). A separate phylogenetic analysis of the ITS1 and ITS2 regions was largely congruent with the combined nuclear rRNA tree (Figure S3).

### 3.3. Genetic Difference and Species Delineation

Mean pairwise genetic difference (p-distance) among ticks from *within* the seven species-level clades from entire mitochondrial genomes varied from 0.14% to 1.16% (Table 2). In contrast, the mean genetic difference (p-distance) *among* the seven species-level clades varied from 4.94% to 21.75% (Table 3). Species delimitation analyses by ABGD and bPTP of the entire mitochondrial genomes always indicated seven species (Figure 2). We note that the interpretation of the bPTP result for *Bothriocroton dahlbergi* n. sp. is limited by the availability of only a single sequence. The multispecies coalescent analysis (Figure S4), executed with the seven hypothesised species as operational taxonomic units, recovered all seven lineages as monophyletic. However, the relationships among *B. auruginans, B. undatum,* and the *B. hydrosauri* group were unresolved, with low posterior support for this node (posterior probability = 0.52) (Figure S4).

A00 analyses of BPP produced highly consistent parameter estimates among independent runs, with terminal and internal population size parameters (θ) ranging from approximately 0.00068 to 0.244 and divergence time parameters (τ) ranging from 0.00065 to 0.014. These estimates were subsequently used to guide prior selection for the A11 species delimitation analyses. Among the six A11 analyses, the seven-species model was favoured in five of six prior combinations, with posterior probabilities ranging from 0.571 to 0.921 (Table 4). The only competing model with substantial support was a six-species scenario in which *Bothriocroton bulli* n. sp. and *B. hydrosauri* were merged. This model was preferred only under the most restrictive prior population size [θ ~ IG(3,0.002)] combined with τ ~ IG(3,0.05), where it had a posterior probability of 0.562. All of the other analyses recognised *B. bulli* n. sp. and *B. hydrosauri* as distinct species (PP = 0.571–0.921). No alternative species delimitations received appreciable support.

### 3.4. Systematics

Family Ixodidae Murray, 1877

Subfamily Bothriocrotoninae Klompen, Dobson & Barker, 2002

Genus *Bothriocroton* Keirans, King & Sharrad, 1994

***Bothriocroton bulli* n. sp. Barker, 2026**

Etymology: *B. bulli* n. sp. is named for the late Professor Michael Bull, who discovered the first evidence that the *Bothriocroton* ticks on *Tiliqua rugosa*, the sleepy lizard, in southwestern WA were substantially different to the *Bothriocroton* ticks on *T. rugosa* in southeastern Australia [43]. We are also pleased to have the opportunity to highlight the outstanding contribution that our good friend Michael Bull made to the understanding of the ecology of *B. hydrosauri* and *Amblyomma limbatum,* and other species of Australian ticks.

*Holotype:* Male; Pemberton, WA, Australia (−34.44, 116.04); altitude, 175 m; collectors, Stephen and Dayana Barker; Barker & Barker reference number (ref. #) **B4922**; 1 October 2015; collected from *T. rugosa*, the sleepy lizard; deposited in the Queensland Museum (QM S124260); NCBI GenBank Accession numbers PZ836786 (mt genome).

*Paratypes*: Three females and one male; Pemberton, WA, Australia (**B4922**, data as for holotype); deposited in the QM (S124261). One female, Boxwood Hill, Bremer Bay, WA, Australia (−34.36, 118.75); altitude, 75 m; collectors, Stephen and Dayana Barker; Barker & Barker ref. # **B4931**; October 2015; collected from *T. rugosa*; deposited in the QM (S124262); NCBI GenBank Accession numbers PZ836787 (mt genome), PZ835946 (nuclear rRNA). One female; Margaret River, WA, Australia (−33.95, 115.07); altitude, 90 m; collectors, Stephen and Dayana Barker; Barker & Barker ref. # **B4918**; 10 October 2015; collected from *T. rugosa*; deposited in the QM (S124263); GenBank Accession numbers PZ836785 (mt genome), PZ835945 (nuclear rRNA).

*Other material examined:* refer to Table 1.

*ZooBank:* The Life Science Identifier (LSID) for *Bothriocroton bulli* n. sp. Barker, S.C. is http://zoobank.org/urn:lsid:zoobank.org:act:12A48F1E-DB7F-4529-BC73-2C0406FE4BD7.

**Description**

**Figure 5:**
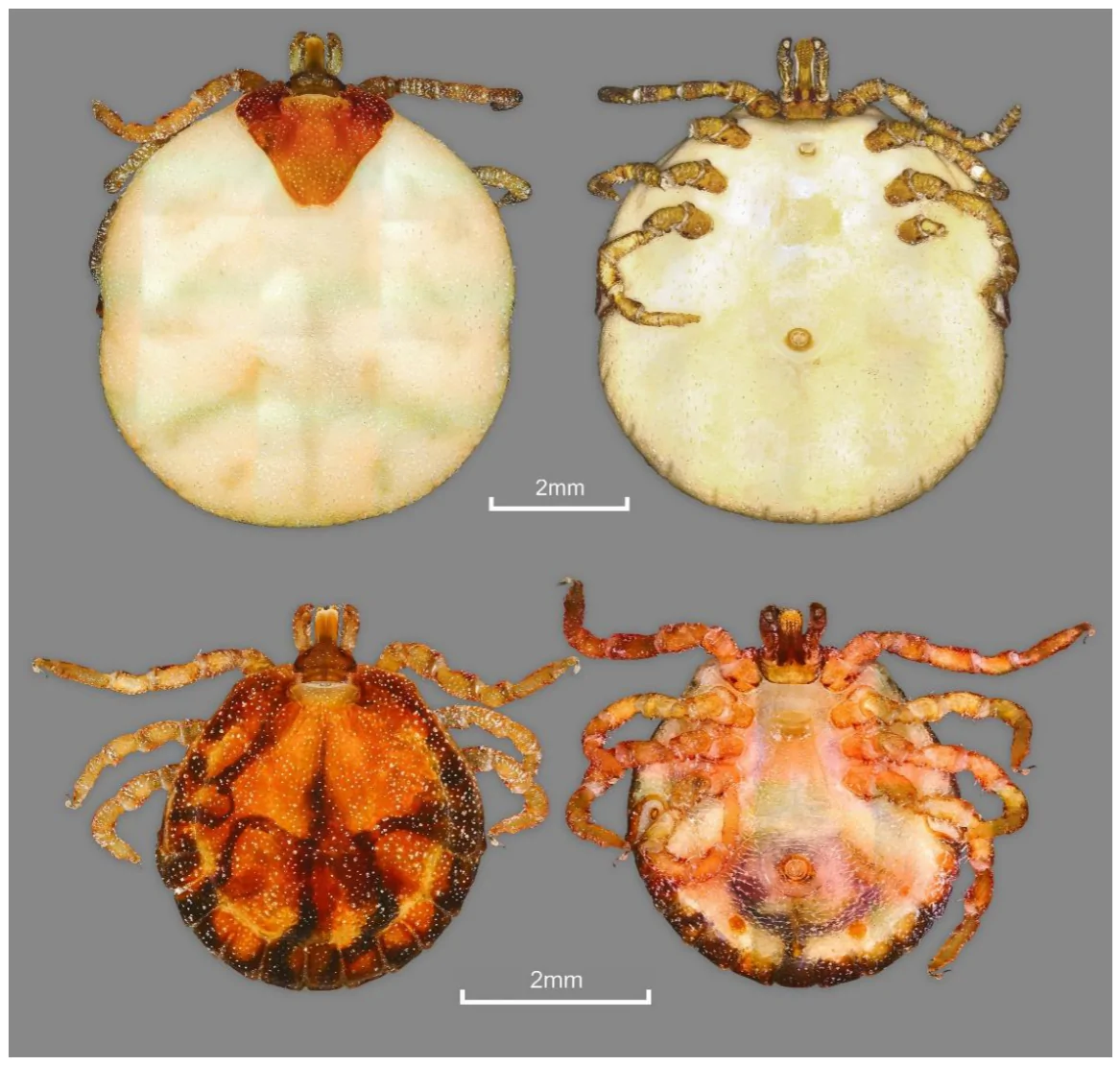
*Bothriocroton bulli* n. sp. light photograph. (**Top**) female (paratype); (**Bottom**) male (holotype), B4922 (Pemberton, WA, Australia).

**Female** (Figure 5). **Gnathosoma** (Figures S5–S7). Mouthparts narrow, slender and elongate. Hypostome (dorsal view) slightly shorter than palps. Length 0.56 mm (n = 1). Small to no dorsal spur on palpal article 2. Palpal article 3 rounded to squircular (i.e., a shape intermediate between a square and a circle). Setae present, particularly medially. Palpal article 1 poorly distinguished from palpal article 2; 3/3 stout dental formula, followed by additional minute files of five to six rows of denticles diagonally arranged. Basis capitulum (ventral view) mostly smooth. Length 0.28 mm, width 0.73 mm (n = 1). Porose areas large, subcircular to pyriform, pointed medially. Basis capitulum margin with slightly undulating, slight double concavity, forming medial indent (notch). Small projection on cervix (dorsal view).

**Scutum** (Figures S8 and S9). Cordiform, single shallow concavity on lateral margin. Length 1.63 mm, width 2.28 mm (n = 1). No apical indent, no enamel present on the specimens examined. Scutum is punctate: punctations small on medial aspect and intercervical area, but larger on lateral aspect and scapulae. Large punctation confluences forming lines of depressions on lateral aspect. Small setae sparse on the scapulae and in lateral groove, otherwise scutum bare. Scapulae pointed apically, margin slightly concave to angle. Intercervical area trapezoid shape, with ‘wings’ at anterolateral angle. Cervical grooves wide, with primary groove not extending the entire length of the lateral intercervical area. Additional distinct narrow groove running closely alongside lateral intercervical wall.

**Alloscutum** (Figure S10). Circular, covered in setae in a bilateral symmetrical pattern. Posterolateral and medial grooves with few setae, extending to festoon margin. Idiosoma length 6.50 mm, width 6.00 mm (n = 1) when engorged. Unknown for unengorged.

**Venter and coxae** (Figures S11 and S12). Genital aperture between coxa 2 and 3. Genital grooves and anal suture shallow. Coxa 1 internal and external spurs small. Coxa 2–4 also with small external spurs, no internal spurs.

**Tarsi**. Single small dorsal hump on all tarsi.

**Male** (Figure 5). **Gnathosoma** (Figures S13 and S14). Mouthparts slender. Dorsal palpal article 2 with a small distal spur. Long setae on palpal margins and spinal ridge, including pairs of long setae on palpal article 1. Hypostome dentition 3/3 stout files, with occasional minute teeth, though not forming another file. Length 0.48 mm (n = 1). Basis capitulum punctate dorsally, subpentagonal with indented basis margin. Length 0.24 mm, width 0.65 mm (n = 1). Small, pinched posterolateral salience at basis angle. Cervix smooth dorsally, rugose laterally with small cervical projection. Ventral basis smooth, single patch of follicles located centrally.

**Conscutum** (Figures S15–S17). Circular. Length 3.46 mm, width 3.38 mm (n = 1). Scapulae not distinct, broad and evenly rounded, closely associated with the capitulum. Intercervical area trapezoidal, punctate with short, fine setae. Cervical grooves wide, short, with moderately shallow lateral angle. Conscutal surface overall evenly punctate, though punctations small. Punctation density greatest in lateral grooves, being distinct and continuous, though shallower than *B. hydrosauri*. Lateral groove expanded at the posterior extreme of conscutum, to form bilateral indentations on either side of the midline, punctations here larger and denser than on rest of the conscutum, comparable to *B. hydrosauri*. Eleven distinct festoons. Few setae, small and fine when present.

**Venter and coxae** (Figures S18 and S19). Striated, with fine setae. Genital hood between coxa 2 and coxa 3, small, fine teeth-like structures present on posterior margin of genital hood. Genital grooves distinct, continue past anus to internal festoon margin. Ventral plaques present anterior to internal festoon margin. Posterior anal groove deep, without lip, short anal suture to medial ventral plaque. Coxa 1 with internal and external spurs of similar size, close together. Coxa 2–4 with single small external spur, little change with progression.

**Tarsi**. Long and fine with small and blunt single dorsal hump midway down tarsus. Distinct pulvillus.

**Remarks and differential diagnosis**. Male *B. bulli* n. sp. can be distinguished from *B. hydrosauri* by reference to the lateral grooves. In *B. hydrosauri*, the lateral grooves are deep, composed of many large and sometimes confluent punctations that do not extend onto the scapulae. In *B. bulli* n. sp., the lateral grooves are shallower, composed of small punctations that extend onto the scapulae. In addition, *B. bulli* n. sp. males are typically evenly round ticks, with wide scapulae that are tightly associated with the capitulum, whereas *B. hydrosauri* males are more pyriform, being rounded posteriorly and slenderer anteriorly, with sloping scapulae that are more loosely associated with the capitulum. Also, female *B. bulli* n. sp. have wide cervical grooves whereas *B. hydrosauri* females have much narrower grooves. *B. bulli* is known only from southwestern WA whereas *B. hydrosauri* is only known from eastern Australia (southeastern SA, Vic, Tas, NSW, Qld).

The *B. hydrosauri* group (*B. hydrosauri*, *B. bulli* n. sp. and *B. dahlbergi* n. sp.) is readily distinguishable from other *Bothriocroton* and other ixodid ticks since females of the *B. hydrosauri* group are large, darkly-coloured and circular, with highly pilose dorsal alloscuta, and often with a conspicuous spot of enamel at the scutal apex. Males of the *B. hydrosauri* group are also darkly coloured, highly punctate, with deep lateral grooves, posterior conscutal indentation and ventral plaques. Refer also to the dichotomous keys to adult female and male *Bothriocroton* ticks (Figure 11).

Family Ixodidae Murray, 1877

Subfamily Bothriocrotoninae Klompen, Dobson & Barker, 2002

Genus *Bothriocroton* Keirans, King & Sharrad, 1994

***Bothriocroton concolor* (Neumann, 1899). Redescription.**

**Type depository:** Zoologischen Museums Berlin, Germany (holotype) (Moritz, M. and Fischer, S.C. 1981. Die Typen der Arachniden-Sammlung des Zoologischen Museums Berlin. Mitt. Zool. Mus. Berlin, 57: 341–364) as *Aponomma concolor* (refer to Guglielmone et al. 2014) [44,45,46]. Holotype apparently lost.

*Material examined:* refer to Table 1.

**Redescription**

**Figure 6:**
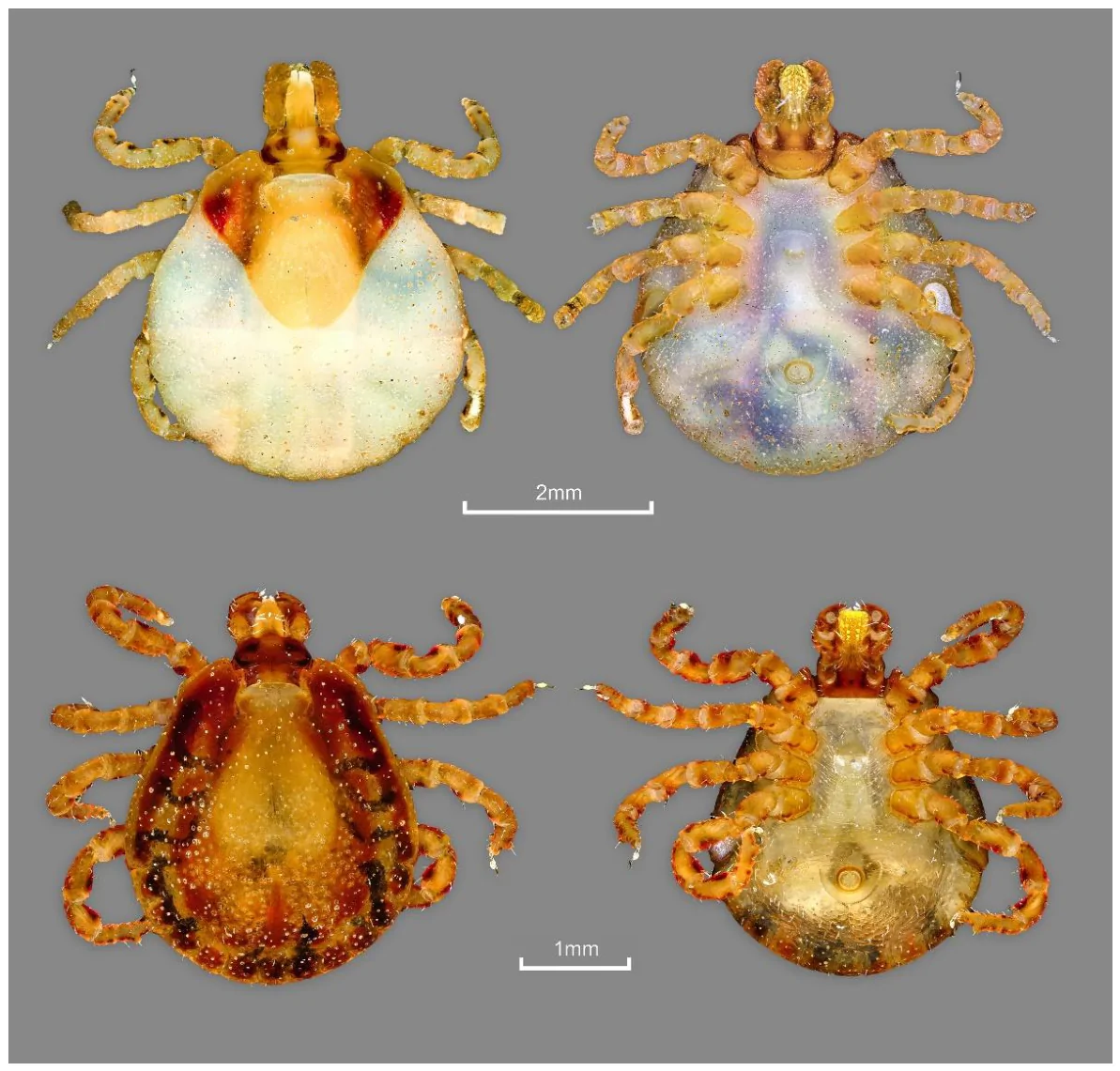
*Bothriocroton concolor* light photograph. (**Top**) Female, B3007 (Mt Ommaney, Qld); (**Bottom**) Male, B8060 (Australia Zoo Wildlife Hospital, Qld).

**Female** (Figure 6). **Gnathosoma** (Figures S5–S7). Mouthparts wide, dorsal hypostome slightly shorter and narrower than palps. Length 0.72 mm (n = 1). Robust medial distal salience on dorsal palpal article 2. Palpal article 3 subtriangular, rounded, with a wide base meeting distal palpal article 2. Long setae present on palpal margins, particularly medial dorsal and ventral. Ventral palps featuring obvious process on distomedial palpal article 2, which, as Roberts (1970) suggested, resembles a finger, crossing the medial palpal margin [5]. Palpal article 3 also with a distomedial spur, though less prominent. Palpal article 1 poorly distinguished from palpal article 2. Hypostome dentition 3/3 with two files of stout teeth and an innermost file of minute teeth. Basis capitulum length 0.32 mm (n = 1), width 0.89 mm (n = 1). Ventral basis capitulum apunctate, with smooth surface, slightly clefted laterally. Porose areas small, circular to laterally pyriform. Basis posterior margin weak, indented, slightly sinusoidal. Cervix unremarkable.

**Scutum** (Figures S8 and S9). Cordiform profile, with slightly indented apex and concave posterolateral margins. Length 1.94 mm, width 2.02 mm (n = 1). Medial aspect evenly punctate, lateral aspects more sparsely so, with size of punctations varying. Punctations on intercervical area small anteriorly becoming larger posteriorly. Substantial concavities at posterior of lateral aspects, extending to posterolateral scutal margin. Setae present here, and on scapulae, otherwise bare. Cervical grooves long, narrow, and distinct, corrugated throughout. Projection angle from pinch point shallow, cutting into intercervical wall gradually, forming long grooves and a large, subhexagonal intercervical area. Lateral aspects frequently darkly coloured—red, brown or amber—whereas the medial aspects and intercervical areas usually markedly paler in colour.

**Alloscutum** (Figure S10). Body profile subcircular overall. Short setae distributed evenly in distinct alloscutal punctations. Posterolateral grooves and medial suture distinct. Eleven festoons, distinct. Idiosoma length 3.50 mm, width 3.33 mm (n = 1) when unengorged.

**Venter and coxae** (Figures S11 and S12). Short, fine setae overall. Genital aperture between coxae 2 and 3. Genital grooves distinct, diverging at anus. Medial ventral suture projects from posterior anal groove to festoon margin. Large, blunt external spurs on all coxae. Internal spur on Coxa 1 of comparable size to external spur. Anterior process diminishes in salience down coxae.

**Tarsi**. Tarsus with single salient dorsal hump, tarsus 1–3 without.

**Male** (Figure 6). **Gnathosoma** (Figures S13 and S14). Hypostome length 0.46 mm (n = 1). Mouthparts as in female—relatively stubby, and broad, though not extending laterally beyond lateral capitulum margin. Dorsal basis capitulum lightly punctate, basis margin with single concavity. Length 0.26 mm, width 0.68 mm (n = 1). No cornua, but some salience at angles.

**Conscutum** (Figures S15–S17). Body profile elliptical. Length 2.93 mm, width 2.61 mm (n = 1). Punctate overall, becoming particularly large and dense posteriorly. Small medial and posterolateral regions without punctation.

**Scapulae** loosely associated with capitulum. Intercervical area subhexagonal, with small, even punctation. Cervical grooves as in female. Lateral groove depressed shallowly, with large punctations. Setae sparse in lateral groove and punctate posterior conscutum. Eleven distinct festoons.

**Venter and coxae** (Figures S18 and S19). Evenly distributed, small, fine setae. Genital hood between coxa 2 and coxa 3, small, fine teeth like structures present on posterior margin of genital hood. Genital grooves terminate at anus. Ventral plaques absent. Posterior anal groove without lip, short anal suture to festoon margin. Coxae as in female, although spurs further apart on coxa 1.

**Tarsi**. Tarsi as in female.

**Remarks and differential diagnosis**. *B. concolor* is one of nine species that parasitises echidnas in Australasia, four of which are *Bothriocroton*. The *B. concolor* group (*B. concolor*, *B. spratti* n. sp.) can be distinguished from the other species of *Bothriocroton* by reference to the following characteristics. Females of the *B. concolor* group have small, circular porose areas, and narrow, deep, corrugated cervical grooves; the scutal posterolateral margins are concave, and the scuta are often characterised by darkly coloured lateral aspects, contrasting a pale medial aspect.

Males of the *B. concolor* group have a highly punctate conscutum, particularly on the posterior aspect, where the punctations become large and dense. Both sexes have wide palps that are markedly broader, proportionally, than in other species, and both with a characteristic ‘finger-like’ process on ventral palpal article 2 projecting past the medial margin.

Females of *B. hydrosauri* have larger porose areas, and males have ventral plaques, which in addition to the distinguishing features of the *B. concolor* group (above), distinguish these ticks.

The two ticks from PNG that we genotyped clustered with the *B. concolor* lineage (Figure 2, B0895). These two ticks were subsequently lost, however, so further investigation is required to confirm whether *B. concolor* is indeed present in PNG. Unfortunately, records of PNG *B. concolor*, including Hoogstraal (1982), conflate *B. concolor* and *B. oudemansi* [47]. Along with *B. oudemansi, B. concolor* is the second echidna *Bothriocroton* possibly known from PNG. Although *B. oudemansi* apparently primarily parasitises *Zaglossus* echidnas, there is a report in Beati et al. (2008) of *B. oudemansi* on a *T. aculeatus* as well [16]. *B. oudemansi* and *B. concolor* are easily distinguished since *B. oudemansi* has extruding spiracle plates and slender palps, in contrast to *B. concolor*. *B. hydrosauri* is also found on echidnas in Qld.

Distinguishing *B. concolor* and *B. spratti* n. sp. by their morphology is challenging. But in both males and females, the cervical grooves of *B. concolor* have a shallow angle relative to the pinchpoint, curving to form the ‘neck’ of the intercervical area more gradually, resulting in a longer overall groove. In contrast the cervical grooves of *B. spratti* n. sp. are angled more sharply after the pinch point, resulting in a shorter groove, and a more elliptical intercervical area.

The type locality is “Queensland”. Thus, we chose to use the name *Bothriocroton concolor* (Neumann, 1899) for the clade that contains ticks from Qld (Figure 2, Figure 3 and Figure 4).

Family Ixodidae Murray, 1877

Subfamily Bothriocrotoninae Klompen, Dobson & Barker, 2002

Genus *Bothriocroton* Keirans, King & Sharrad, 1994

***Bothriocroton dahlbergi* n. sp. Barker, 2026**

Etymology: *B. dahlbergi* n. sp. is named for Amber Dahlberg (Wildlife & Raptor Care Qld; WARC), who collected the holotype and paratypes.

*Holotype:* Female; Mt Molloy, Qld, Australia (−16.70, 145.33); altitude, 360 m; collector, Amber Dahlberg; Barker & Barker ref. # **B7139**; 19 January 2023; collected from *Tachyglossus aculeatus*, the short-beaked echidna; deposited in the QM (S124264); NCBI GenBank Accession numbers PZ836794 (mt genome), PZ835952 (nuclear rRNA).

*Paratypes:* Four females; (**B7139**, data as for holotype). Deposited in the QM (S124265).

*Other material examined* (*Barker & Barker Collection*), *provisionally designated as Bothriocroton dahlbergi* n. sp.: 1 male; Captain Billy Ck, Cape York Peninsula, FNQ (−11.66, 142.85); altitude, 8 m; collector unknown; Barker & Barker ref. # B3148; 9 July 1975; collected from *T. aculeatus*.

*ZooBank:* The Life Science Identifier (LSID) for *Bothriocroton dahlbergi* n. sp. Barker, S.C. is http://zoobank.org/urn:lsid:zoobank.org:act:8EBE20F4-B378-44D3-B8B5-C226356E7CD0.

**Description**

**Figure 7:**
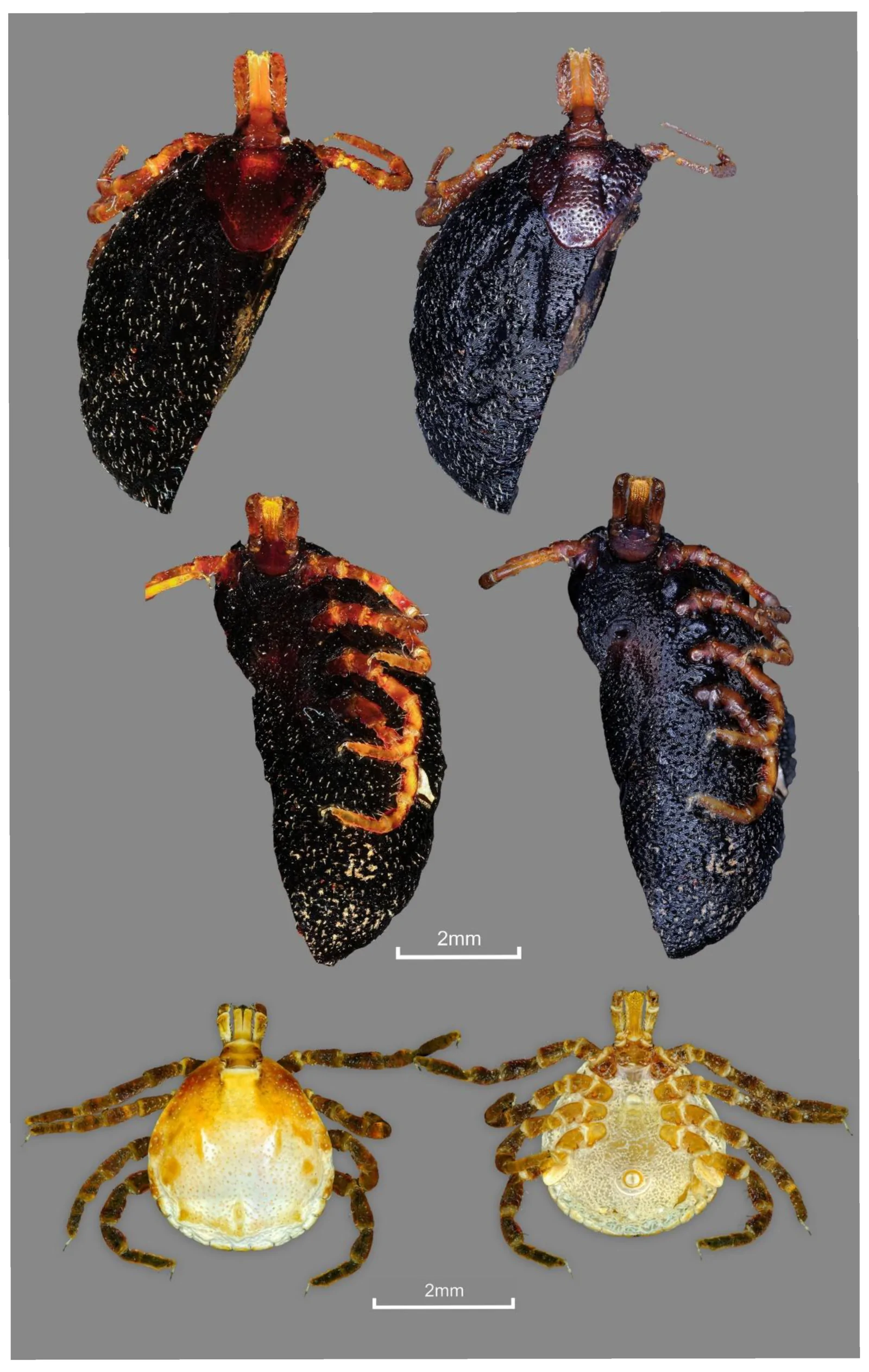
*Bothriocroton dahlbergi* n. sp. light photograph. (**Top**) Female **B7139** from Mt Molloy, Far North Qld (FNQ); (**Bottom**) Male **B3148** from Captain Billy Ck, FNQ.

**Female** (Figure 7, top). **Gnathosoma** (Figures S5–S7). Long, slender mouthparts, with hypostome and palps of equal length. Little to no distal dorsal spur on palpal article 2. No ventral palpal article 2 spur. Palpal margins well within lateral basis axis. Long setae, relatively thick, present on palpal margins, particularly medial margin. Palpal article 3 is rounded, subtriangular. Hypostome length 0.89 mm (n = 1), dentition 4/4, with three files of six or seven strong teeth decreasing in size posteriorly and internally, plus a fourth innermost file of minute teeth. Trapezoidal basis profile, with squared anterior aspect. Porose areas are moderately sized, lachrymiform. Philtrum present between porose areas. Basis margin robust, undulating, with three shallow concavities. Length 0.33 mm, width 0.87 mm (n = 1). Cervix is weakly rugose, without obvious projection.

**Scutum** (Figures S8 and S9). Diamond-shaped, with wide lateral aspects. Length 1.84 mm, width 1.94 mm (n = 1). Scapulae pointed, closely associated with capitulum. Scapular margin shallowly rounded, anterolaterally to angle. Evenly punctate overall, with little confluences in lateral aspect. Sparse, fine, short setae overall. Intercervical area rectangular. Cervical grooves shallow, uncorrugated. Lateral carinae present. No apical enamel present.

**Alloscutum** (Figure S10). Not present intact from available specimen. Idiosoma length 6.22 mm (n = 1) when engorged.

**Venter and coxae** (Figures S11 and S12). Coxa with minute striations on the surface (only visible with SEM), with thin setae evenly distributed. Coxa 1 with internal and external spurs of comparable size, widely separated. Coxa 2–4 without internal spur, external spur remaining salient. Anterior process reduces in salience from coxa 1–4.

**Tarsus**. Single dorsal hump of little salience on all tarsi.

**Male** (Figure 7, bottom). **Gnathosoma** (Figures S13 and S14). Mouthparts as in female, although dental files are more diagonal. Hypostome length 0.50 mm (n = 1). No distal ventral margin on palpal article 2. Basis capitulum with many small dorsal punctations, overall shape is trapezoidal. Length 0.28 mm, width 0.56 mm (n = 1). Posterolateral basis salience at an angle. Basis margin is strongly concave, rounded. Cervix without dorsal projection.

**Conscutum** (Figures S15–S17). Body profile pyriform. Length 3.08 mm, width 2.60 mm (n = 1). Scapulae narrow, tapers quickly. Intercervical area rectangular, with small punctations and sparse setation. Cervical grooves narrow and shallow. Conscutal surface highly punctate—uneven distribution across central dorsum, highly dense around shallowly depressed continuous lateral groove. Posterior cuticle indentations expand the lateral groove bilaterally at the anterior festoon margin, composed of many large, dense punctations. Extremely sparse setation in lateral groove. Eleven festoons, distinct.

**Venter and coxae** (Figures S18 and S19). Genital hood at level of coxa 2. Ventral plaques present. Coxae as in female, although internal coxa 1 spur slightly more slender.

**Tarsus.** As in female.

**Remarks and differential diagnosis**. *B. dahlbergi* n. sp. can be distinguished from the other species of *Bothriocroton* by reference to scutal punctation. All female *B. hydrosauri* examined had distinct depressed bilateral lines of deep, large and confluencing punctations in each of the lateral scutal aspects, mirroring the top of the male lateral groove. On the other hand, *B. dahlbergi* n. sp. had punctations throughout the scutal surface, but without the depressed ‘grooves’ and with little to no confluencing punctation. Moreover, the cervical grooves of the *B. dahlbergi* n. sp. were markedly shallow, wrinkled, with an uneven medial margin, as compared to Qld *B. hydrosauri*, the margins of which are much clearer, with deeper grooves.

We found differences between the Captain Billy Creek male, which was provisionally designated as *Bothriocroton dahlbergi* n. sp., and males of *B. hydrosauri*; notably, the absence of any distal ventral salience on palpal article 2, and the uniquely rounded concavity of the basis margin. However, we are cautious in ascribing any weight to these characters due to the age and deterioration of the specimens from Captain Billy Creek, and the variable nature of these characters in *B. hydrosauri.* The females of *B. dahlbergi* n. sp. (together with the Captain Billy Creek male) had a consistent 4/4 stout dental formula, sometimes with an additional fifth internal minute file. *B. hydrosauri,* most commonly had a dental formula of 3/3 stout, with an additional fourth internal minute file. However, some *B. hydrosauri* specimens have an enlarged internal minute file, or a stout fourth file, and so this character is not diagnostic. These limited differential morphological characters contrast with the high degree of genetic distinction: approximately 18%, between *B. dahlbergi* n. sp. and *B. hydrosauri*. *B. dahlbergi* n. sp. is known only from Mt Molloy, on the Atherton Tablelands in Far North Qld and possibly Cape York Peninsula, Qld, whereas the most northerly known *B. hydrosauri* are from East End via Calliope, near Gladstone, Qld (B8394).

Family Ixodidae Murray, 1877

Subfamily Bothriocrotoninae Klompen, Dobson & Barker, 2002

Genus *Bothriocroton* Keirans, King & Sharrad, 1994

***Bothriocroton hydrosauri* (Denny, 1843). Redescription.**

**Type depository**: Not stated in Denny, H. (1843) [48]. Description of six supposed new species of parasites. Ann. Mag. Nat. Hist., 12: 312–316) as *Ixodes hydrosauri.* Types presumed lost.

*Material examined:* refer to Table 1.

**Redescription**

**Figure 8:**
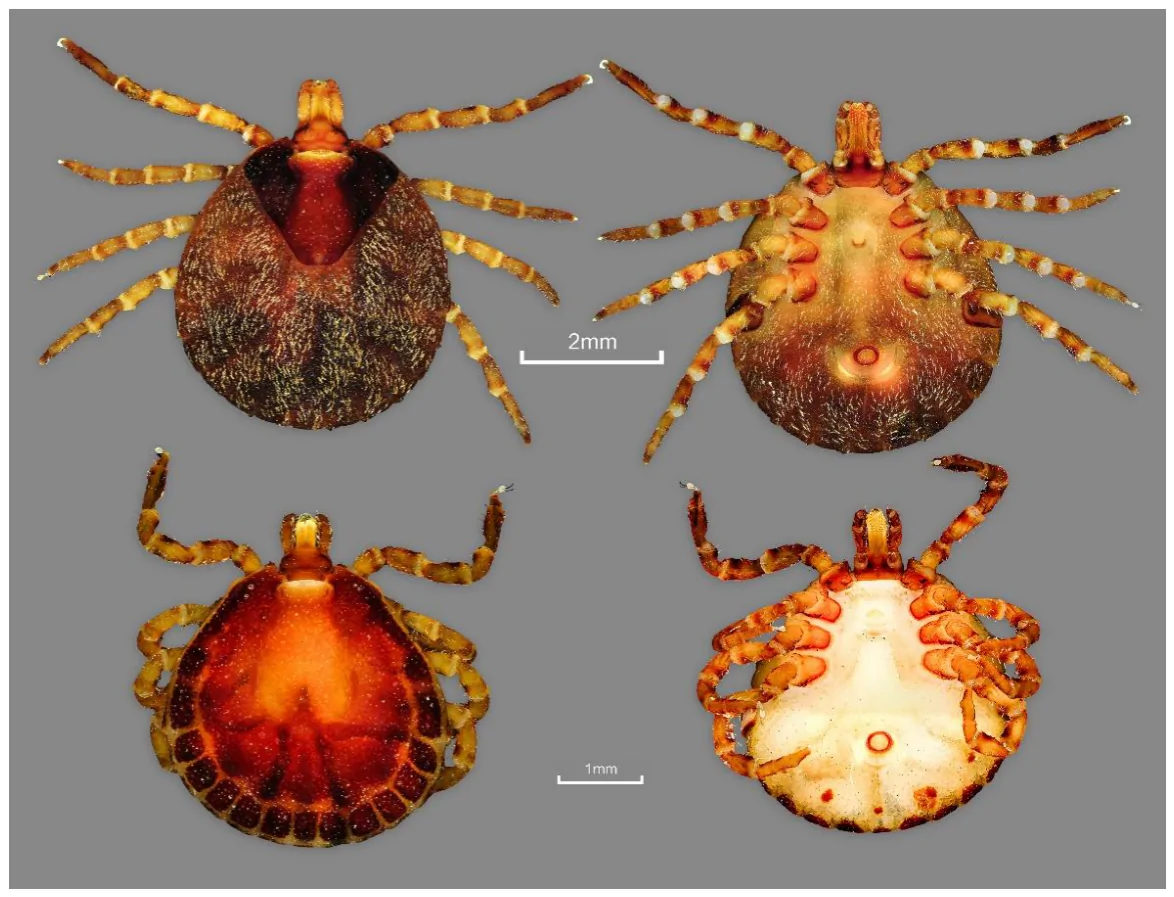
*Bothriocroton hydrosauri* light photographs. (**Top**) Female, B8037 from Leongatha, Vic; (**Bottom**) male, B8473 from East Coast Veterinary Clinic, St Helens, Tas.

**Figure 9:**
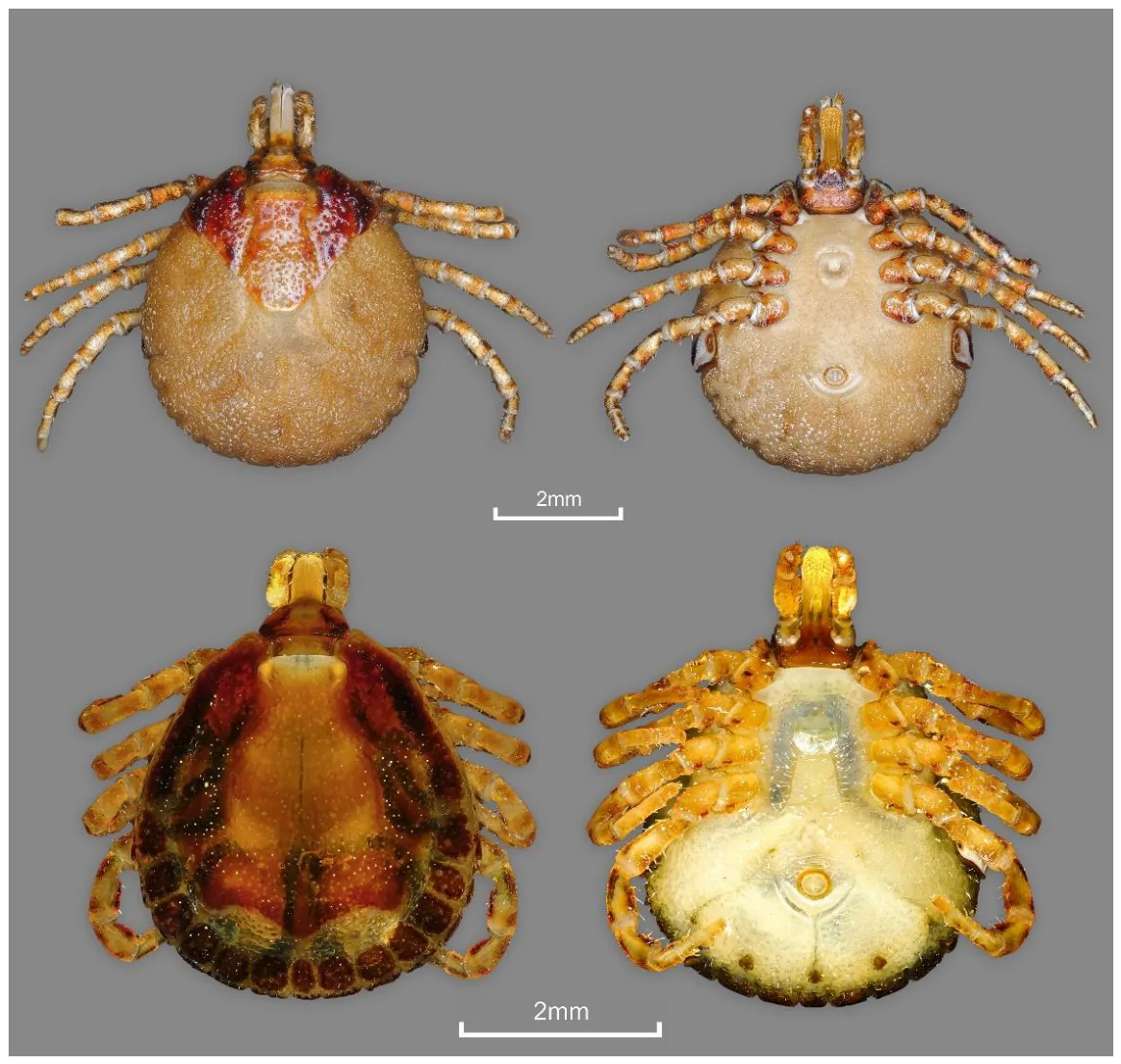
*Bothriocroton hydrosauri* (Qld specimens) light photograph. (**Top**) female and (**Bottom**) male from B8395 (Damascus via Gin Gin, Qld).

**Female** (Figure 8 and Figure 9, top). **Gnathosoma** (Figures S5–S7). Mouthparts narrow, slender and elongate. Dorsal hypostome longer than palps. Palps without medial spurs, with ridge following the spine of palpal article 2, palpal article 3 broad and rounded, palpal article 1 with two sets of paired setae, medially and laterally, long setae particularly prominent on other palpal margins and ridgeline, internal palpal face concave, punctated with undeveloped setal follicles. Hypostome length 0.60 mm (n = 1), dentition 4/4, with three files of about eight strong teeth, plus a fourth innermost file of minute teeth. Basis capitulum subpentagonal, posterior basis margin sinusoidal, bilateral double concavity forming medial indent or notch. Length 0.23 mm, width 0.76 mm (n = 1). Slight, blunt posterior salience at posterolateral angle. Large elliptical to lachrymiform porose areas, highly porose webbing, and broad, uneven depressed philtrum between porose areas. Lateral aspect and posterior seam of cervix is rugose, dorsal aspect with prominent, wide cervical projection.

**Scutum** (Figures S8 and S9). Scutum cordiform. Length 1.83 mm, width 2.14 mm (n = 1). Scutal surface evenly punctate, otherwise smooth, except for lateral aspect where there is a confluence of punctations form uneven depressions. Short, fine setae sparsely distributed across scutum, although more dense on the intercervical area. Scapulae with pointed apex, tightly wrapped with capitulum, slight concavity in margin before rounding out anterolaterally. Intercervical area elliptical, with rugose anterior margin. Cervical grooves relatively smooth, without corrugation or setation. Cervical groove lateral angle shallow.

**Alloscutum** (Figure S10). Circular. Highly pilose, with long, thick setae arranged with indefinable bilateral symmetry. Posterolateral grooves and medial suture bare. Paired false ocelli present immediately posterior to scutal apex and anterior to medial suture. Eleven festoons, distinct. Idiosoma length 4.04 mm (n = 1) when not engorged.

**Venter and coxae** (Figures S11 and S12). Coxa with minute striations on the surface (only visible with SEM), with comparably thick setae as on alloscutum. Setae begin at level of coxa 2. Genital aperture between coxa 2 and coxa 3. Genital grooves slight, not continuing past anus. Anal groove posterior, thick-lipped. Anal suture continues to internal festoon margin. Coxa 1 with broad, blunted internal spur and smaller external spur. Coxa 2–4 without internal spur, external spur remaining salient, although small.

**Male** (Figure 8 and Figure 9, bottom). **Gnathosoma** (Figures S13 and S14). Mouthparts slender. Dorsal hypostome shorter than palps. Palps as in female, with tiny spur-like process ventrally on palpal article 2, though not projecting past medial palpal margin. Hypostome length 0.59 mm (n = 1), dentition 4/4, with three files of five or six strong teeth decreasing in size posteriorly and internally, plus a fourth and innermost file of minute teeth irregular in disposition and number and sometimes as few as 1 or 2. Basis capitulum subpentagonal, dorsal capitulum punctate, basis margin straight or slightly concave. Length 0.22 mm, width 0.61 mm (n = 1). Little to no cornual salience. Slight dorsal projection on the cervix (Figure S7), cervix mostly smooth.

**Conscutum** (Figures S15–S17). Subcircular. Length 3.30 mm, width 3.29 mm (n = 1). Scapulae blunt, loosely associated with capitulum, with rounded margins. Intercervical area with an anterior projection, dense, even punctation and sparse setae. Cervical grooves relatively smooth, with shallow lateral angle. Conscutal surface overall evenly punctate, though punctations small cf. *concolor*. Punctation density high in lateral grooves, which are deep, distinct and continuous. Groove expands at posterior extreme of conscutum, forming bilateral ‘indentations’ on either side of the midline, punctations here larger and denser than on remainder of conscutum. Eleven distinct festoons. Little setation, small and fine where present.

**Venter and coxae** (Figures S18 and S19). Striated, with small, fine setae. Genital hood between coxa 2 and coxa 3, small, fine teeth-like structures present on posterior margin of genital hood. Genital grooves continue past anus to internal festoon margin. Ventral plaques present anterior to internal festoon margin. Posterior anal groove without lip, short anal suture to medial ventral plaque. Coxa 1 with internal and external spurs, internal marginally larger. Coxa 2–4 with single small external spur, little change with progression.

**Tarsi**. Small and blunt single dorsal hump midway down tarsus.

**Remarks and differential diagnosis**. The *B. hydrosauri* group (*B. hydrosauri*, *B. bulli* n. sp., *B. dahlbergi* n. sp.) is readily distinguishable from the other *Bothriocroton* species: females are larger, darkly coloured and circular, commonly with pilose dorsal alloscuta, and often with a conspicuous spot of enamel at the scutal apex. Males of the *B. hydrosauri* group are also darkly coloured, highly punctate, with deep lateral grooves, posterior conscutal indentation and ventral plaques. There is much variation in morphology among populations of *B. hydrosauri*, which was first recognised by Andrews et al. (2006) [17]. *B. hydrosauri* females from Qld may have smaller, more lachrymiform porose areas, compared to southern counterparts. Andrews et al. (2006) highlighted the indentations in the posterior conscutum cuticle in male ticks from Central Qld (which they referred to as *B. tachyglossi*) as a diagnostic factor. Whereas these indentations may indeed be more pronounced in ticks from Qld, indentations of varying salience can still be found in *B. hydrosauri* from southern populations as well [17].

Morphologically distinguishing *B. hydrosauri* from *B. bulli* n. sp. and *B. dahlbergi* n. sp. is challenging. But male *B. hydrosauri* can be distinguished from *B. bulli* n. sp. by reference to the lateral grooves. In *B. hydrosauri*, the lateral groove is deep, composed of many large or confluencing punctations, and does not extend onto scapulae. In *B. bulli* n. sp., the lateral groove is shallower, composed of small punctations, and extends onto the scapulae. In addition, *B. bulli* n. sp. males are typically evenly rounded ticks, with wide scapulae that are tight with the capitulum, whereas *B. hydrosauri* males are more pyriform, being rounded posteriorly and more slender anteriorly, with sloping scapulae that are more loosely associated with the capitulum. Female *B. bulli* n. sp. have wide cervical grooves whereas *B. hydrosauri* females have much narrower cervical grooves. Refer also to the dichotomous key to adult female and male *Bothriocroton* species (Figure 11).

All female *B. hydrosauri* examined had distinct lines of deep, large and confluencing punctations in each of the lateral scutal aspects, mirroring the top of the male lateral groove. *B. dahlbergi* n. sp. specimens had small punctations throughout the scutal surface, without the depressed ‘grooves’ and with little to no confluencing punctation. *B. hydrosauri* is conventionally understood to be a generalist reptile tick, particularly of *Tiliqua* and *Varanus* species [12]. The two new records of *B. hydrosauri* in Qld, however, were from short-beaked echidnas: East End via Calliope, B8394; Damascus via Gin Gin, B8395. What is more, there is no indication that *B. hydrosauri* in Qld parasitises reptiles, nor is there evidence that *B. hydrosauri* in southern Australia parasitise echidnas (Barker & Barker, unpublished data). *B. tachyglossi* was described in 1953 for female ticks from short-beaked echidnas (*T. aculeatus*) and cattle (*Bos taurus*) in central and south-eastern Qld (Wowan, Degilbo, Monto) [4]. Later, after seeing male ticks for the first time, Roberts (1970) synonymised *B. tachyglossi* and *B. hydrosauri* [5]. Our findings from mitochondrial genomes and nuclear rRNA genes concur with Roberts [5] in that the *Bothriocroton* ticks from central Qld from short-beaked echidnas (East End via Calliope, B8394; Damascus via Gin Gin, B8395) are *B. hydrosauri* (Denny, 1843). These findings contrast with those of Andrews et al. (2006), who re-examined the type specimens of Roberts (1953, 1970) and re-recognised and redescribed *B. tachyglossi* (Roberts, 1953) [4,5,17]. Andrews et al. (2006) emphasised the “large and highly punctate areas between the festoons and the scutum” in the male, as a diagnostic character, which they described as “very small or absent in specimens of *A. hydrosauri*” [17] [We also saw these features when we examined the holotype and paratypes of *B. tachyglossi* (Roberts, 1953)]. When we examined *B. hydrosauri* we also saw this dichotomy, but concluded that it is likely the dichotomy was overstated by Andrews [17]. The indentations in the posterior conscutum cuticle of the male, which take the form of an expansion of the lateral groove into the posterior cuticle bilaterally at the anterior festoon margin, are typically more pronounced in Qld *B. hydrosauri* than in the *B. hydrosauri* from southern Australia that we examined. Indeed, many southern *B. hydrosauri* males have comparably large indentations. That character notwithstanding, we observed the same tendency toward subtriangular porose areas in the Qld specimens, compared to the more circular characterisation of southern ticks, as did Roberts (1953) [4]. Qld specimens often also have pronounced distomedial expansions of the dorsal palpal article 2.

According to our findings from mitochondrial genomes and nuclear rRNA, however, the differences in morphology discussed above between *B. hydrosauri* (Denny, 1843) and *B. tachyglossi* (Roberts, 1953) [17] are not species-level differences. Andrews [17] also had some molecular data derived from allozyme (protein) gel electrophoresis, which provided evidence of genetic differences at 16% of the 18 allozyme gene loci they examined, between *B. hydrosauri* (Denny, 1843) and *B. tachyglossi* (Roberts, 1953) [17]. The rule of thumb at the time was that “populations of the same species seldom differ at more than 10% of loci, and almost never at more than 15%” [49]. A result of 16% difference thus supported, at the time, the species-level distinction of *B. hydrosauri* (Denny, 1843) and *B. tachyglossi* (Roberts, 1953) [17], but this conflicts with our mitochondrial genome matrices (Table 2 and Table 3). Our taxonomic conclusions, drawn from mitochondrial genome and nuclear rRNA genes, also conflict with the conclusions of Andrews [17] concerning their allozyme comparison of *B. hydrosauri* from eastern Australia and *B. hydrosauri* from WA (the *B. hydrosauri* from WA are described in the present paper as *B. bulli* n. sp.). Andrews [17] concluded that the allozyme data of Bull [50] indicated ca. 5% fixed gene (allozymes) differences between *B. hydrosauri* from eastern Australia and *B. hydrosauri* from WA, which was below the 10% threshold proposed for species-level differentiation [17,49,50]. On the other hand, we found percentage differences between the mitochondrial genomes of those from eastern Australia and *B. hydrosauri* from WA typical of different species of ticks—4.79–5.11% different (Table 3). Unless subsequent collections of *Bothriocroton* ticks from Qld reveal species-level differences when compared to *B. hydrosauri* (Denny, 1843) from southern Australia, *B. tachyglossi* (Roberts, 1953) should be, at some time in the future, synonymised once again with *B. hydrosauri* (Denny, 1843). We note with interest that the *B. hydrosauri* on echidnas in Qld live outside of the climate-profile that was described for *B. hydrosauri* in southern Australia [48,51].

The type locality of *Bothriocroton hydrosauri* (Denny, 1843) is “Van Diemen’s Land”, which is now known as Tasmania, Australia. Thus, we chose to use the name *Bothriocroton hydrosauri* (Denny, 1843) for the clade that contains ticks from Tasmania (Figure 2, Figure 3 and Figure 4). *B. hydrosauri* is widespread on mainland Australia as well as occurring in Tasmania (Table 1, Figure 2, Figure 3 and Figure 4).

Family Ixodidae Murray, 1877

Subfamily Bothriocrotoninae Klompen, Dobson & Barker, 2002

Genus *Bothriocroton* Keirans, King & Sharrad, 1994

***Bothriocroton spratti* n. sp. Barker, 2026**

Etymology: *B. spratti* n. sp. is named for Dr David M. Spratt, who contributed much to knowledge of Australian ticks, including *Bothriocroton* species.

*Holotype:* Male; Woodend, Vic, Australia (−37.35, 144.52); altitude, 570 m; collectors, James, Dyer, Luxmore, Robson and colleagues; Barker & Barker ref. # **B5461**; 26 August 2019; collected from *Tachyglossus aculeatus*, the short-beaked echidna; deposited in the QM (S124266). NCBI GenBank Accession number PZ836803 (mt genome).

*Paratypes:* Two males; Woodend, Vic, Australia (**B5461**, data as for holotype); deposited in the QM (S124267).

One male and one female; Phillip Is., Vic, Australia (−38.48, 145.26); altitude, 40 m; collectors, Farrer, Tuttle and colleagues; Barker & Barker ref. # **B8141**; September 2023; collected from *T. aculeatus*; deposited in the QM (S124268); NCBI GenBank Accession numbers PZ836808 (mt genome) and PZ835960 (nuclear rRNA).

*Other material examined:* Refer to Table 1.

*ZooBank:* The Life Science Identifier (LSID) for *Bothriocroton spratti* n. sp. Barker, S.C. is http://zoobank.org/urn:lsid:zoobank.org:act:CEA39053-C0ED-4920-AEB3-19126EA07C09.

**Description**

**Figure 10:**
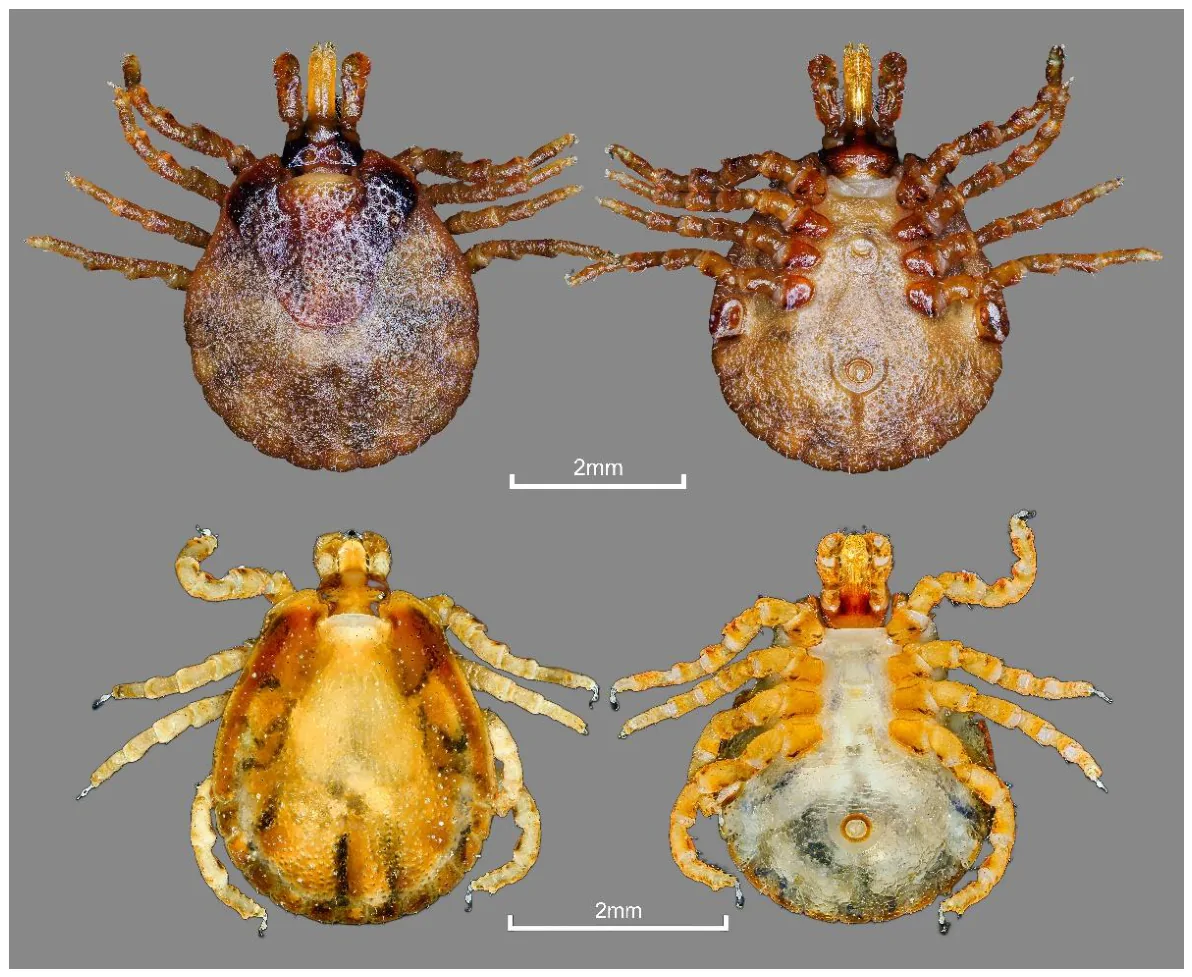
Light photographs of *Bothriocroton spratti* n. sp. (**Top**) Female, B5485 from Kangaroo Island, SA; (**Bottom**) male, **B8141** from Phillip Island, Vic.

**Female** (Figure 10, top). **Gnathosoma** (Figures S5–S7). Mouthparts broad, though remaining within basis lateral margin. Hypostome (dorsal view) shorter than palps. Length 0.87 mm (n = 1). Palps dorsally wide, each comparable to width of dorsal hypostome. Dorsally, robust distal medial spur on palpal article 2. Palpal article 3 with a wide base to meet distal palpal article 2, narrows distally forming subtriangular overall shape. Long setae on palpal margins, particularly dorsomedial margin of palpal article 3, medial margin of palpal article 2; and dorsal proximal margin of palpal article 2. Ventral palps with obvious process on distomedial palpal article 2, which, as Roberts [5] suggested, resembles a finger, particularly a pronated, cocked thumb. Palpal article 3 also with a distomedial spur, though less prominent than the spur on palpal article 2. Palpal article 1 poorly distinguished from palpal article 2. Hypostome dentition 3/3 with 2 files of stout teeth and an innermost file of minute teeth. Basis capitulum apunctate. Length 0.33 mm, width 0.85 mm (n = 1). Porose areas small proportional to basis. No philtrum between porose areas. Basis posterior margin straight, slight posterior rounded salience at angle. Cervix unremarkable; smooth, without rugation or dorsal projection.

**Scutum** (Figures S8 and S9). Cordiform, with indented apex, concave posterolateral margin. Length 1.97 mm, width 2.14 mm (n = 1). Median aspect evenly punctate, lateral aspects sparsely punctate. Large concavities in lateral aspects form from confluence of large punctations, extending to posterior scutal margin. Short, fine setae present on lateral and distal aspects. Scapulae pointed, loosely associated with capitulum. Slight concavity in scapular margin before rounding out anterolaterally. Intercervical area elliptical, laterally elongate, squat. Cervical grooves short and deep, corrugated. Cervical groove angle sharp from pinch point, with medial wall punctation.

**Alloscutum** (Figure S10). Body profile circular. Lightly pilose, with thin setae emerging from wide punctations. Posterolateral grooves and medial suture present. Eleven festoons distinct. Idiosoma length 3.56 mm (n = 1) when unengorged.

**Venter and coxae** (Figures S11 and S12). Thin setae evenly distributed. Setae begin at level of coxa 2. Genital aperture between coxa 2 and coxa 3. Genital grooves slight, angle diverging laterally at anus. Anal groove posterior, groove clefted. Anal suture continues to internal festoon margin. Coxa 1 with internal and external spurs of comparable sizes. Coxa 2 with slight impression of internal spur, coxa 3–4 without internal spur, external spur remaining salient. Anterior process reduces in salience from coxa 1 to coxa 4.

**Tarsi**. Tarsus 4 with single salient dorsal hump, tarsus 1–3 without.

**Male** (Figure 10, bottom). **Gnathosoma** (Figures S13 and S14). Mouthparts as in female, although ventral processes on palpal article 2 and palpal article 3 appear sharper. Hypostome length 0.42 mm (n = 1). Basis capitulum with few small dorsal punctations. Length 0.22 mm, width 0.64 mm (n = 1). Overall shape trapezoidal. Posterolateral salience, basis margin otherwise relatively straight. Cervix unremarkable.

**Conscutum** (Figures S15–S17). Body elliptical. Length 3.06 mm, width 2.57 mm (n = 1). Scapulae blunt, with rounded margins. Intercervical area elliptical, with anterior projection. Intercervical area with uneven, large punctations. Intercervical area setation fine and sparse. Cervical grooves as in female—corrugated, with sharp inward angle and punctation on medial wall of groove. Conscutal surface overall punctate—anterior aspect evenly so, with consistent size. Posterior aspect increases punctation salience, becoming significantly larger and more dense. Small median and posterolateral regions without punctation, where grooves would exist in female alloscutum. No distinct indentations, but dense punctation present where indentations would be in *B. hydrosauri*. Setation sparse overall, but setae become longer posteriorly. Eleven distinct festoons.

**Venter and coxae** (Figures S18 and S19). Striated, with evenly distributed, small, fine setae. Genital hood between coxa 2 and coxa 3, small, fine teeth-like structures present on posterior margin of genital hood. Shallow genital grooves terminate at anus. Ventral plaques absent. Posterior anal groove without lip, short anal suture to festoon margin. Coxae as in female.

**Tarsi** as in female.

**Remarks and differential diagnosis.** *B. spratti* n. sp. is one of nine species that parasitise echidnas in Australasia, four of which are *Bothriocroton*. The *B. concolor* group (*B. spratti* n. sp. and *B. concolor*) can be distinguished by the following characteristics: females with small, circular porose areas, and narrow, deep, corrugated cervical grooves; males with highly punctate conscutum, particularly in the posterior aspect, where the punctations are large and dense. Both sexes have wide palps that are markedly broader, proportionally, than in other species, and both with a characteristic ‘finger-like’ process on ventral palpal article 2 projecting past the medial margin.

Whereas the identification of the *B. concolor* group from *B. hydrosauri* group is straightforward, morphologically distinguishing *B. spratti* n. sp. and *B. concolor* is challenging. But these two species can be distinguished, in both sexes, by their cervical grooves. *B. concolor* cervical grooves have a shallow angle relative to the pinch point, curving to form the ‘neck’ of the intercervical area more gradually. In contrast, *B. spratti* n. sp. grooves are angled more sharply after the pinch point, resulting in a shorter groove, and a more elliptical intercervical area.

The northern limit of the geographic range of *B. spratti* n. sp. is not known; it will be somewhere south of Glen Innes, NSW, and north of Albury-Wodonga, NSW.

### 3.5. Dichotomous Key

**Figure 11:**
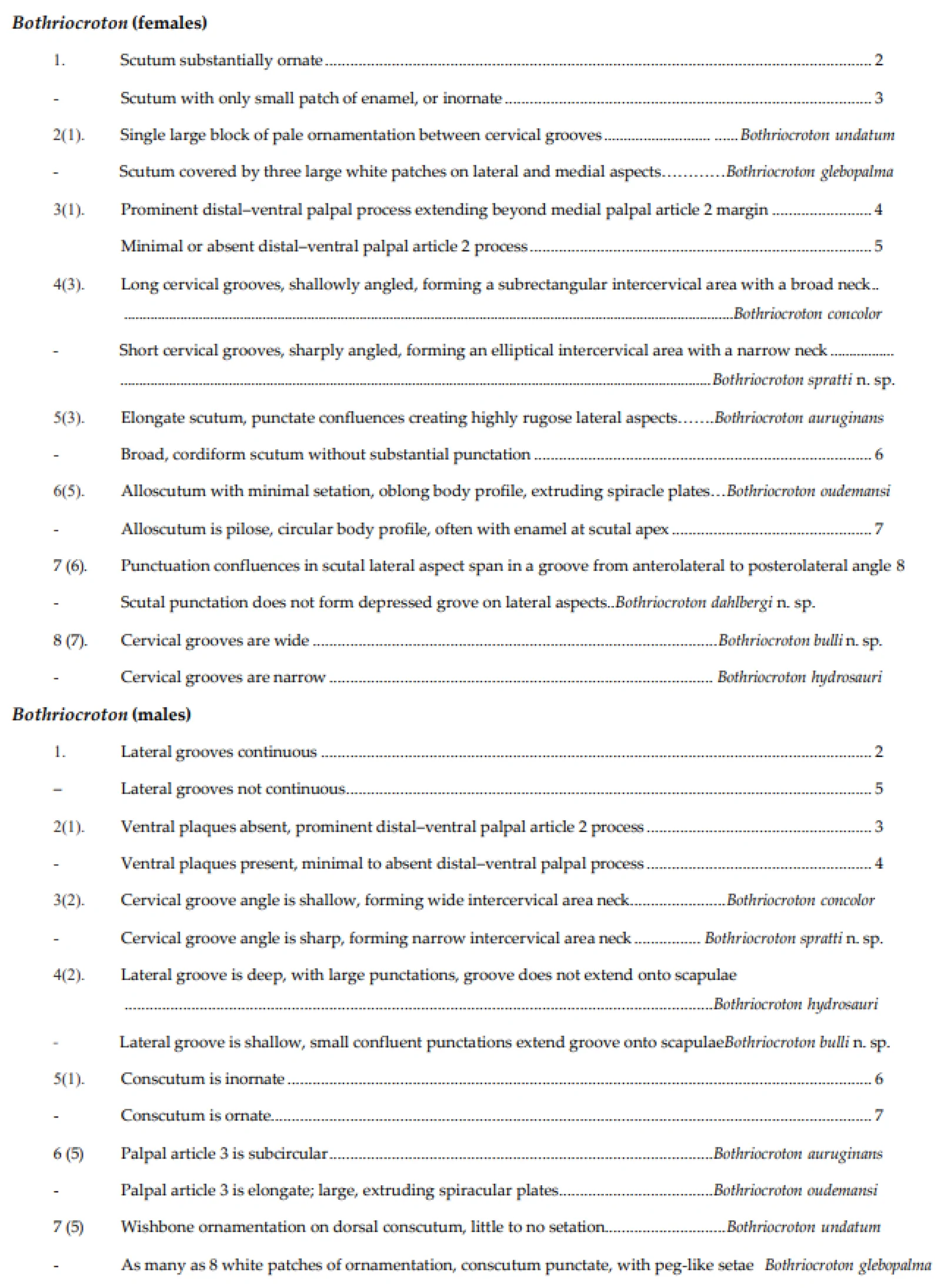
Dichotomous keys to adult female and male *Bothriocroton* ticks.

## 4. Discussion

**Figure 12:**
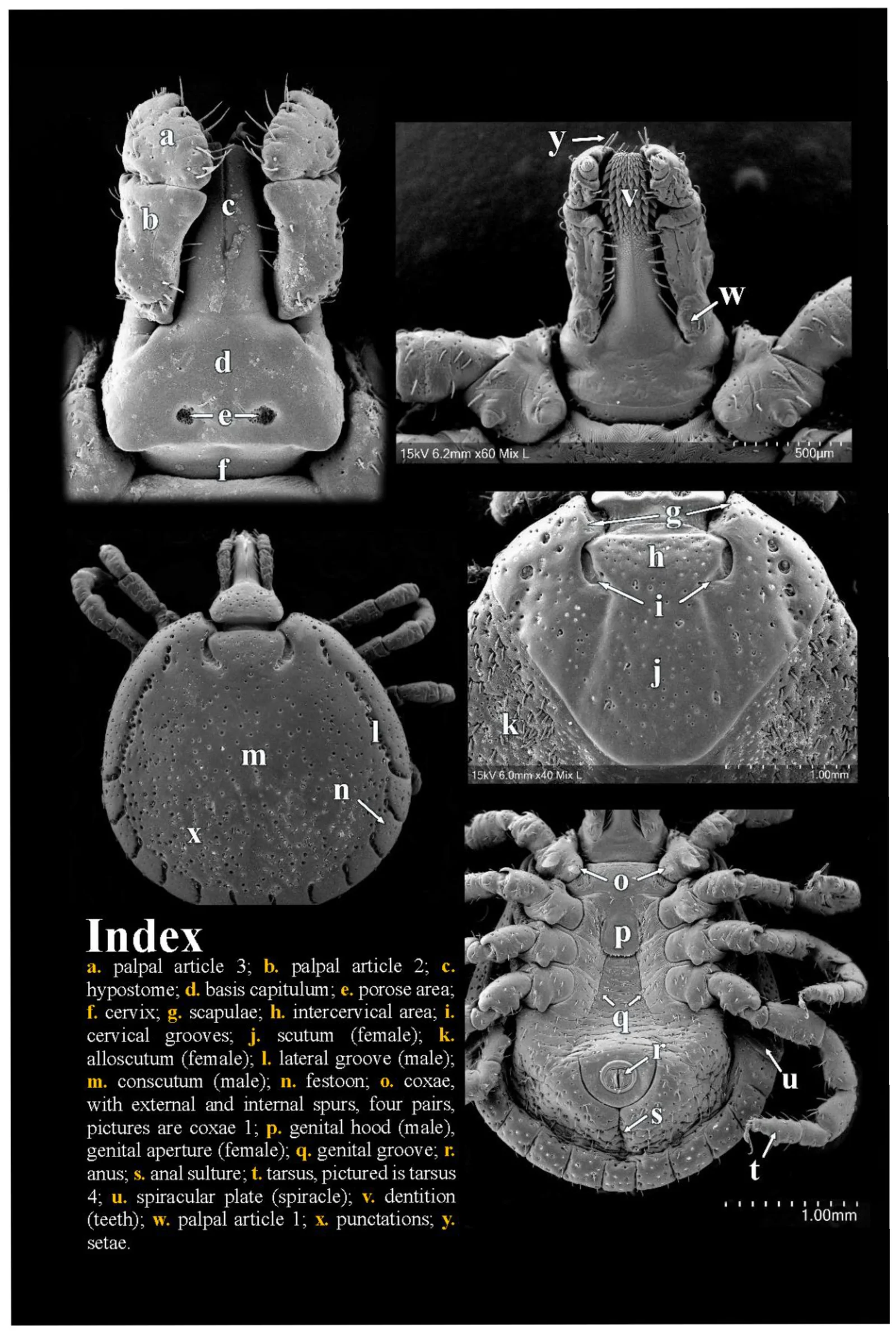
Terminology index.

Here we show how entire mitochondrial genomes (10,553 bp and 3512 amino acids) and nuclear rRNA sequences (18S rRNA, ITS1, ITS2 and 28S rRNA; 7510 bp) may be used to revise the taxonomy of ticks and thus to redescribe species, and to guide the discovery and description of new species. In our experience, morphology alone (Figure 12) may not always allow closely related species of ticks to be readily diagnosed. For example, the two species in the *B. concolor* group (*B. spratti* n. sp., *B. concolor* (Neumann, 1899)) had mitochondrial genomes that were 8–9% genetically different, yet with little morphological distinction. Similarly, *B. dahlbergi* n. sp. was as much as 17% different to *B. hydrosauri* (Denny, 1843) (Table 2), yet with little morphological distinction. On the other hand, the *B. hydrosauri* from Central Qld that infested echidnas are sufficiently morphologically different to the *B. hydrosauri* in southern Australia that infested reptiles to confuse experts like Roberts (1953) (but see Roberts 1970) and Andrews et al. (2006) [4,5,17]. Yet the *B. hydrosauri* from Central Qld that infested echidnas, which we genotyped (East End via Calliope, B8394; Damascus via Gin Gin, B8395), were only 0.97 to 1.22% different to the *B. hydrosauri* from southern Australia from reptiles (Table 2). Fortunately, the species of *Bothriocroton* that we studied can be distinguished unequivocally by their mitochondrial and nuclear rRNA genotypes.

In addition to morphological and genetic differences, historical observations provide evidence of behavioural differentiation among some of these populations. Andrews and Bull (1983) showed that males and females from geographically isolated Western Australia and South Australia populations of *B. hydrosauri* (previously identified as *Aponomma hydrosauri*) had differences in courtship behaviour: females failed to respond normally to males from the other population, with substantial differences in their copulatory behaviour too [52]. The Western Australian population examined in that study corresponds to the lineage recognised here as *B. bulli* n. sp. Although these observations were not interpreted at the time as evidence of separate species, they provide an additional indication of differentiation between the Western Australian and other *B. hydrosauri* populations.

At present, the genus *Bothriocroton* comprises nine extant species: *B. auruginans*, *B. bulli* n. sp., *B. concolor*, *B. dahlbergi* n. sp., *B. glebopalma*, *B. hydrosauri*, *B. oudemansi*, *B. spratti* n. sp. and *B. undatum*. At the time of writing, complete mitochondrial genomes were not available for *B. glebopalma* and *B. oudemansi*, although partial 12S, 16S and 18S rRNA sequences were available (Figures S1 and S2). We found no genetic evidence to support *B. tachyglossi* as a species. Unless contrary evidence is forthcoming, *B. tachyglossi* (Roberts, 1953) should be synonymised with *B. hydrosauri* (Denny, 1843) at some time in the future. The evolutionary relationships among several *Bothriocroton* species remain uncertain. This uncertainty is most apparent in the phylogenetic positions of *B. auruginans*, the wombat tick, and *B. undatum*, the goanna tick. Our mitochondrial phylogenies consistently had *B. undatum* as the sister to *B. auruginans* plus the *B. hydrosauri* group whereas the nuclear rRNA phylogenies had *B. auruginans* as the sister to *B. undatum* plus the *B. hydrosauri* group. Previous 18S rRNA trees had poor resolution of the deeper relationships within *Bothriocroton* [16,18]. In contrast, the mitochondrial genome trees in the present study are congruent with those reported by Kelava et al. (2024) [19].

Discordance between mitochondrial and nuclear phylogenies has on occasion been reported [53,54,55]. Mitochondrial genomes and nuclear rRNA markers differ fundamentally in their modes of inheritance and evolutionary histories, and consequently do not necessarily recover identical phylogenetic relationships [56,57]. Such discordance has been attributed to biological processes including incomplete lineage-sorting and introgression, although the present data do not allow these possibilities to be evaluated [58,59,60]. Mitochondrial introgression following hybridization is one possible explanation for discordance where mitochondrial genomes have a different evolutionary history from the nuclear genome [54,58]. This possibility may be particularly relevant to *Bothriocroton*, given the geographic and climatic history of Australia and the potential for changes in distribution and population connectivity associated with aridification to have brought previously separated populations into secondary contact. Aridification and changes in biome distribution have been implicated in diversification and population divergence in other Australian taxa [61]. Similar discordance between mitochondrial and nuclear phylogenies has also been reported in the tick genus *Rhipicephalus*, where historical hybridization and mitochondrial introgression have been considered as possible explanations [62]. Distinguishing between these alternatives will require population-level sampling across the geographic ranges of the relevant *Bothriocroton* species and sequencing of multiple independent nuclear loci or genome-wide markers. Such data would allow the extent of nuclear gene-tree discordance to be assessed and would provide a basis for testing alternative demographic histories, including ILS, historical gene flow and mitochondrial introgression. Comparisons of mitochondrial and nuclear genealogies across sympatric and allopatric populations, together with explicit tests for historical gene flow, could further determine whether mitochondrial lineages have crossed species boundaries independently of the nuclear genome. These analyses would provide a more robust assessment of whether the conflicting mitochondrial and nuclear relationships observed here reflect incomplete lineage-sorting, introgression, or a combination of these processes.

We further note with interest the marked difference in phylogenetic signal between the two datasets. Although the mitochondrial dataset (10,553 bp from the 13 protein-coding genes) and the nuclear rRNA dataset (7510 bp from 18S rRNA, ITS1, ITS2 and 28S rRNA) were of comparable lengths, the mitochondrial alignment contained 5637 variable, and thus potentially phylogenetically informative, sites compared with only 993 sites in the nuclear alignment (Figures S20 and S21). We expect that the substantially greater degree of sequence variation in the mitochondrial genomes provided greater phylogenetic resolution, and thus accounts for some of the conflicting relationships recovered between the two marker systems. Complete mitochondrial genomes and complete nuclear rRNA block sequences from *B. glebopalma* and *B. oudemansi* may provide additional context for resolving these deeper conflicting relationships. More broadly, we highlight the need for caution when inferring phylogenetic relationships from a single genetic locus.

Routine genotyping of ticks to confirm provisional identifications of species from morphology is a simple task in the light of the Barcode of Life Data Systems (BOLD). We recommend the submission of short (ca. 500 bp) nucleotide sequences of cytochrome oxidase 1 (*cox 1*) to BOLD when people cannot confidently identify *Bothriocroton* ticks by their morphology (or to check their morphological identifications). The following modified primers of Song et al. (2010) were predicted from in silico analyses to amplify the *Bothriocroton* species examined in this study: HCO1215 5′-GCCATTTTACCGCGATGA-3′ and HCO2064-SK 5′-GRTGAGCTCATACAATAAATC-3′ (Figure S22) [63].

## 5. Conclusions

Our phylogenetic trees, genetic distance matrices, species delimitation analyses (ABGD, bPTP and BPP), together with morphology, provide the most complete picture of the genus *Bothriocroton* so far. Three new species have been described in the *B. concolor* group (*B. spratti* n. sp.) and the *B. hydrosauri* group (*B. bulli* n. sp. and *B. dahlbergi* n. sp.). We have updated and improved the morphological dichotomous keys to male and female *Bothriocroton* species of Barker & Barker (2023), which were derived from those of Roberts (1970) [5,13]. The diversification of *Bothriocroton* appears to have occurred within a complex history of host association, geographic isolation and dispersal across Australia. Changes in host distributions and connectivity associated with the progressive aridification of the continent may have contributed to the isolation and subsequent secondary contact of tick populations. Further population-level genomic sampling across host species and geographic regions will be required to determine the relative contributions of host specificity, dispersal and climatic history to diversification within the genus.

## Figures and Tables

**Table 2:** Matrix of pairwise genetic differences (%) and Tamura–Nei distances (calculated in MEGA11) among 43 entire mitochondrial genomes (39 *Bothriocroton* sequences plus 2 *Haemaphysalis*, 1 *Alloceraea* and 1 *Archaeocroton* sequences). Cell colours represent a continuous green–yellow–red gradient, with green indicating lower values, yellow intermediate values, and red higher values. The colour gradient is scaled independently for pairwise genetic differences (%) and Tamura–Nei genetic distances and is intended as a visual aid for comparing relative values within each measure.

			1	2	3	4	5	6	7	8	9	10	11	12	13	14	15	16	17	18	19	20	21	22	23	24	25	26	27	28	29	30	31	32	33	34	35	36	37	38	39	40	41	42	43
**1**	***B*** **. *hydrosauri* B8395**	**QLD**	#	0.001	0.010	0.009	0.009	0.010	0.010	0.011	0.010	0.050	0.049	0.050	0.197	0.183	0.183	0.183	0.183	0.183	0.183	0.183	0.210	0.210	0.210	0.209	0.209	0.234	0.234	0.233	0.234	0.233	0.233	0.236	0.234	0.235	0.233	0.233	0.233	0.235	0.235	0.305	0.290	0.328	0.322
**2**	***B*** **. *hydrosauri* B8394**	**QLD**	0.19	#	0.011	0.010	0.010	0.011	0.011	0.011	0.010	0.050	0.049	0.050	0.197	0.183	0.183	0.184	0.183	0.183	0.183	0.183	0.209	0.210	0.209	0.209	0.209	0.234	0.234	0.233	0.234	0.233	0.233	0.235	0.233	0.235	0.233	0.233	0.233	0.235	0.235	0.305	0.290	0.328	0.321
**3**	*B. hydrosauri* OR350529	SA	1.09	1.18	#	0.011	0.009	0.001	0.012	0.012	0.012	0.050	0.050	0.051	0.197	0.183	0.183	0.183	0.183	0.183	0.183	0.183	0.209	0.210	0.209	0.208	0.208	0.236	0.236	0.235	0.236	0.235	0.235	0.236	0.234	0.235	0.233	0.233	0.233	0.235	0.235	0.306	0.290	0.326	0.324
**4**	***B*** **. *hydrosauri* B0643.M1**	**TAS**	1.13	1.22	1.28	#	0.003	0.011	0.001	0.002	0.001	0.049	0.049	0.051	0.197	0.182	0.182	0.182	0.181	0.182	0.181	0.181	0.208	0.209	0.208	0.208	0.208	0.233	0.233	0.232	0.233	0.232	0.232	0.235	0.233	0.234	0.232	0.232	0.232	0.234	0.234	0.304	0.288	0.325	0.321
**5**	***B*** **. *hydrosauri* B3165**	**TAS**	0.97	1.07	0.96	0.47	#	0.009	0.004	0.004	0.003	0.049	0.050	0.051	0.196	0.182	0.182	0.182	0.182	0.182	0.182	0.182	0.209	0.209	0.208	0.208	0.208	0.234	0.234	0.233	0.234	0.233	0.233	0.236	0.235	0.235	0.234	0.234	0.234	0.235	0.235	0.305	0.289	0.328	0.323
**6**	***B*** **. *hydrosauri* B7113.F1**	**VIC**	1.09	1.18	0.18	1.27	0.96	#	0.012	0.012	0.012	0.050	0.050	0.051	0.197	0.183	0.182	0.183	0.182	0.183	0.182	0.182	0.208	0.209	0.208	0.207	0.207	0.236	0.236	0.235	0.236	0.235	0.235	0.236	0.234	0.235	0.233	0.233	0.233	0.235	0.235	0.306	0.290	0.327	0.324
**7**	***B*** **. *hydrosauri* B7125.M1**	**VIC**	1.07	1.16	1.22	0.31	0.42	1.20	#	0.001	0.002	0.051	0.050	0.052	0.198	0.184	0.183	0.184	0.183	0.184	0.183	0.183	0.210	0.211	0.210	0.210	0.210	0.235	0.235	0.234	0.235	0.234	0.235	0.236	0.234	0.235	0.233	0.233	0.233	0.235	0.235	0.306	0.290	0.327	0.322
**8**	***B*** **. *hydrosauri* B7125.M2**	**VIC**	1.10	1.19	1.20	0.34	0.45	1.20	0.08	#	0.003	0.051	0.050	0.052	0.198	0.184	0.183	0.184	0.183	0.184	0.183	0.183	0.210	0.211	0.210	0.210	0.210	0.235	0.235	0.234	0.235	0.234	0.234	0.236	0.234	0.235	0.233	0.233	0.233	0.235	0.235	0.307	0.290	0.327	0.322
**9**	***B*** **. *hydrosauri* B8405**	**VIC**	1.03	1.13	1.18	0.22	0.36	1.17	0.24	0.27	#	0.050	0.050	0.052	0.198	0.184	0.184	0.184	0.183	0.184	0.183	0.183	0.210	0.211	0.210	0.210	0.210	0.235	0.235	0.234	0.235	0.234	0.234	0.236	0.234	0.235	0.233	0.234	0.234	0.235	0.235	0.306	0.290	0.327	0.323
**10**	***B*** **. *bulli* n. sp. B4931**	**WA**	4.82	4.79	4.83	4.96	4.83	4.86	4.94	4.94	4.90	#	0.008	0.009	0.199	0.183	0.182	0.183	0.182	0.183	0.182	0.182	0.211	0.212	0.211	0.210	0.210	0.236	0.237	0.237	0.237	0.237	0.236	0.236	0.235	0.235	0.234	0.234	0.234	0.235	0.235	0.306	0.290	0.325	0.322
**11**	***B*** **. *bulli* n. sp. B4918**	**WA**	4.83	4.87	4.90	5.00	4.89	4.94	4.94	4.94	4.94	0.92	#	0.002	0.198	0.181	0.181	0.181	0.180	0.181	0.180	0.180	0.210	0.210	0.210	0.209	0.209	0.235	0.236	0.235	0.236	0.236	0.235	0.234	0.234	0.233	0.233	0.233	0.233	0.234	0.233	0.304	0.290	0.323	0.321
**12**	***B*** **. *bulli* n. sp. B4922**	**WA**	4.94	4.91	5.03	5.11	5.00	5.05	5.07	5.07	5.05	0.97	0.36	#	0.199	0.182	0.182	0.182	0.182	0.182	0.182	0.182	0.211	0.211	0.211	0.210	0.210	0.237	0.237	0.237	0.237	0.237	0.237	0.235	0.234	0.234	0.233	0.233	0.233	0.234	0.234	0.304	0.290	0.324	0.322
**13**	***B*** **. *dahlbergi* n. sp. B7139.F1**	**QLD**	16.72	16.74	16.64	16.77	16.61	16.69	16.73	16.74	16.75	16.83	16.74	16.85	#	0.225	0.225	0.225	0.225	0.225	0.225	0.225	0.251	0.251	0.251	0.249	0.250	0.274	0.274	0.275	0.274	0.274	0.274	0.265	0.266	0.265	0.265	0.265	0.265	0.265	0.265	0.332	0.321	0.356	0.355
**14**	***B*** **. *auruginans* B6841**	**TAS**	15.64	15.68	15.63	15.71	15.59	15.58	15.66	15.67	15.68	15.66	15.53	15.63	18.61	#	0.003	0.003	0.003	0.003	0.003	0.003	0.218	0.218	0.218	0.218	0.218	0.243	0.243	0.243	0.243	0.242	0.242	0.242	0.240	0.241	0.240	0.240	0.240	0.241	0.241	0.317	0.294	0.336	0.333
**15**	*B*. *auruginans* B4505, OR350528	VIC	15.62	15.66	15.60	15.68	15.56	15.54	15.62	15.64	15.65	15.60	15.51	15.60	18.62	0.28	#	0.002	0.001	0.002	0.001	0.001	0.218	0.218	0.218	0.218	0.217	0.243	0.243	0.243	0.243	0.243	0.243	0.242	0.240	0.240	0.240	0.240	0.240	0.241	0.241	0.318	0.294	0.336	0.333
**16**	***B*** **. *auruginans* B7014.F1**	**VIC**	15.64	15.68	15.63	15.69	15.57	15.56	15.65	15.66	15.66	15.63	15.53	15.62	18.61	0.29	0.16	#	0.001	0.000	0.001	0.001	0.217	0.218	0.218	0.218	0.217	0.243	0.243	0.243	0.243	0.243	0.243	0.242	0.241	0.240	0.241	0.240	0.240	0.241	0.241	0.317	0.294	0.335	0.334
**17**	***B*** **. *auruginans* B7014.F2**	**VIC**	15.60	15.64	15.59	15.65	15.53	15.52	15.60	15.61	15.62	15.60	15.50	15.60	18.60	0.29	0.14	0.07	#	0.001	0.000	0.000	0.217	0.218	0.218	0.217	0.217	0.243	0.243	0.243	0.243	0.242	0.242	0.242	0.240	0.240	0.240	0.240	0.240	0.241	0.240	0.317	0.293	0.336	0.333
**18**	***B*** **. *auruginans* B7014.N1**	**VIC**	15.63	15.67	15.62	15.69	15.56	15.56	15.64	15.66	15.66	15.62	15.53	15.62	18.60	0.29	0.16	0.01	0.07	#	0.001	0.001	0.217	0.218	0.218	0.217	0.217	0.243	0.243	0.243	0.243	0.242	0.243	0.242	0.241	0.240	0.240	0.240	0.240	0.241	0.241	0.317	0.294	0.335	0.334
**19**	***B*** **. *auruginans* B7014.N3**	**VIC**	15.60	15.64	15.59	15.65	15.53	15.52	15.60	15.61	15.62	15.60	15.50	15.60	18.60	0.29	0.14	0.07	0.00	0.07	#	0.000	0.217	0.218	0.218	0.217	0.217	0.243	0.243	0.243	0.243	0.242	0.242	0.242	0.240	0.240	0.240	0.240	0.240	0.241	0.240	0.317	0.293	0.336	0.333
**20**	***B*** **. *auruginans* B7285.F2**	**VIC**	15.60	15.64	15.59	15.65	15.53	15.52	15.60	15.61	15.62	15.60	15.50	15.60	18.60	0.29	0.14	0.07	0.00	0.07	0.00	#	0.217	0.218	0.218	0.217	0.217	0.243	0.243	0.243	0.243	0.242	0.242	0.242	0.240	0.240	0.240	0.240	0.240	0.241	0.240	0.317	0.293	0.336	0.333
**21**	***B*** **. *undatum* B8356B**	**QLD**	17.56	17.55	17.51	17.57	17.49	17.47	17.59	17.57	17.57	17.70	17.65	17.72	20.38	18.13	18.13	18.10	18.10	18.09	18.10	18.10	#	0.003	0.002	0.017	0.016	0.269	0.267	0.267	0.268	0.267	0.268	0.261	0.259	0.260	0.259	0.259	0.259	0.260	0.260	0.327	0.305	0.341	0.346
**22**	*B*. *undatum* NC_017757	QLD	17.59	17.57	17.57	17.62	17.54	17.52	17.64	17.63	17.62	17.75	17.67	17.73	20.35	18.17	18.17	18.14	18.14	18.13	18.14	18.14	0.36	#	0.004	0.018	0.017	0.268	0.267	0.267	0.268	0.267	0.267	0.260	0.258	0.259	0.258	0.258	0.258	0.259	0.259	0.327	0.304	0.342	0.347
**23**	***B*** **. *undatum* B8140.M1**	**QLD**	17.56	17.56	17.51	17.59	17.48	17.45	17.60	17.59	17.59	17.72	17.67	17.74	20.34	18.17	18.17	18.14	18.14	18.13	18.14	18.14	0.25	0.43	#	0.017	0.017	0.269	0.268	0.268	0.268	0.268	0.268	0.261	0.259	0.260	0.259	0.259	0.259	0.260	0.260	0.325	0.305	0.341	0.346
**24**	***B*** **. *undatum* B3263**	**VIC**	17.55	17.54	17.46	17.56	17.46	17.41	17.57	17.56	17.55	17.60	17.56	17.63	20.25	18.14	18.14	18.10	18.10	18.10	18.10	18.10	1.72	1.77	1.78	#	0.001	0.270	0.268	0.268	0.269	0.268	0.269	0.260	0.259	0.259	0.259	0.259	0.259	0.260	0.260	0.324	0.305	0.340	0.346
**25**	***B*** **. *undatum* B5053a.N1**	**VIC**	17.53	17.53	17.45	17.54	17.44	17.40	17.56	17.54	17.53	17.60	17.56	17.63	20.29	18.12	18.11	18.08	18.08	18.07	18.08	18.08	1.66	1.73	1.73	0.16	#	0.270	0.268	0.268	0.269	0.268	0.269	0.260	0.259	0.259	0.259	0.259	0.259	0.260	0.260	0.326	0.305	0.341	0.346
**26**	***B*** **. *concolor* B6868.AU439**	**NSW**	19.18	19.23	19.30	19.24	19.21	19.31	19.27	19.25	19.24	19.35	19.26	19.39	21.72	19.68	19.73	19.71	19.71	19.71	19.71	19.71	21.37	21.34	21.43	21.40	21.41	#	0.004	0.004	0.004	0.004	0.003	0.090	0.089	0.088	0.088	0.088	0.088	0.089	0.089	0.326	0.319	0.348	0.346
**27**	***B*** **. *concolor* [PNG] B0895.N7**	**PNG**	19.17	19.22	19.31	19.24	19.20	19.32	19.26	19.24	19.24	19.36	19.28	19.40	21.73	19.68	19.73	19.71	19.71	19.71	19.71	19.71	21.30	21.26	21.35	21.33	21.35	0.44	#	0.004	0.004	0.004	0.004	0.090	0.090	0.089	0.089	0.089	0.089	0.089	0.089	0.325	0.319	0.349	0.345
**28**	***B*** **. *concolor* B7019.F10**	**QLD**	19.15	19.20	19.27	19.21	19.17	19.29	19.23	19.20	19.20	19.37	19.29	19.42	21.79	19.70	19.75	19.74	19.73	19.73	19.73	19.73	21.30	21.28	21.36	21.34	21.36	0.42	0.47	#	0.002	0.001	0.001	0.090	0.089	0.088	0.089	0.089	0.089	0.089	0.089	0.326	0.318	0.348	0.345
**29**	***B*** **. *concolor* B8484.AU483**	**QLD**	19.20	19.25	19.32	19.26	19.21	19.32	19.26	19.25	19.25	19.40	19.32	19.45	21.77	19.68	19.72	19.71	19.70	19.70	19.70	19.70	21.35	21.31	21.39	21.37	21.39	0.40	0.46	0.18	#	0.002	0.001	0.090	0.090	0.089	0.089	0.089	0.089	0.089	0.089	0.326	0.319	0.349	0.345
**30**	***B*** **. *concolor* B3095**	**QLD**	19.15	19.20	19.27	19.21	19.17	19.29	19.23	19.20	19.20	19.38	19.30	19.43	21.76	19.67	19.71	19.70	19.69	19.69	19.69	19.69	21.31	21.28	21.36	21.34	21.35	0.40	0.47	0.15	0.17	#	0.001	0.090	0.090	0.089	0.089	0.089	0.089	0.089	0.089	0.326	0.319	0.348	0.345
**31**	***B*** **. *concolor* B8145**	**QLD**	19.16	19.21	19.29	19.24	19.19	19.29	19.24	19.23	19.23	19.38	19.30	19.43	21.74	19.67	19.72	19.70	19.70	19.70	19.70	19.70	21.34	21.31	21.39	21.37	21.39	0.33	0.39	0.14	0.12	0.14	#	0.089	0.089	0.088	0.088	0.088	0.088	0.088	0.088	0.326	0.319	0.349	0.345
**32**	***B*** **. *spratti* n. sp. B8632.AU473**	**NSW**	19.58	19.57	19.57	19.64	19.60	19.59	19.58	19.57	19.61	19.57	19.47	19.56	21.50	19.91	19.92	19.91	19.89	19.91	19.89	19.89	21.16	21.13	21.21	21.12	21.13	8.45	8.48	8.45	8.49	8.49	8.41	#	0.012	0.001	0.011	0.011	0.011	0.001	0.002	0.318	0.312	0.345	0.342
**33**	***B*** **. *spratti* n. sp. B6897, AU439**	**SA**	19.18	19.17	19.16	19.23	19.24	19.20	19.19	19.18	19.20	19.25	19.17	19.25	21.24	19.50	19.52	19.54	19.52	19.54	19.52	19.52	20.79	20.75	20.84	20.78	20.79	8.07	8.13	8.09	8.14	8.13	8.06	1.49	#	0.011	0.002	0.002	0.002	0.011	0.011	0.318	0.313	0.344	0.341
**34**	*B*. *spratti* n. sp. NC 017756	SA	19.24	19.24	19.23	19.28	19.25	19.25	19.23	19.22	19.26	19.22	19.12	19.20	21.17	19.52	19.52	19.52	19.50	19.51	19.50	19.50	20.83	20.81	20.90	20.81	20.81	8.02	8.06	8.03	8.08	8.07	8.00	0.50	1.13	#	0.011	0.011	0.011	0.001	0.001	0.319	0.312	0.343	0.341
**35**	***B*** **. *spratti* n. sp. B5478**	**SA**	19.14	19.14	19.12	19.19	19.18	19.15	19.14	19.13	19.16	19.19	19.11	19.19	21.21	19.52	19.54	19.54	19.52	19.54	19.52	19.52	20.79	20.74	20.84	20.78	20.78	8.01	8.07	8.03	8.08	8.07	8.00	1.44	0.24	1.09	#	0.001	0.001	0.011	0.011	0.317	0.312	0.343	0.342
**36**	***B*** **. *spratti* n. sp. B5481**	**SA**	19.14	19.14	19.12	19.19	19.18	19.16	19.15	19.14	19.16	19.18	19.10	19.18	21.20	19.48	19.50	19.52	19.50	19.51	19.50	19.50	20.77	20.72	20.82	20.76	20.76	8.00	8.07	8.01	8.06	8.05	7.98	1.42	0.24	1.08	0.10	#	0.000	0.010	0.010	0.316	0.312	0.343	0.341
**37**	***B*** **. *spratti* n. sp. B5485**	**SA**	19.14	19.14	19.12	19.19	19.18	19.16	19.15	19.14	19.16	19.18	19.10	19.18	21.20	19.48	19.50	19.52	19.50	19.51	19.50	19.50	20.77	20.72	20.82	20.76	20.76	8.00	8.07	8.01	8.06	8.05	7.98	1.42	0.24	1.08	0.10	0.00	#	0.010	0.010	0.316	0.312	0.343	0.341
**38**	***B*** **. *spratti* n. sp. B8141**	**VIC**	19.25	19.25	19.24	19.28	19.27	19.26	19.23	19.22	19.26	19.24	19.14	19.23	21.19	19.56	19.56	19.56	19.54	19.55	19.54	19.54	20.83	20.80	20.89	20.81	20.81	8.04	8.07	8.05	8.09	8.09	8.01	0.48	1.12	0.12	1.06	1.05	1.05	#	0.001	0.319	0.313	0.344	0.342
**39**	***B*** **. *spratti* n. sp. B5461**	**VIC**	19.24	19.24	19.23	19.28	19.25	19.25	19.23	19.22	19.26	19.22	19.12	19.20	21.21	19.54	19.54	19.54	19.52	19.54	19.52	19.52	20.84	20.81	20.90	20.81	20.81	8.04	8.08	8.05	8.10	8.09	8.02	0.51	1.12	0.14	1.06	1.05	1.05	0.11	#	0.319	0.313	0.343	0.342
**40**	*Archaeocroton sphenodonti* NC_017745	NZ	24.03	24.06	24.13	24.09	24.03	24.10	24.10	24.11	24.07	24.12	23.98	24.05	25.60	24.74	24.75	24.73	24.72	24.72	24.72	24.72	25.28	25.32	25.21	25.17	25.22	25.16	25.10	25.15	25.18	25.16	25.16	25.00	24.71	24.76	24.67	24.65	24.65	24.78	24.76	#	0.274	0.317	0.315
**41**	*Alloceraea inermis* OL741739	Romania	23.23	23.24	23.21	23.21	23.16	23.19	23.18	23.17	23.17	23.19	23.22	23.24	24.99	23.37	23.35	23.37	23.35	23.37	23.35	23.35	24.09	24.07	24.09	24.10	24.12	24.78	24.79	24.76	24.81	24.79	24.78	24.67	24.42	24.41	24.40	24.39	24.39	24.42	24.46	22.06	#	0.282	0.280
**42**	*Haemaphysalis flava* OL741741	Japan	25.56	25.55	25.45	25.50	25.52	25.46	25.50	25.48	25.49	25.41	25.31	25.38	27.04	25.98	25.94	25.93	25.94	25.93	25.94	25.94	26.32	26.33	26.32	26.22	26.28	26.62	26.62	26.58	26.65	26.61	26.62	26.60	26.31	26.29	26.29	26.30	26.30	26.33	26.31	24.67	22.69	#	0.222
**43**	*Haemaphysalis longicornis* OL741744	Japan	25.16	25.18	25.25	25.22	25.21	25.26	25.17	25.16	25.23	25.19	25.13	25.22	27.01	25.80	25.81	25.83	25.77	25.83	25.77	25.77	26.57	26.59	26.58	26.54	26.56	26.53	26.46	26.46	26.50	26.47	26.48	26.56	26.27	26.27	26.30	26.28	26.28	26.31	26.30	24.53	22.62	18.53	#

**Table 3:** Matrix of average species pairwise genetic differences (%) and Tamura–Nei distances (calculated in MEGA11) among the 39 entire mitochondrial genomes of *Bothriocroton*, grouped by species. The minimum and maximum values are in brackets.

	*B. hydrosauri*	*B. bulli*	*B. dahlbergi*	*B. auruginans*	*B. undatum*	*B. concolor*	*B. spratti*
***B. hydrosauri***	**0.86** (0.08–1.28)						
***B. bulli***	**4.94** (4.79–5.11)	**0.75** (0.36–0.97)					
***B. dahlbergi***	**16.71** (16.61–16.77)	**16.81** (16.74–16.85)	-				
***B. auruginans***	**15.62** (15.52–15.71)	**15.58** (15.5–15.66)	**18.61** (18.6–18.62)	**0.14** (0–0.29)			
***B. undatum***	**17.54** (17.4–17.64)	**17.66** (17.56–17.75)	**20.32** (20.25–20.38)	**18.12** (18.07–18.17)	**1.16** (0.16–1.78)		
***B. concolor***	**19.23** (19.15–19.32)	**19.36** (19.26–19.45)	**21.75** (21.72–21.79)	**19.71** (19.67–19.75)	**21.35** (21.26–21.43)	**0.31** (0.12–0.47)	
***B. spratti***	**19.25** (19.12–19.64)	**19.22** (19.1–19.57)	**21.24** (21.17–21.5)	**19.57** (19.48–19.92)	**20.84** (20.72–21.21)	**8.1** (7.98–8.49)	**0.77** (0–1.49)

**Table 4:** Bayesian species delimitation analyses (A11) executed in the Bayesian Phylogenetics and Phylogeography program (BPP) under alternative combinations of population size (θ) and divergence time (τ) priors. Values shown for θ Mean and τ Mean represent the prior means of the inverse gamma distributions used in each analysis. Posterior probabilities (PP) are shown for the two most frequently recovered delimitation models: a seven-species model and a six-species model in which *Bothriocroton bulli* and *B. hydrosauri* are treated as a single species. The preferred model for each analysis was the delimitation model with the highest posterior probability.

Analysis	θ Prior	θ Mean	τ Prior	τ Mean	PP 7 Species	PP 6 Species (*B. bulli* + *B. hydrosauri*)	Preferred Model
**A11_1**	IG(3, 0.002)	0.001	IG(3, 0.02)	0.01	0.571	0.407	7 species
**A11_2**	IG(3, 0.002)	0.001	IG(3, 0.05)	0.025	0.427	0.562	6 species
**A11_3**	IG(3, 0.005)	0.0025	IG(3, 0.02)	0.01	0.887	0.112	7 species
**A11_4**	IG(3, 0.005)	0.0025	IG(3, 0.05)	0.025	0.843	0.157	7 species
**A11_5**	IG(3, 0.010)	0.005	IG(3, 0.02)	0.01	0.921	0.078	7 species
**A11_6**	IG(3, 0.010)	0.005	IG(3, 0.05)	0.025	0.919	0.081	7 species

## Data Availability

All alignments, tree files and XML files of the current work are available through Mendeley Data. DOI: 10.17632/43wjt87jrh.2.

## Supplementary Materials

The following supporting information can be downloaded at: https://www.mdpi.com/article/10.3390/biology15191733/s1, Table S1: Best-fit partitioning schemes; Table S2: Summary of MrBayes convergence statistics; Figure S1: 12S rRNA phylogenetic tree; Figure S2: 16S rRNA phylogenetic tree; Figure S3: ITS phylogenetic tree; Figure S4: STARBEAST tree; Figure S5: SEM female gnathosoma (dorsal); Figure S6: SEM female gnathosoma (ventral); Figure S7: SEM female gnathosoma basis (dorsal); Figure S8: SEM female scutum; Figure S9: SEM female cervical groove; Figure S10: SEM female alloscutum; Figure S11; SEM female anterior venter; Figure S12: SEM female posterior venter; Figure S13: SEM male gnathosoma (dorsal); Figure S14: SEM male gnathosoma (ventral); Figure S15: SEM male anterior conscutum; Figure S16: SEM male cervical grooves; Figure S17: SEM male posterior conscutum; Figure S18: SEM male anterior venter; Figure S19: SEM male posterior venter; Figure S20: MT genome variable sites; Figure S21: rDNA variable sites; Figure S22: *cox1* alignment.
