# Supplementary material for: How Phylogenies Inferred from 46 Mitochondrial Genomes Informed the Systematics of the Australasian Genus of Ticks, Bothriocroton, with the Description of Three New Species
Source: Biology (Basel). 2026 Oct 1;15(19):1733. doi: 10.3390/biology15191733 (PMC13663443; doi:10.3390/biology15191733)
Supplement: Supplementary file 1 [file biology-15-01733-s001.zip › biology-4467241-supplementary.pdf]

Table S1: Best-fit partitioning schemes selected by PartitionFinder for the 13 mitochondrial protein-coding gene (PCG) nucleotide and amino acid alignments, as well as the 18S+ITS1+ITS2+28S DNA alignments. Codon base triplets are denoted pos1, pos2 and pos3.

MT 13 PCG DNA sequence partitions.

Subset	Best Model Iqtree	MrBayes Model	Partition Names
1	GTR+I+G	GTR+I+G	NAD5_pos1, NAD3_pos1, ATP6_pos1
2	GTR+I+G	GTR+I+G	ATP6_pos2
3	HKY+I+G	HKY+I+G	ATP6_pos3, NAD4_pos3, NAD3_pos3
4	TRN+I+G	GTR+I+G	ATP8_pos1, NAD2_pos1, NAD6_pos1
5	HKY+G	HKY+G	ATP8_pos2
6	HKY+G	HKY+G	ATP8_pos3, NAD6_pos3, NAD2_pos3
7	GTR+G	GTR+G	COX1_pos1
8	GTR+I+G	GTR+I+G	COX1_pos2
9	TRN+I+G	GTR+I+G	COX1_pos3, COX2_pos3
10	GTR+I+G	GTR+I+G	COX2_pos1, CYTB_pos1
11	GTR+I+G	GTR+I+G	COX2_pos2
12	TVM+I	GTR+I	COX3_pos1
13	K81UF+I	GTR+I	COX3_pos2
14	HKY+G	HKY+G	NAD4L_pos3, COX3_pos3
15	K81UF+I+G	GTR+I+G	CYTB_pos2
16	HKY+G	HKY+G	CYTB_pos3
17	GTR+I+G	GTR+I+G	NAD4_pos1, NAD1_pos1, NAD4L_pos1
18	GTR+I+G	GTR+I+G	NAD4L_pos2, NAD3_pos2, NAD5_pos2, NAD1_pos2, NAD4_pos2
19	GTR+I+G	GTR+I+G	NAD5_pos3, NAD1_pos3
20	GTR+G	GTR+G	NAD2_pos2, NAD6_pos2

MT 13 PCG AA sequences partitions.

Subset	Best Model Iqtree	MrBayes Model	Partition Names
1	MTART+I+G+F	WAG+I+G	ATP6, NAD3, NAD1, NAD4
2	MTMAM+G+F	MTMAM+G	ATP8, NAD2
3	MTMAM+I+G+F	MTMAM+I+G	COX1
4	MTMAM+I+G+F	MTMAM+I+G	COX2, NAD4L, CYTB, COX3
5	MTREV+I+G+F	MTREV+I+G	NAD5, NAD6

18S+ITS1+ITS2+28S DNA sequence partitions.

Subset	Best Model Iqtree	MrBayes Model	Partition Names
1	K81UF+I	GTR+I	18S
2	TVM+G	GTR+G	ITS1
3	GTR+G	GTR+G	ITS2
4	GTR+I+G	GTR+I+G	28S

COX1 DNA

Subset	Best Model Iqtree	MrBayes Model	Partition Names
1	TIM2+F+I+G	GTR+I+G	COX1

12S rRNA DNA

Subset	Best Model Iqtree	MrBayes Model	Partition Names
1	TVM+F+I+G	GTR+I+G	12S rRNA

16S rRNA DNA

Subset	Best Model Iqtree	MrBayes Model	Partition Names
1	K3Pu+F+G	GTR+G	16S rRNA

ITS1+ITS2 DNA sequence partitions.

Subset	Best Model Iqtree	MrBayes Model	Partition Names
1	TVM+G	GTR+G	ITS1
2	GTR+G	GTR+G	ITS2

Table S2: Summary of MrBayes convergence statistics for all phylogenetic datasets. The average standard deviation of split frequencies (ASDSF < 0.01) and average Potential Scale Reduction Factor (PSRF ≈ 1.0) indicate highly supported convergence across all Bayesian inference (BI) runs. A minimum ESS >200 indicates adequate sampling and stability of model parameters.

Dataset / Analysis	Average Standard Deviation of Split Frequencies	Average PSRF	Minimum ESS
**46_13PCG_DNA_BI**	0.000776	1	496
**46_13PCG_AA_BI**	0.008821	1.0001	842
**30_18S_ITS1_ITS2_28S_BI**	0.00657	1.001	468
**75_COX1_DNA_BI**	0.008335	1	1299
**70_16S_DNA_BI**	0.007061	1.0004	907
**80_12S_DNA_BI**	0.00567	1	1858
**30_ITS1_ITS2_BI**	0.005986	1	1295

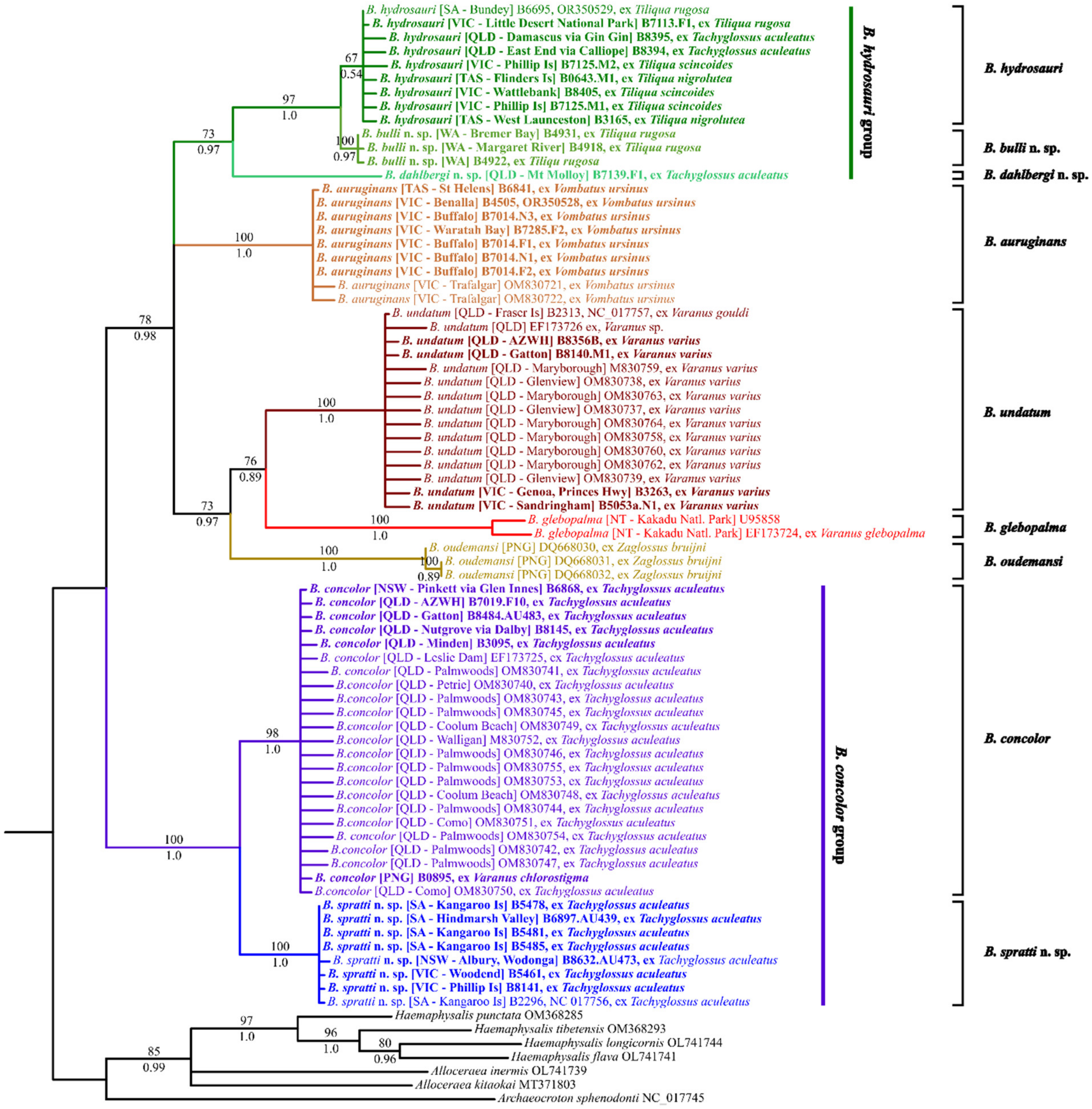

Figure S1. Phylogenetic tree inferred from 80 nucleotide sequences derived from the mitochondrial *12S rRNA* gene. The final alignment was 378 bp. Maximum Likelihood (ML) bootstrap supports are shown above branches, whereas Bayesian Inference (BI) posterior probability supports are shown below branches. Coloured fonts and labels indicate putative clades inferred from the mitochondrial genome tree (Figure 1). Bold font indicates the 35 mitochondrial genome sequences published in the present paper. Abbreviations: Qld, Queensland; NSW, New South Wales; NT, Northern Territory; Vic, Victoria; WA, Western Australia; SA, South Australia; Tas, Tasmania; PNG, Papua New Guinea.

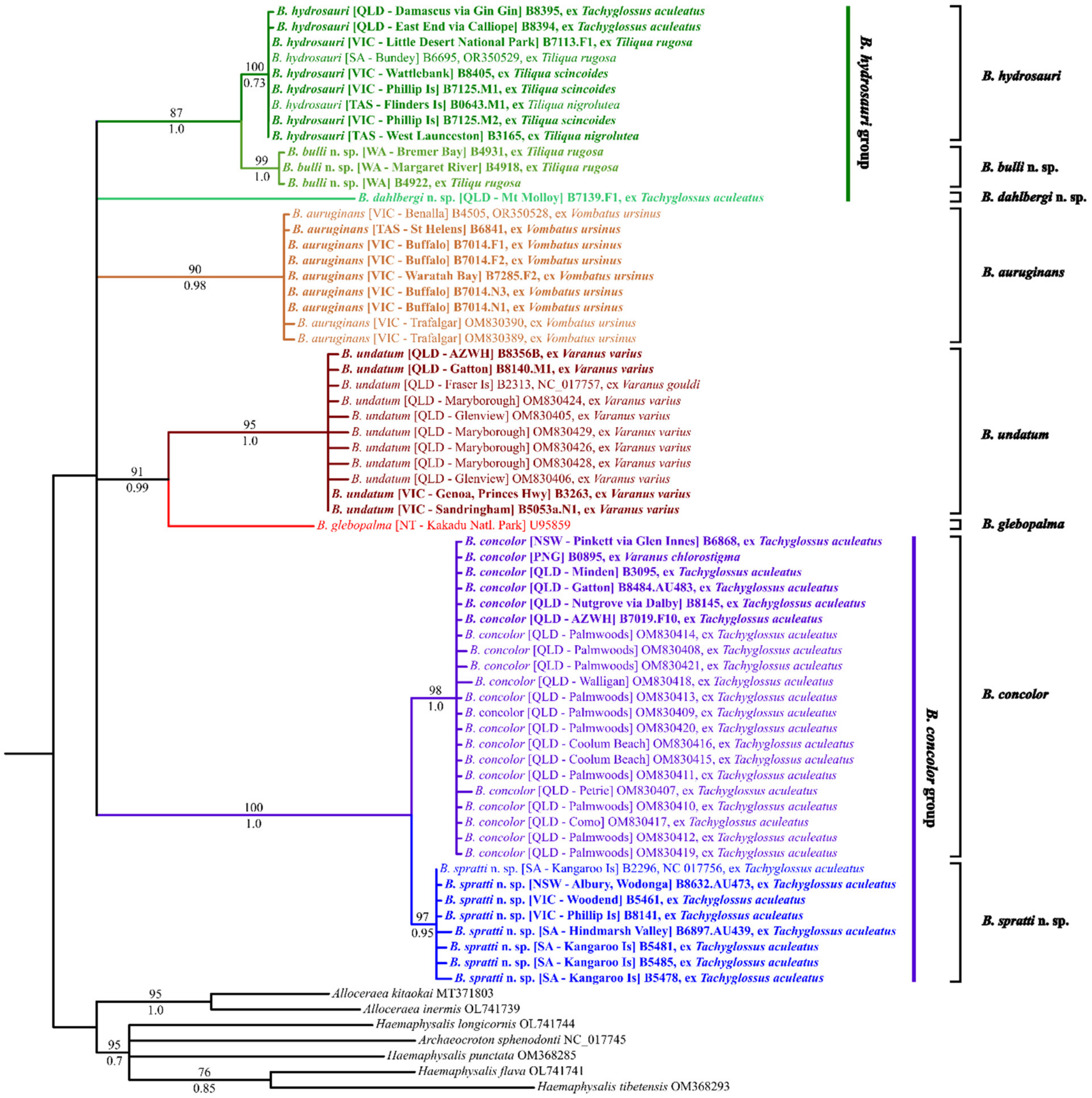

Figure S2. Phylogenetic tree inferred from 70 nucleotide sequences derived from the mitochondrial *16S rRNA* gene. The final alignment was 425 bp. Maximum Likelihood (ML) bootstrap supports are shown above branches, whereas Bayesian Inference (BI) posterior probability supports are shown below branches. Coloured fonts and labels indicate putative clades inferred from the mitochondrial genome tree (Figure 1). Bold font indicates the 35 mitochondrial genome sequences published in the present paper. Abbreviations: Qld, Queensland; NSW, New South Wales; NT, Northern Territory; Vic, Victoria; WA, Western Australia; SA, South Australia; Tas, Tasmania; PNG, Papua New Guinea.

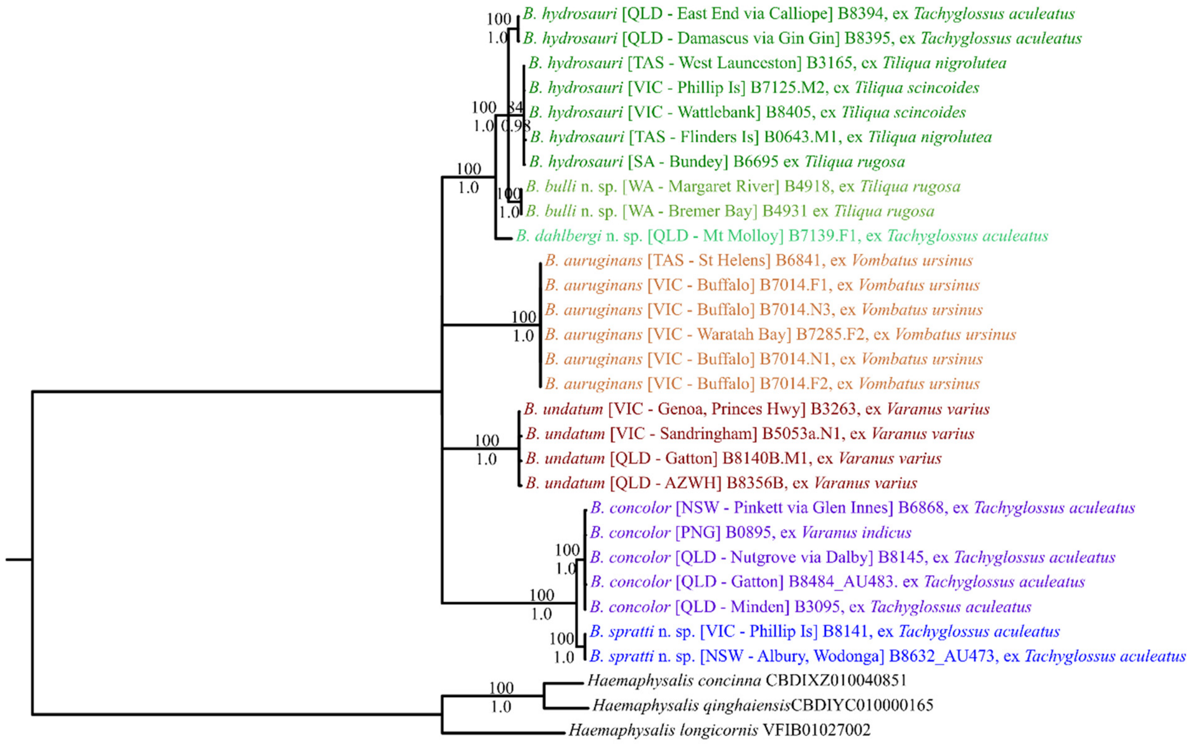

Figure S3. Phylogenetic tree inferred from 30 nucleotide sequences derived from the ITS1 and ITS2 genes. The final alignment was 3,003 bp. Maximum Likelihood (ML) bootstrap supports are shown above branches, whereas Bayesian Inference (BI) posterior probability supports are shown below branches. Coloured fonts and labels indicate putative clades inferred from the mitochondrial genome tree (Figure 1). Bold font indicates the 27 rRNA sequences published in the present paper. Abbreviations: Qld, Queensland; NSW, New South Wales; Vic, Victoria; WA, Western Australia; SA, South Australia; Tas, Tasmania; PNG, Papua New Guinea.

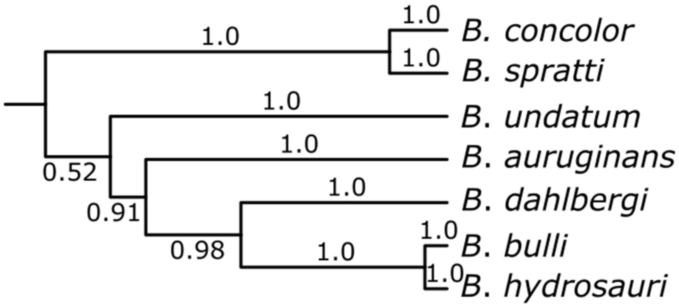

Figure S4. Species tree from the multispecies coalescent analyses in STARBEAST from the mitochondrial genome (10,553 bp) plus nuclear rRNA nucleotides (7,510 bp). Numbers above and below branches: posterior probabilities.

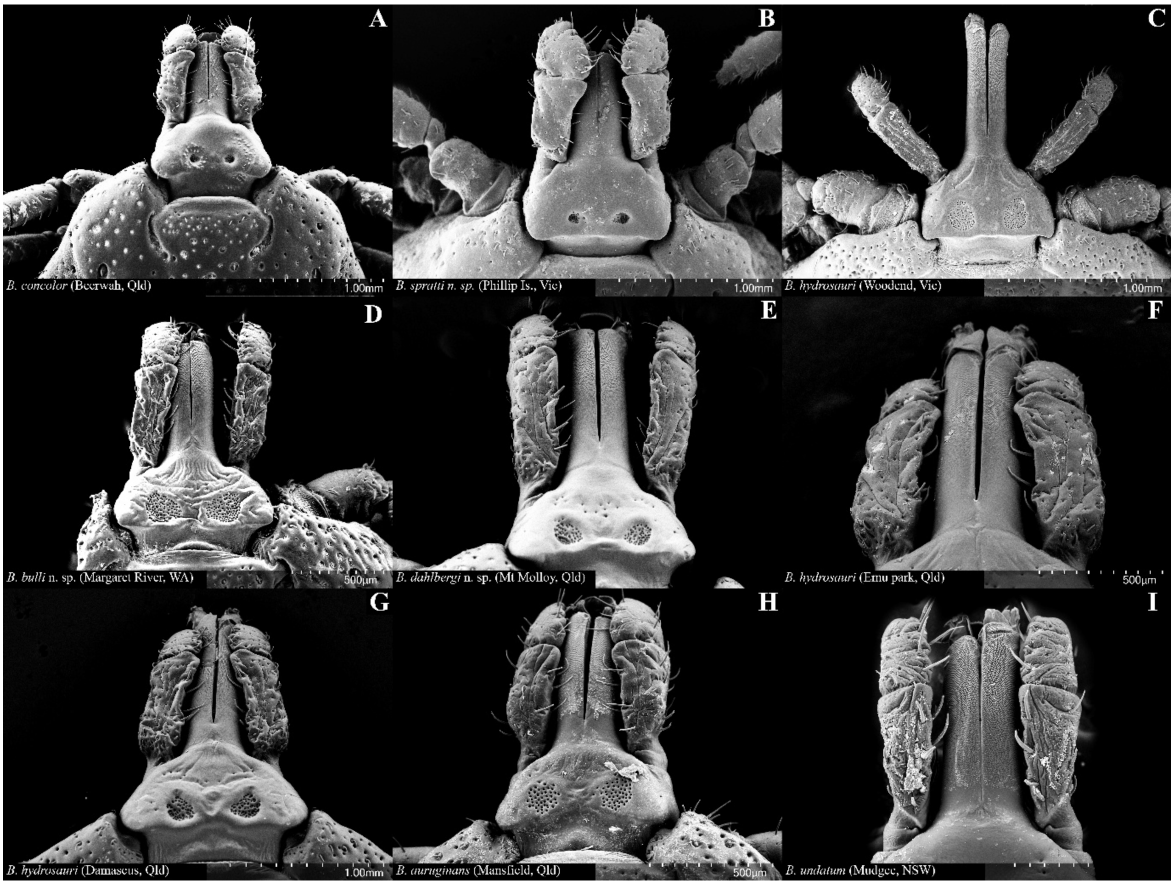

Figure S5. Scanning electron micrographs of the gnathosoma (dorsal view) of females of the nine *Bothriocroton* species ((A) *B. concolor*, Australia Zoo Wildlife Hospital, Qld, B8060 ex *Tachyglossus aculeatus*; (B) *B. spratti* n. sp., Phillip Is, Vic, B8141 ex *T. aculeatus*; (C) *B. hydrosauri,* Woodend, Vic, B4210C ex *Tiliqua* sp.; (D) *B. bulli* n. sp., Margaret River, WA, B4918 ex *Tiliqua rugosa*; (E) *B. dahlbergi* n. sp., Mt Molloy, Qld, B7139 ex *T. aculeatus*; (F) *B. hydrosauri*, Emu Park, Qld, B9104 ex *T. aculeatus*; (G) *B. hydrosauri*, Damascus via Gin Gin, Qld, B8395 ex *T. aculeatus*; (H) *B. auruginans*, Mansfield, Vic, B3349 ex *Vombatus ursinus*; (I) *B. undatum,* Mudgee, NSW, B4026 ex *Varanus varius*. The surprising finding of *B. hydrosauri* in Qld led us to study this species by SEM from three localities: (C) Woodend, Vic; (F) Emu Park, Qld; (G) Damascus via Gin Gin, Qld.

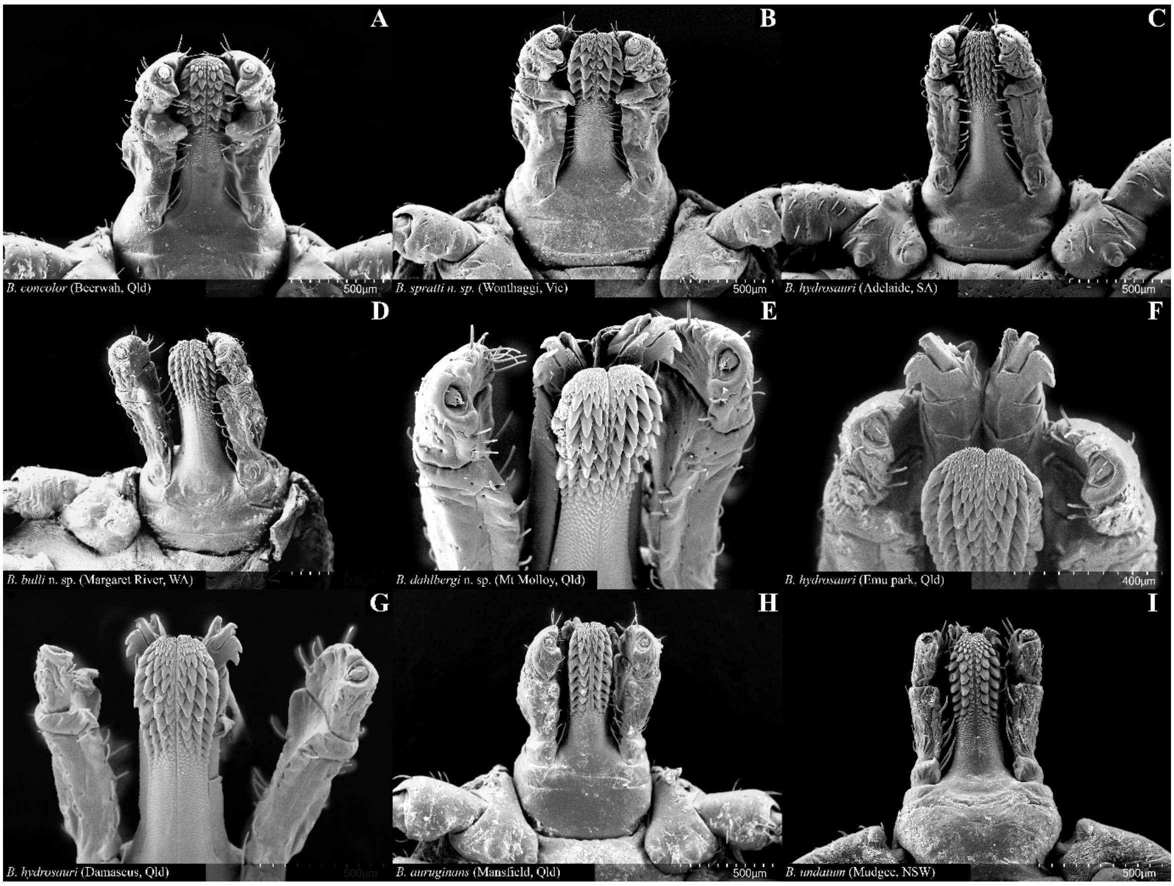

Figure S6. Scanning electron micrographs of the gnathosoma (ventral view) of females the nine *Bothriocroton* species ((A) *B. concolor*, Australia Zoo Wildlife Hospital, Qld, B8060 ex *Tachyglossus aculeatus*; (B) *B. spratti* n. sp., Wonthaggi, Vic, B8215 ex *T. aculeatus*; (C) *B. hydrosauri*, Hallett Cove Conservation Park, Adelaide, SA, B6930 ex *Tiliqua* sp.; (D) *B. bulli* n. sp., Margaret River, WA, B4918 ex *Tiliqua rugosa*; (E) *B. dahlbergi* n. sp., Mt Molloy, Qld, B7139 ex *T. aculeatus*; (F) *B. hydrosauri*, Emu Park, Qld, B9104 ex *T. aculeatus*; (G) *B. hydrosauri*, Damascus via Gin Gin, Qld, B8395 ex *T. aculeatus*; (H) *B. auruginans*, Mansfield, Vic, B3349 ex *Vombatus ursinus*; (I) *B. undatum,* Mudgee, NSW, B4026 ex *Varanus varius*. The surprising finding of *B. hydrosauri* in Qld led us to study this species by SEM from three localities: (C) Adelaide, SA; (F) Emu Park, Qld; (G) Damascus via Gin Gin, Qld.

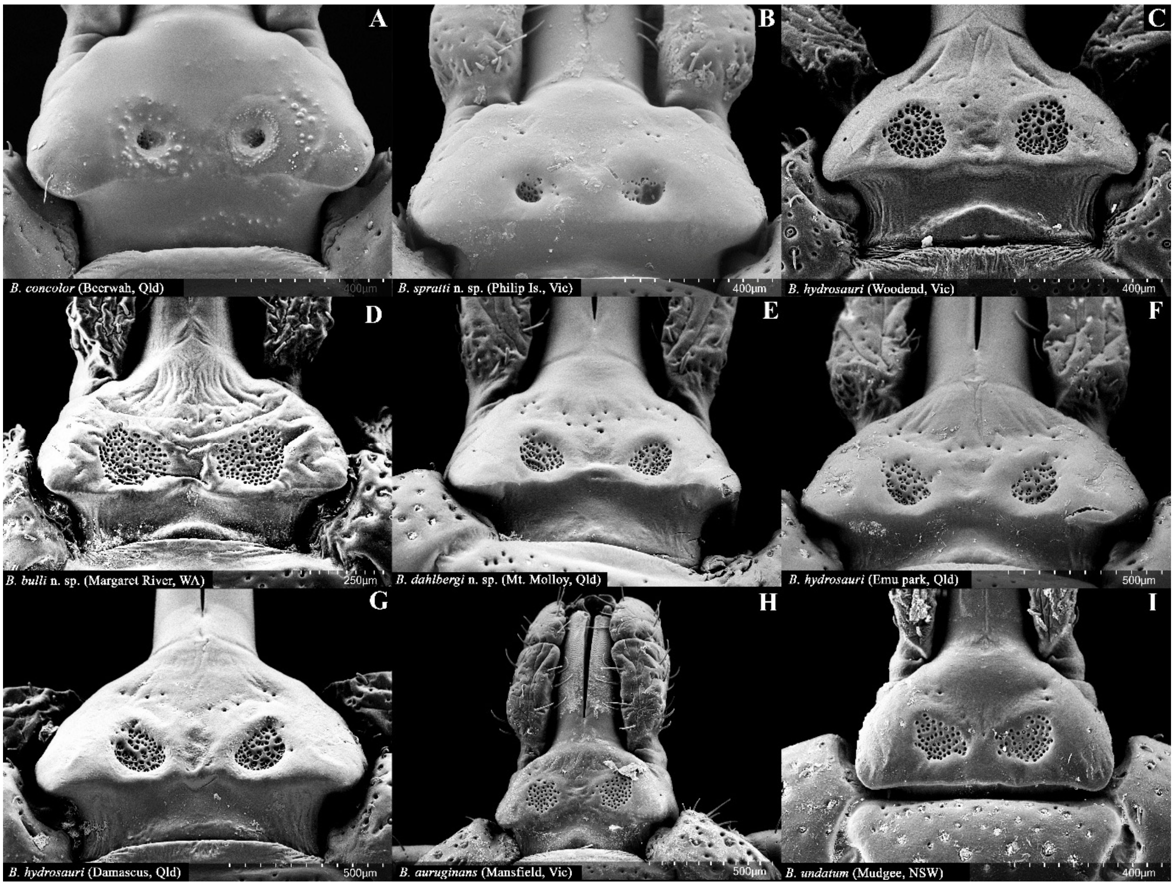

Figure S7. Scanning electron micrographs of the gnathosoma basis (dorsal view) of females of the nine *Bothriocroton* species ((A) *B. concolor*, Australia Zoo Wildlife Hospital, Qld, B8060 ex *Tachyglossus aculeatus*; (B) *B. spratti* n. sp., Phillip Is, Vic, B8141 ex *T. aculeatus*; (C) *B. hydrosauri,* Woodend, Vic, B4210C ex *Tiliqua* sp.; (D) *B. bulli* n. sp., Margaret River, WA, B4918 ex *Tiliqua rugosa*; (E) *B. dahlbergi* n. sp., Mt Molloy, Qld, B7139 ex *T. aculeatus*; (F) *B. hydrosauri*, Emu Park, Qld, B9104 ex *T. aculeatus*; (G) *B. hydrosauri*, Damascus via Gin Gin, Qld, B8395 ex *T. aculeatus*; (H) *B. auruginans*, Mansfield, Vic, B3349 ex *Vombatus ursinus*; (I) *B. undatum,* Mudgee, NSW, B4026 ex *Varanus varius*. The surprising finding of *B. hydrosauri* in Qld led us to study this species by SEM from three localities: (C) Woodend, Vic; (F) Emu Park, Qld; (G) Damascus via Gin Gin, Qld.

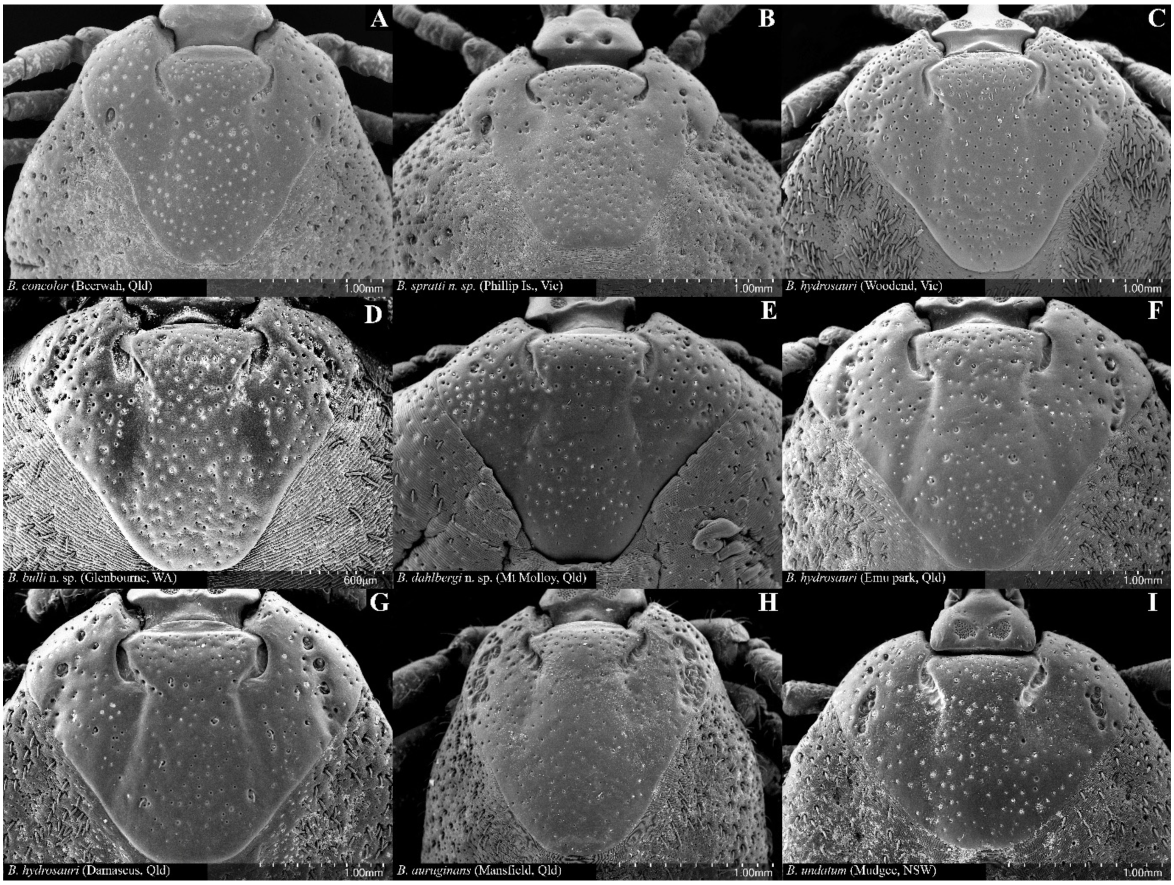

Figure S8. Scanning electron micrographs of the scutum (dorsal view) of females of the nine *Bothriocroton* species ((A) *B. concolor*, Australia Zoo Wildlife Hospital, Qld, B8060 ex *Tachyglossus aculeatus*; (B) *B. spratti* n. sp., Phillip Is, Vic, B8141 ex *T. aculeatus*; (C) *B. hydrosauri,* Woodend, Vic, B4210C ex *Tiliqua* sp.; (D) *B. bulli* n. sp., Glenbourne, WA, B3185 ex *Tiliqua rugosa*; (E) *B. dahlbergi* n. sp., Mt Molloy, Qld, B7139 ex *T. aculeatus*; (F) *B. hydrosauri*, Emu Park, Qld, B9104 ex *T. aculeatus*; (G) *B. hydrosauri*, Damascus via Gin Gin, Qld, B8395 ex *T. aculeatus*; (H) *B. auruginans*, Mansfield, Vic, B3349 ex *Vombatus ursinus*; (I) *B. undatum,* Mudgee, NSW, B4026 ex *Varanus varius*. The surprising finding of *B. hydrosauri* in Qld led us to study this species by SEM from three localities: (C) Woodend, Vic; (F) Emu Park, Qld; (G) Damascus via Gin Gin, Qld.

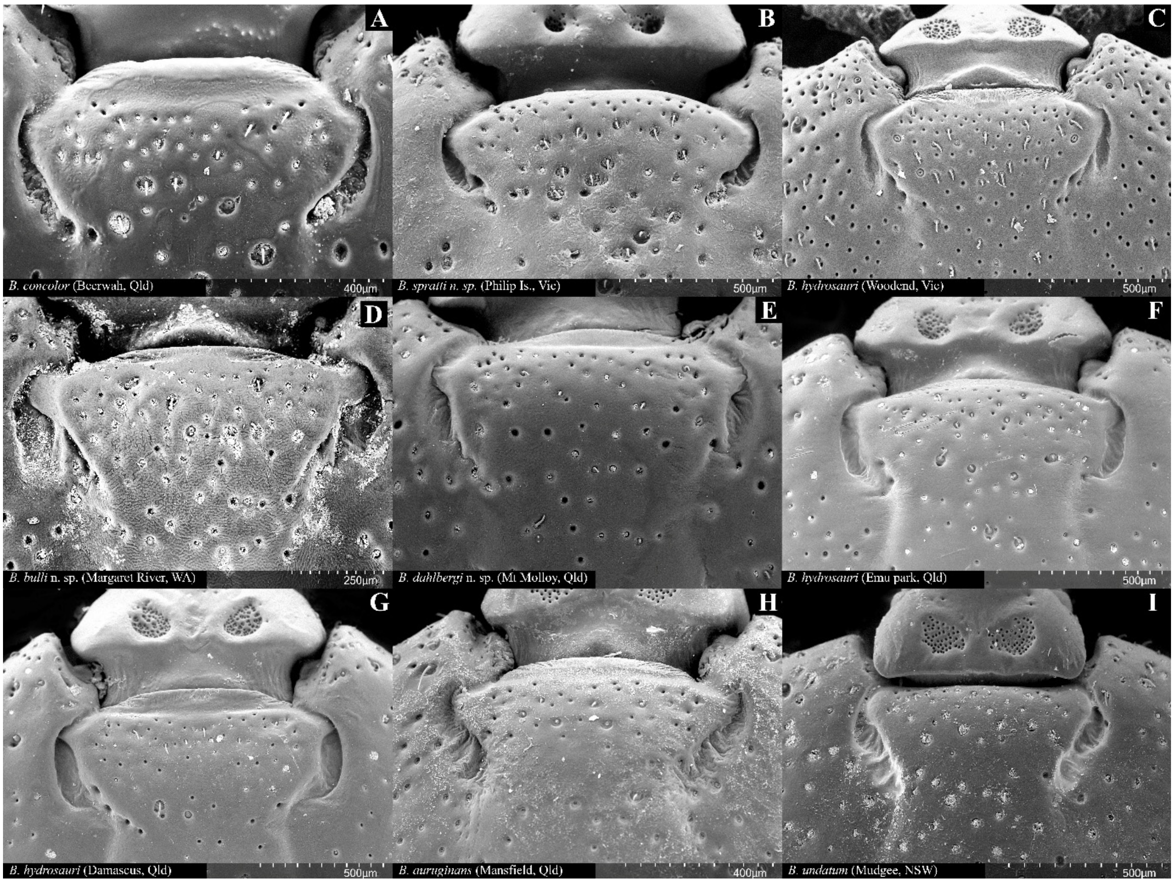

Figure S9. Scanning electron micrographs of the cervical groove of females of the nine *Bothriocroton* species ((A) *B. concolor*, Australia Zoo Wildlife Hospital, Qld, B8060 ex *Tachyglossus aculeatus*; (B) *B. spratti* n. sp., Phillip Is, Vic, B8141 ex *T. aculeatus*; (C) *B. hydrosauri,* Woodend, Vic, B4210C ex *Tiliqua* sp.; (D) *B. bulli* n. sp., Glenbourne, WA, B3185 ex *Tiliqua rugosa*; (E) *B. dahlbergi* n. sp., Mt Molloy, Qld, B7139 ex *T. aculeatus*; (F) *B. hydrosauri*, Emu Park, Qld, B9104 ex *T. aculeatus*; (G) *B. hydrosauri*, Damascus via Gin Gin, Qld, B8395 ex *T. aculeatus*; (H) *B. auruginans*, Mansfield, Vic, B3349 ex *Vombatus ursinus*; (I) *B. undatum,* Mudgee, NSW, B4026 ex *Varanus varius*. The surprising finding of *B. hydrosauri* in Qld led us to study this species by SEM from three localities: (C) Woodend, Vic; (F) Emu Park, Qld; (G) Damascus via Gin Gin, Qld.

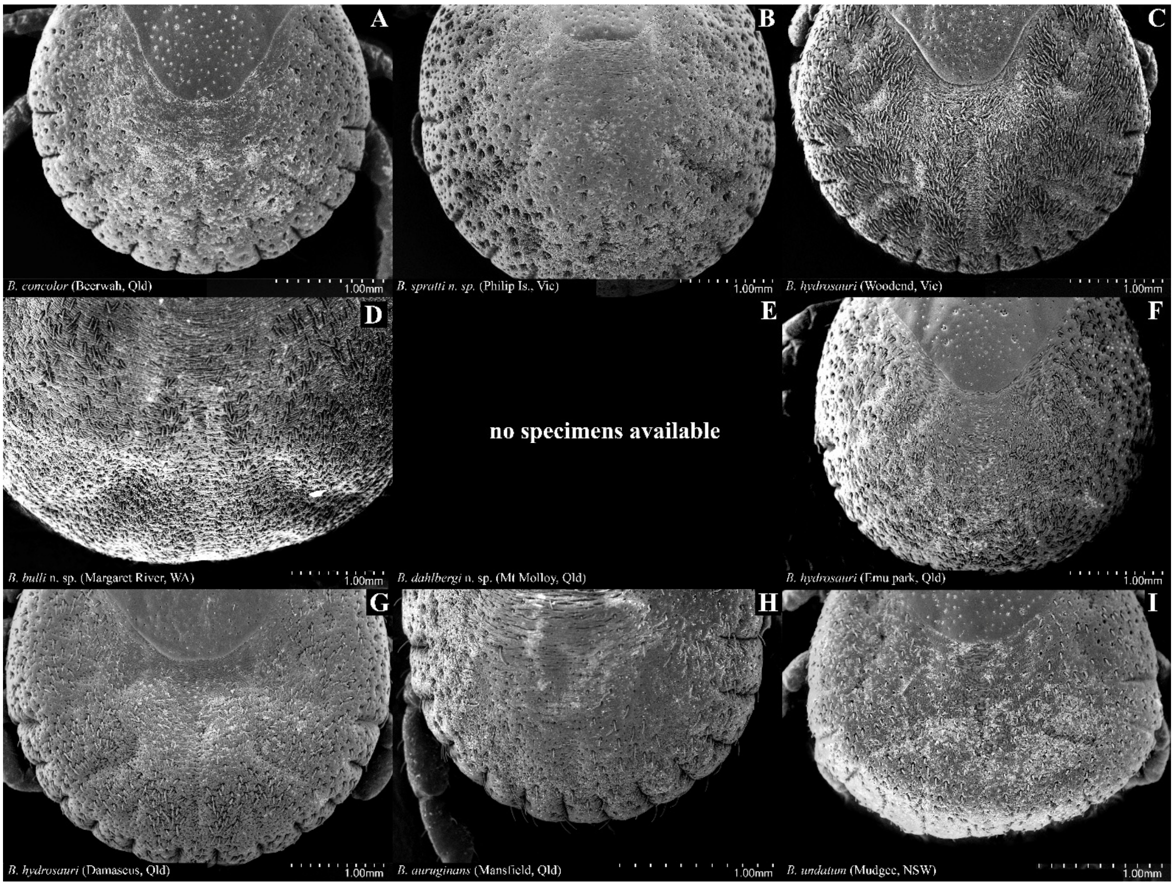

Figure S10. Scanning electron micrographs of the alloscutum of females of the nine *Bothriocroton* species ((A) *B. concolor*, Australia Zoo Wildlife Hospital, Qld, B8060 ex *Tachyglossus aculeatus*; (B) *B. spratti* n. sp., Phillip Is, Vic, B8141 ex *T. aculeatus*; (C) *B. hydrosauri,* Woodend, Vic, B4210C ex *Tiliqua* sp.; (D) *B. bulli* n. sp., Glenbourne, WA, B3185 ex *Tiliqua rugosa*; (E) *B. dahlbergi* n. sp., specimen unavailable; (F) *B. hydrosauri*, Emu Park, Qld, B9104 ex *T. aculeatus*; (G) *B. hydrosauri*, Damascus via Gin Gin, Qld, B8395 ex *T. aculeatus*; (H) *B. auruginans*, Mansfield, Vic, B3349 ex *Vombatus ursinus*; (I) *B. undatum,* Mudgee, NSW, B4026 ex *Varanus varius*. The surprising finding of *B. hydrosauri* in Qld led us to study this species by SEM from three localities: (C) Woodend, Vic; (F) Emu Park, Qld; (G) Damascus via Gin Gin, Qld.

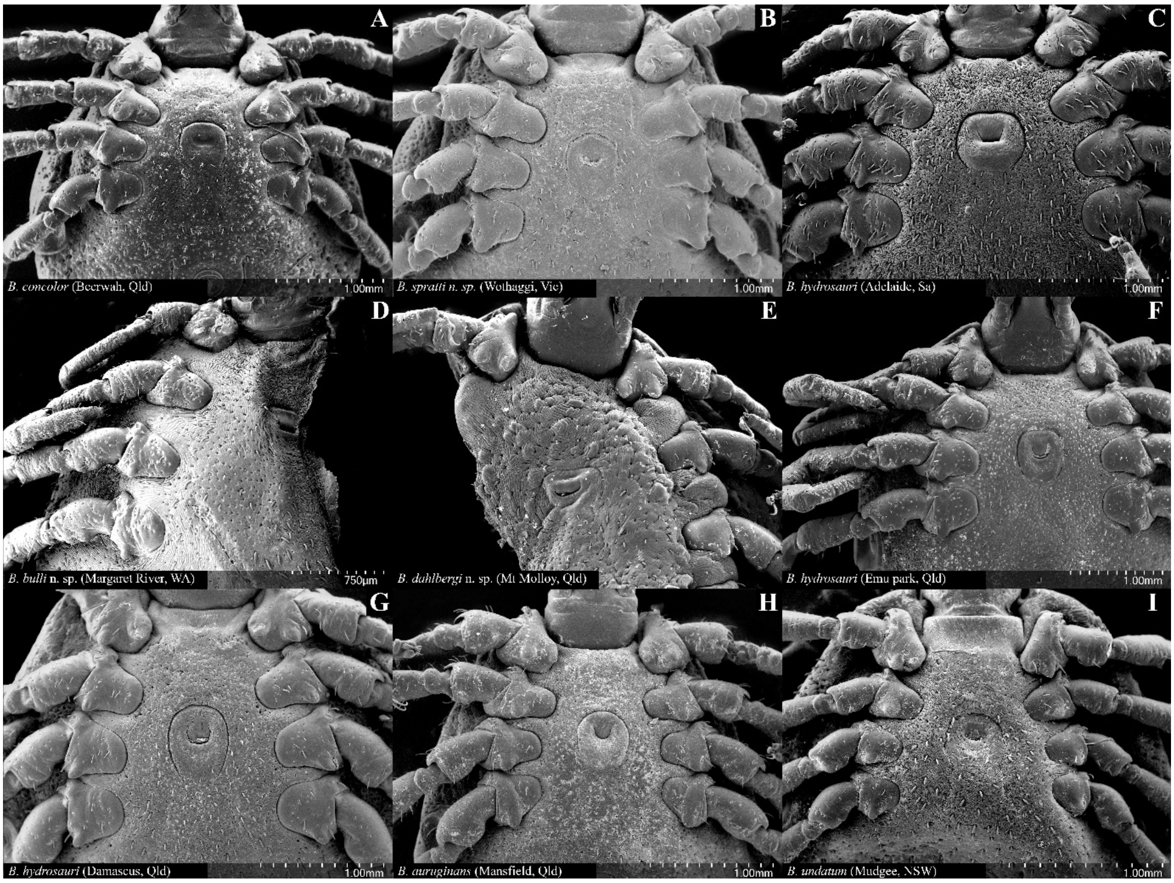

Figure S11. Scanning electron micrographs of the anterior venter of females of the nine *Bothriocroton* species ((A) *B. concolor*, Australia Zoo Wildlife Hospital, Qld, B8060 ex *Tachyglossus aculeatus*; (B) *B. spratti* n. sp., Wonthaggi, Vic, B8215 ex *T. aculeatus*; (C) *B. hydrosauri*, Hallett Cove Conservation Park, Adelaide, SA, B6930 ex *Tiliqua* sp.; (D) *B. bulli* n. sp., Margaret River, WA, B4918 ex *Tiliqua rugosa*; (E) *B. dahlbergi* n. sp., Mt Molloy, Qld, B7139 ex *T. aculeatus*; (F) *B. hydrosauri*, Emu Park, Qld, B9104 ex *T. aculeatus*; (G) *B. hydrosauri*, Damascus via Gin Gin, Qld, B8395 ex *T. aculeatus*; (H) *B. auruginans*, Mansfield, Vic, B3349 ex *Vombatus ursinus*; (I) *B. undatum,* Mudgee, NSW, B4026 ex *Varanus varius*. The surprising finding of *B. hydrosauri* in Qld led us to study this species by SEM from three localities: (C) Adelaide, SA; (F) Emu Park, Qld; (G) Damascus via Gin Gin, Qld.

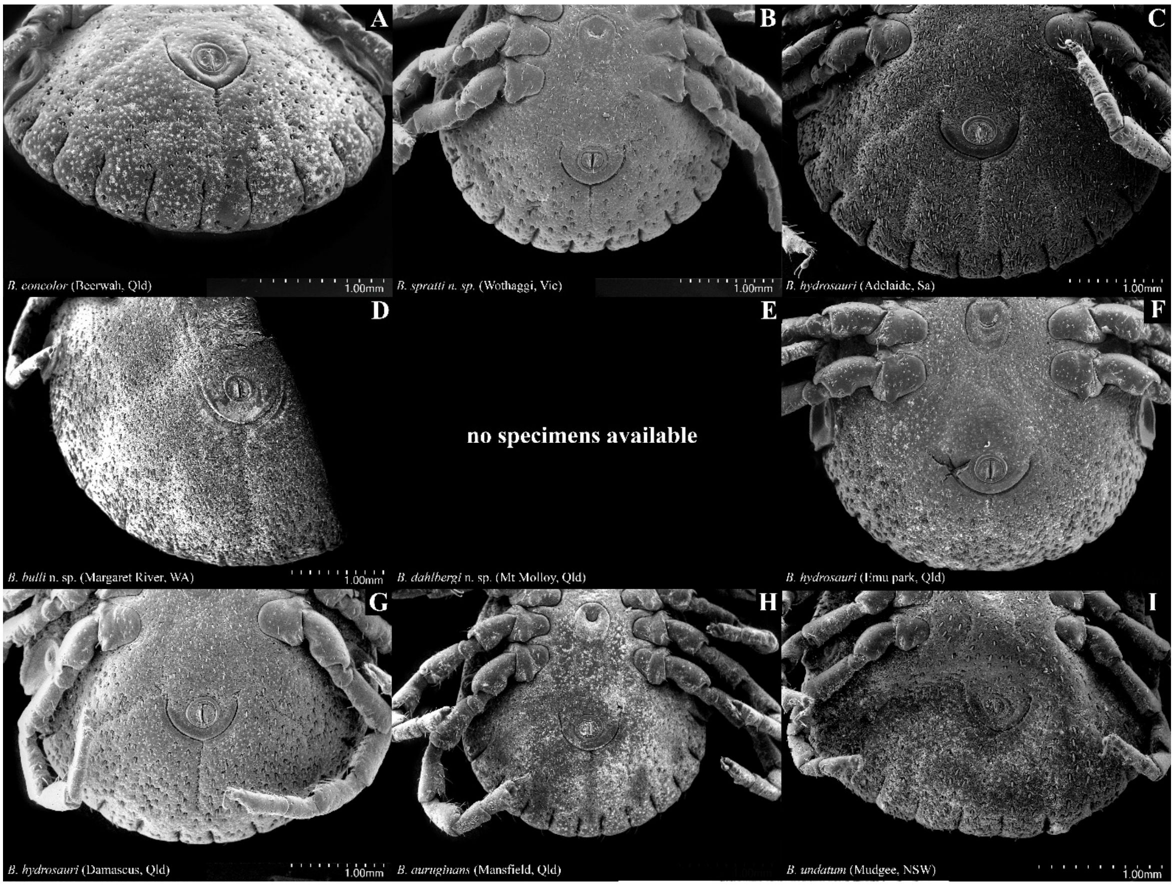

Figure S12. Scanning electron micrographs of the posterior venter of females of the nine *Bothriocroton* species ((A) *B. concolor*, Australia Zoo Wildlife Hospital, Qld, B8060 ex *Tachyglossus aculeatus*; (B) *B. spratti* n. sp., Wonthaggi, Vic, B8215 ex *T. aculeatus*; (C) *B. hydrosauri*, Hallett Cove Conservation Park, Adelaide, SA, B6930 ex *Tiliqua* sp.; (D) *B. bulli* n. sp., Margaret River, WA, B4918 ex *Tiliqua rugosa*; (E) *B. dahlbergi* n. sp., specimen unavailable; (F) *B. hydrosauri*, Emu Park, Qld, B9104 ex *T. aculeatus*; (G) *B. hydrosauri*, Damascus via Gin Gin, Qld, B8395 ex *T. aculeatus*; (H) *B. auruginans*, Mansfield, Vic, B3349 ex *Vombatus ursinus*; (I) *B. undatum,* Mudgee, NSW, B4026 ex *Varanus varius*. The surprising finding of *B. hydrosauri* in Qld led us to study this species by SEM from three localities: (C) Adelaide, SA; (F) Emu Park, Qld; (G) Damascus via Gin Gin, Qld.

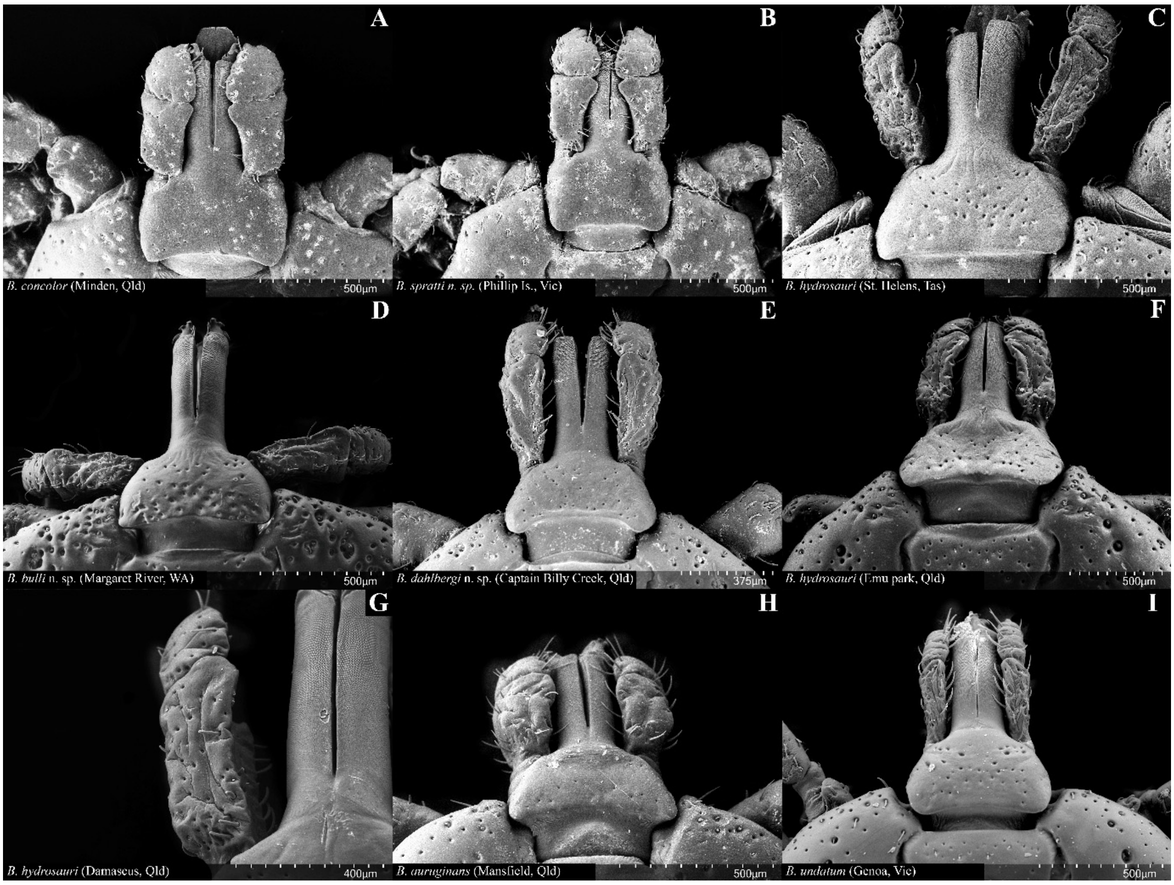

Figure S13. Scanning electron micrographs of the gnathosoma (dorsal view) of males of the nine *Bothriocroton* species ((A) *B. concolor*, Minden, Qld, B3095 ex *Tachyglossus aculeatus*; (B) *B. spratti* n. sp., Phillip Is, Vic, B8141 ex *T. aculeatus*; (C) *B. hydrosauri,* St. Helens, Tas, B8473 ex *Tiliqua nigrolutea*; (D) *B. bulli* n. sp., Young River, WA, B9105 ex *T. rugosa*; (E) *B. dahlbergi* n. sp., Captain Billy Creek, Qld, B3148 ex *T. aculeatus* (Not genotyped); (F) *B. hydrosauri*, Emu Park, Qld, B9104 ex *T. aculeatus*; (G) *B. hydrosauri*, Damascus via Gin Gin, Qld, B8395 ex *T. aculeatus*; (H) *B. auruginans*, Mansfield, Vic, B3349 ex *Vombatus ursinus*; (I) *B. undatum,* Genoa, Vic, B3263 ex *Varanus varius*. The surprising finding of *B. hydrosauri* in Qld led us to study this species by SEM from three localities: (C) St. Helens, Tas; (F) Emu Park, Qld; (G) Damascus via Gin Gin, Qld.

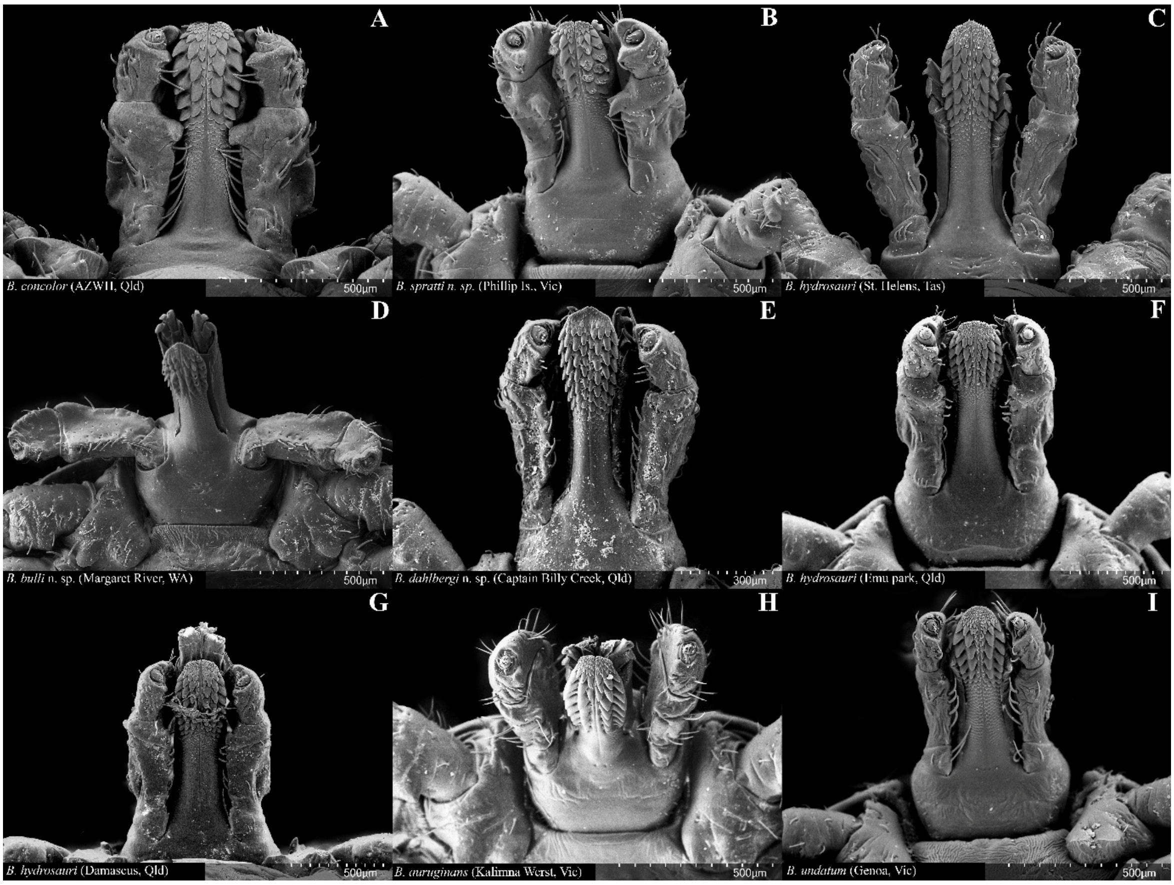

Figure S14. Scanning electron micrographs of the gnathosoma (ventral view) of males of the nine *Bothriocroton* species ((A) *B. concolor*, Australia Zoo Wildlife Hospital, Qld, B8060 ex *Tachyglossus aculeatus*; (B) *B. spratti* n. sp., Phillip Is, Vic, B8141 ex *T. aculeatus*; (C) *B. hydrosauri,* St. Helens, Tas, B8473 ex *Tiliqua nigrolutea*; (D) *B. bulli* n. sp., Young River, WA, B9105 ex *T. rugosa*; (E) *B. dahlbergi* n. sp., Captain Billy Creek, Qld, B3148 ex *T. aculeatus* (Not genotyped); (F) *B. hydrosauri*, Emu Park, Qld, B9104 ex *T. aculeatus*; (G) *B. hydrosauri*, Damascus via Gin Gin, Qld, B8395 ex *T. aculeatus*; (H) *B. auruginans*, Kalimna West, Vic, B3401 ex *Canis familiaris*; (I) *B. undatum,* Genoa, Vic, B3263 ex *Varanus varius*. The surprising finding of *B. hydrosauri* in Qld led us to study this species by SEM from three localities: (C) St. Helens, Tas; (F) Emu Park, Qld; (G) Damascus via Gin Gin, Qld.

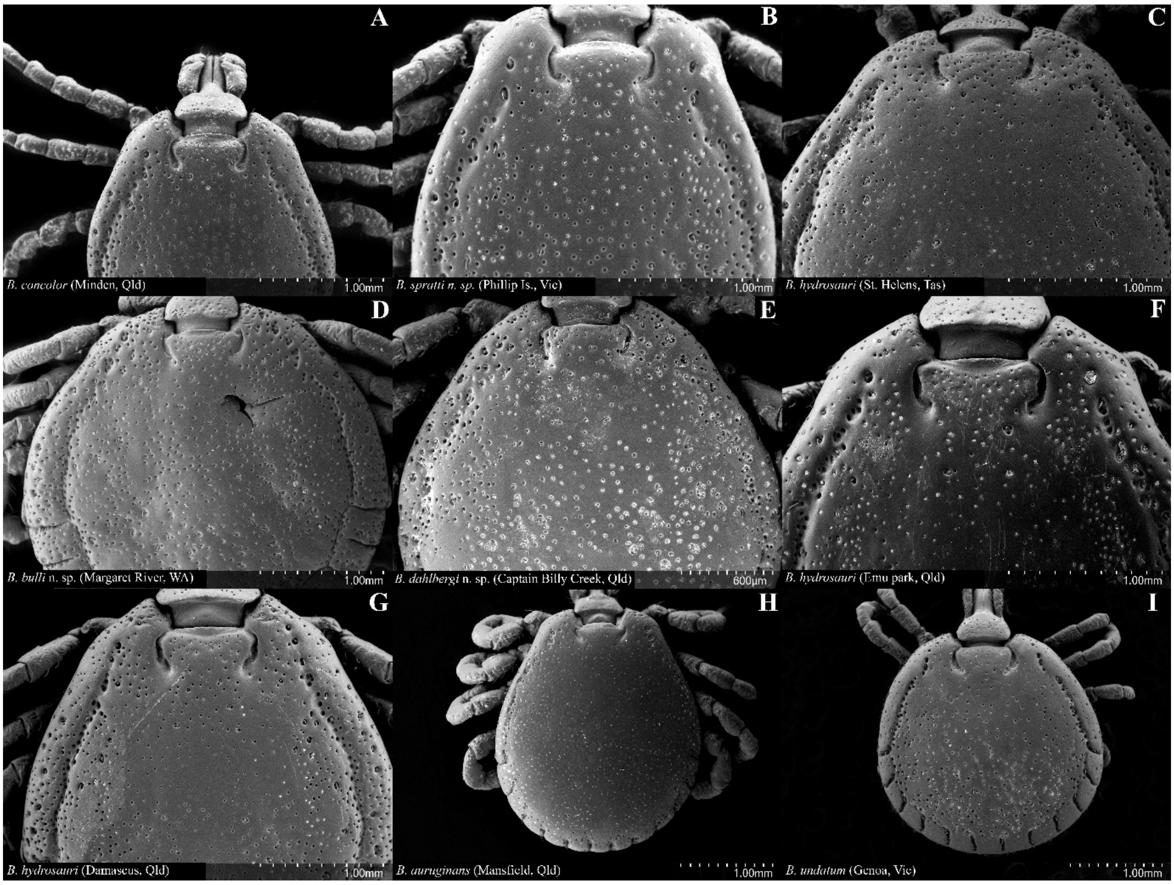

Figure S15. Scanning electron micrographs of the anterior conscutum (dorsal view) of males of the nine *Bothriocroton* species ((A) *B. concolor*, Minden, Qld, B3095 ex *Tachyglossus aculeatus*; (B) *B. spratti* n. sp., Phillip Is, Vic, B8141 ex *T. aculeatus*; (C) *B. hydrosauri,* St. Helens, Tas, B8473 ex *Tiliqua nigrolutea*; (D) *B. bulli* n. sp., Young River, WA, B9105 ex *T. rugosa*; (E) *B. dahlbergi* n. sp., Captain Billy Creek, Qld, B3148 ex *T. aculeatus* (not genotyped); (F) *B. hydrosauri*, Emu Park, Qld, B9104 ex *T. aculeatus*; (G) *B. hydrosauri*, Damascus via Gin Gin, Qld, B8395 ex *T. aculeatus*; (H) *B. auruginans*, Mansfield, Vic, B3349 ex *Vombatus ursinus*; (I) *B. undatum,* Genoa, Vic, B3263 ex *Varanus varius*. The surprising finding of *B. hydrosauri* in Qld led us to study this species by SEM from three localities: (C) St. Helens, Tas; (F) Emu Park, Qld; (G) Damascus via Gin Gin, Qld.

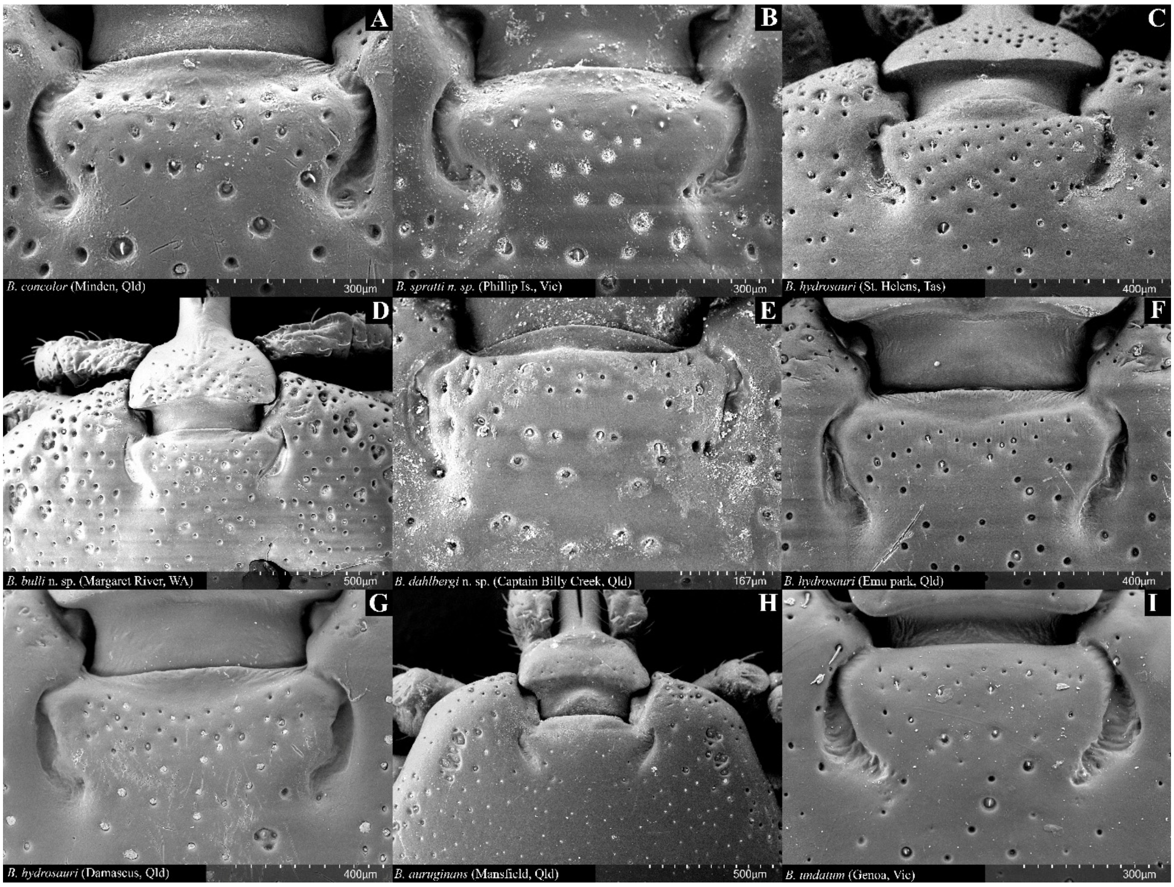

Figure S16. Scanning electron micrographs of the cervical grooves (dorsal view) of males of the nine *Bothriocroton* species ((A) *B. concolor*, Minden, Qld, B3095 ex *Tachyglossus aculeatus*; (B) *B. spratti* n. sp., Phillip Is, Vic, B8141 ex *T. aculeatus*; (C) *B. hydrosauri,* St. Helens, Tas, B8473 ex *Tiliqua nigrolutea*; (D) *B. bulli* n. sp., Young River, WA, B9105 ex *T. rugosa*; (E) *B. dahlbergi* n. sp., Captain Billy Creek, Qld, B3148 ex *T. aculeatus* (Not genotyped); (F) *B. hydrosauri*, Emu Park, Qld, B9104 ex *T. aculeatus*; (G) *B. hydrosauri*, Damascus via Gin Gin, Qld, B8395 ex *T. aculeatus*; (H) *B. auruginans*, Mansfield, Vic, B3349 ex *Vombatus ursinus*; (I) *B. undatum,* Genoa, Vic, B3263 ex *Varanus varius*. The surprising finding of *B. hydrosauri* in Qld led us to study this species by SEM from three localities: (C) St. Helens, Tas; (F) Emu Park, Qld; (G) Damascus via Gin Gin, Qld.

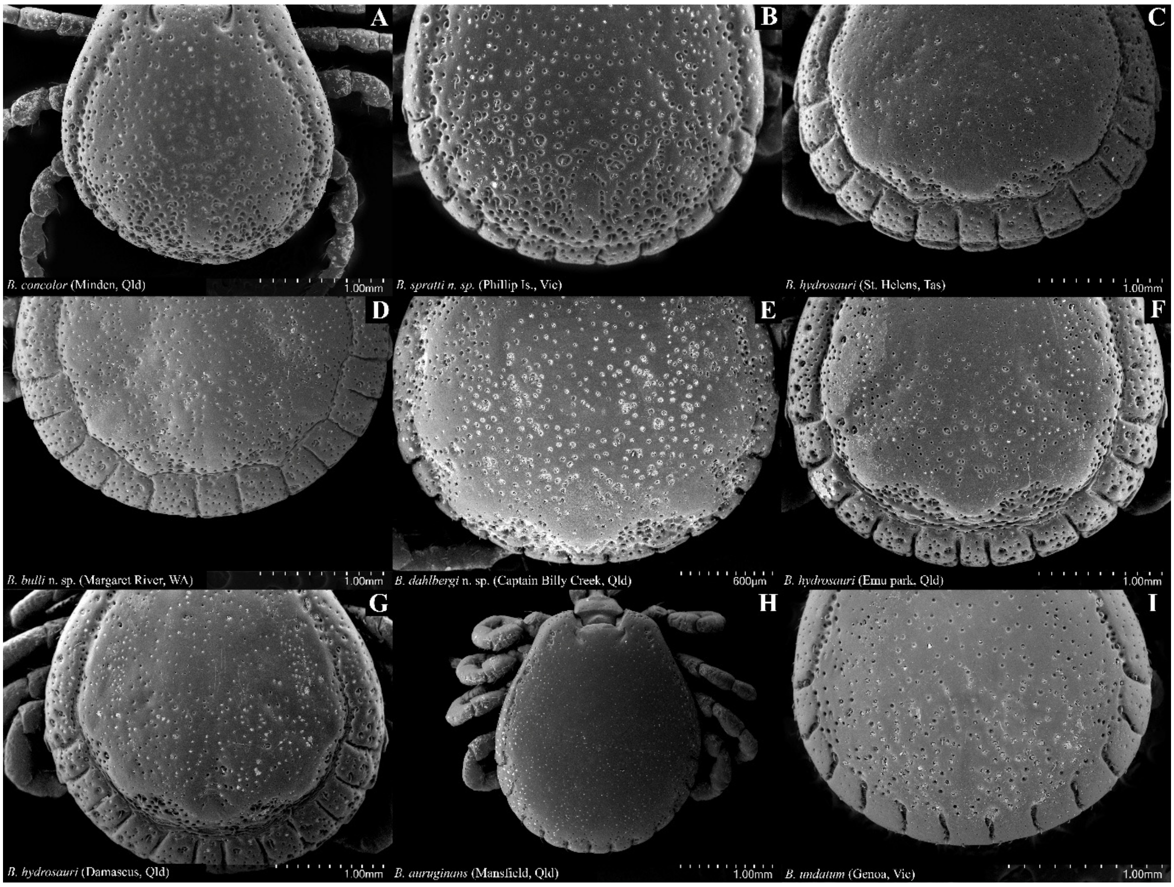

Figure S17. Scanning electron micrographs of the posterior conscutum (dorsal view) of males of the seven *Bothriocroton* species ((A) *B. concolor*, Minden, Qld, B3095 ex *Tachyglossus aculeatus*; (B) *B. spratti* n. sp., Phillip Is, Vic, B8141 ex *T. aculeatus*; (C) *B. hydrosauri,* St. Helens, Tas, B8473 ex *Tiliqua nigrolutea*; (D) *B. bulli* n. sp., Young River, WA, B9105 ex *T. rugosa*; (E) *B. dahlbergi* n. sp., Captain Billy Creek, Qld, B3148 ex *T. aculeatus* (Not genotyped); (F) *B. hydrosauri*, Emu Park, Qld, B9104 ex *T. aculeatus*; (G) *B. hydrosauri*, Damascus via Gin Gin, Qld, B8395 ex *T. aculeatus*; (H) *B. auruginans*, Mansfield, Vic, B3349 ex *Vombatus ursinus*; (I) *B. undatum,* Genoa, Vic, B3263 ex *Varanus varius*. The surprising finding of *B. hydrosauri* in Qld led us to study this species by SEM from three localities: (C) St. Helens, Tas; (F) Emu Park, Qld; (G) Damascus via Gin Gin, Qld.

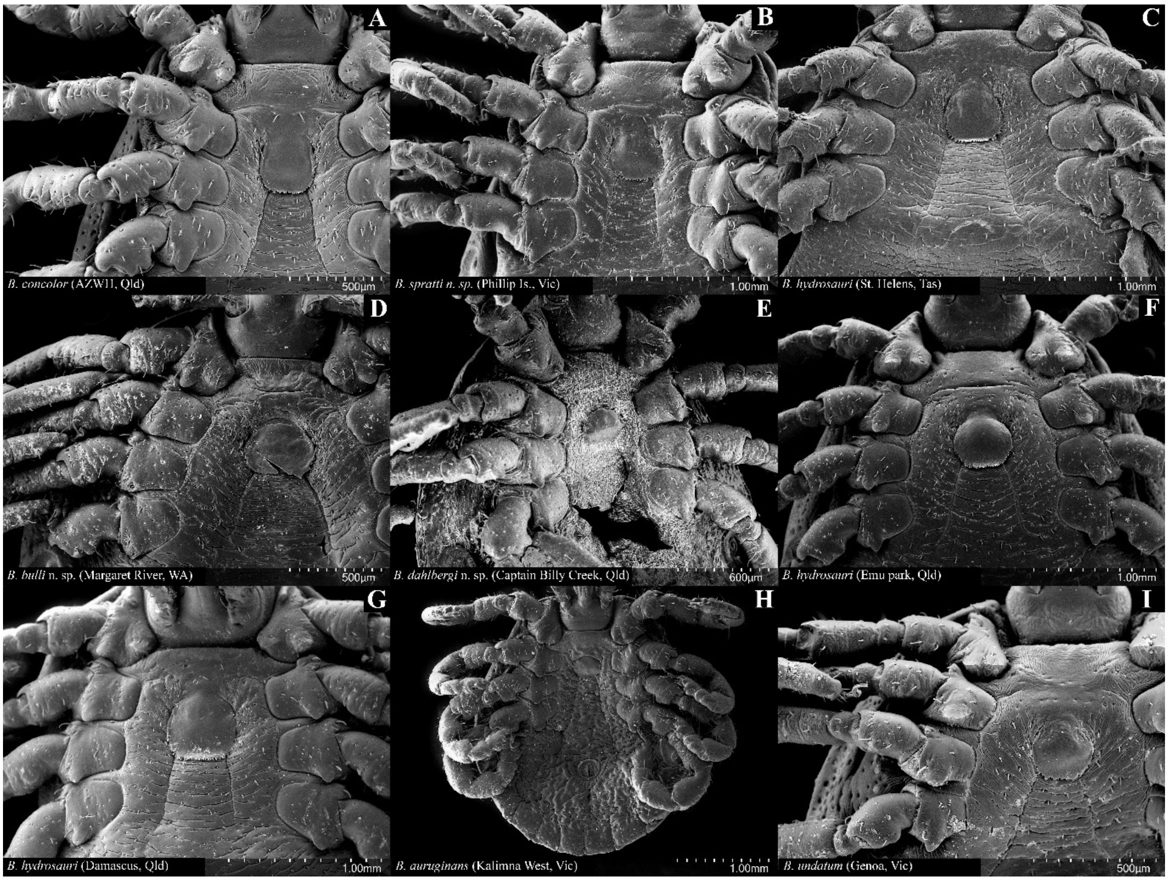

Figure S18. Scanning electron micrographs of the anterior venter of males of the nine *Bothriocroton* species ((A) *B. concolor*, Australia Zoo Wildlife Hospital, Qld, B8060 ex *Tachyglossus aculeatus*; (B) *B. spratti* n. sp., Phillip Is, Vic, B8141 ex *T. aculeatus*; (C) *B. hydrosauri,* St. Helens, Tas, B8473 ex *Tiliqua nigrolutea*; (D) *B. bulli* n. sp., Young River, WA, B9105 ex *T. rugosa*; (E) *B. dahlbergi* n. sp., Captain Billy Creek, Qld, B3148 ex *T. aculeatus* (Not genotyped); (F) *B. hydrosauri*, Emu Park, Qld, B9104 ex *T. aculeatus*; (G) *B. hydrosauri*, Damascus via Gin Gin, Qld, B8395 ex *T. aculeatus*; (H) *B. auruginans*, Kalimna West, Vic, B3401 ex *Canis familiaris*; (I) *B. undatum,* Genoa, Vic, B3263 ex *Varanus varius*. The surprising finding of *B. hydrosauri* in Qld led us to study this species by SEM from three localities: (C) St. Helens, Tas; (F) Emu Park, Qld; (G) Damascus via Gin Gin, Qld.

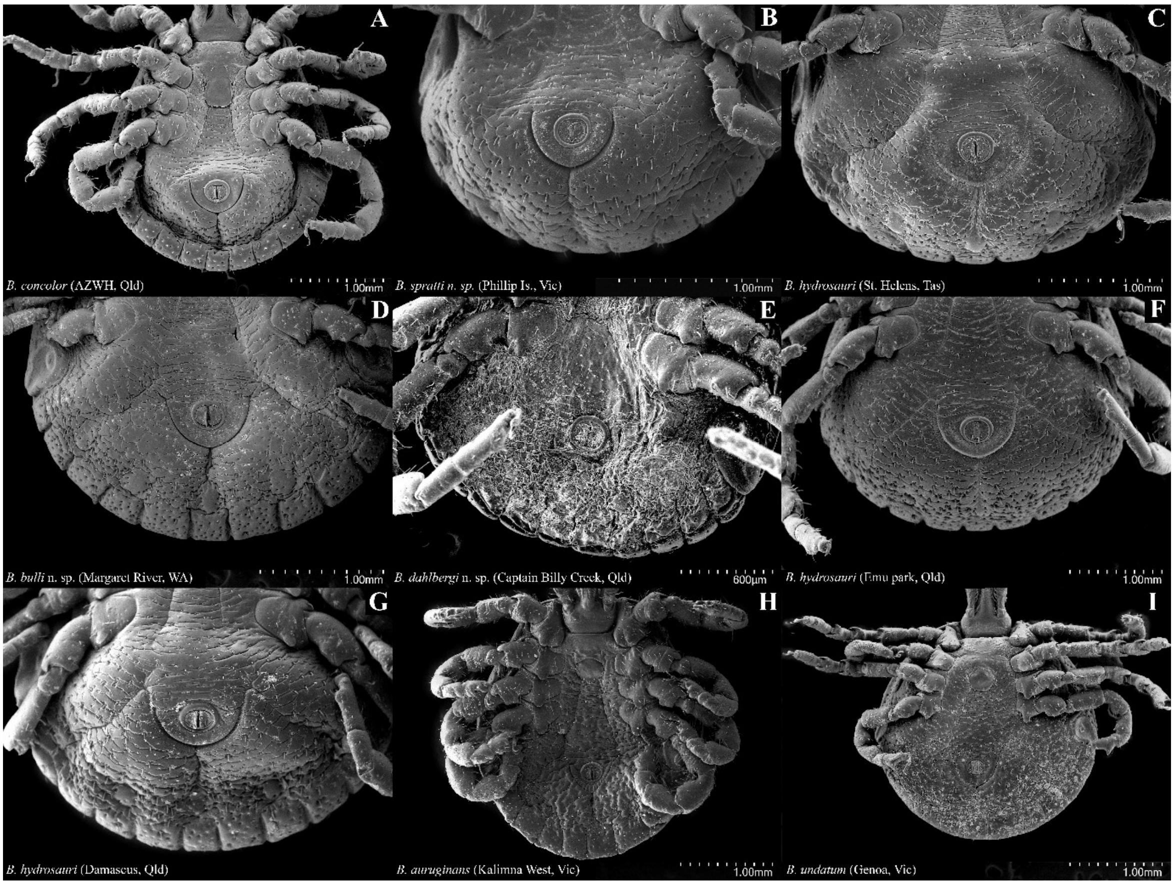

Figure S19. Scanning electron micrographs of the posterior venter of males of the seven *Bothriocroton* species ((A) *B. concolor*, Australia Zoo Wildlife Hospital, Qld, B8060 ex *Tachyglossus aculeatus*; (B) *B. spratti* n. sp., Phillip Is, Vic, B8141 ex *T. aculeatus*; (C) *B. hydrosauri,* St. Helens, Tas, B8473 ex *Tiliqua nigrolutea*; (D) *B. bulli* n. sp., Young River, WA, B9105 ex *T. rugosa*; (E) *B. dahlbergi* n. sp., Captain Billy Creek, Qld, B3148 ex *T. aculeatus* (Not genotyped); (F) *B. hydrosauri*, Emu Park, Qld, B9104 ex *T. aculeatus*; (G) *B. hydrosauri*, Damascus via Gin Gin, Qld, B8395 ex *T. aculeatus*; (H) *B. auruginans*, Kalimna West, Vic, B3401 ex *Canis familiaris*; (I) *B. undatum,* Genoa, Vic, B3263 ex *Varanus varius*. The surprising finding of *B. hydrosauri* in Qld led us to study this species by SEM from three localities: (C) St. Helens, Tas; (F) Emu Park, Qld; (G) Damascus via Gin Gin, Qld.

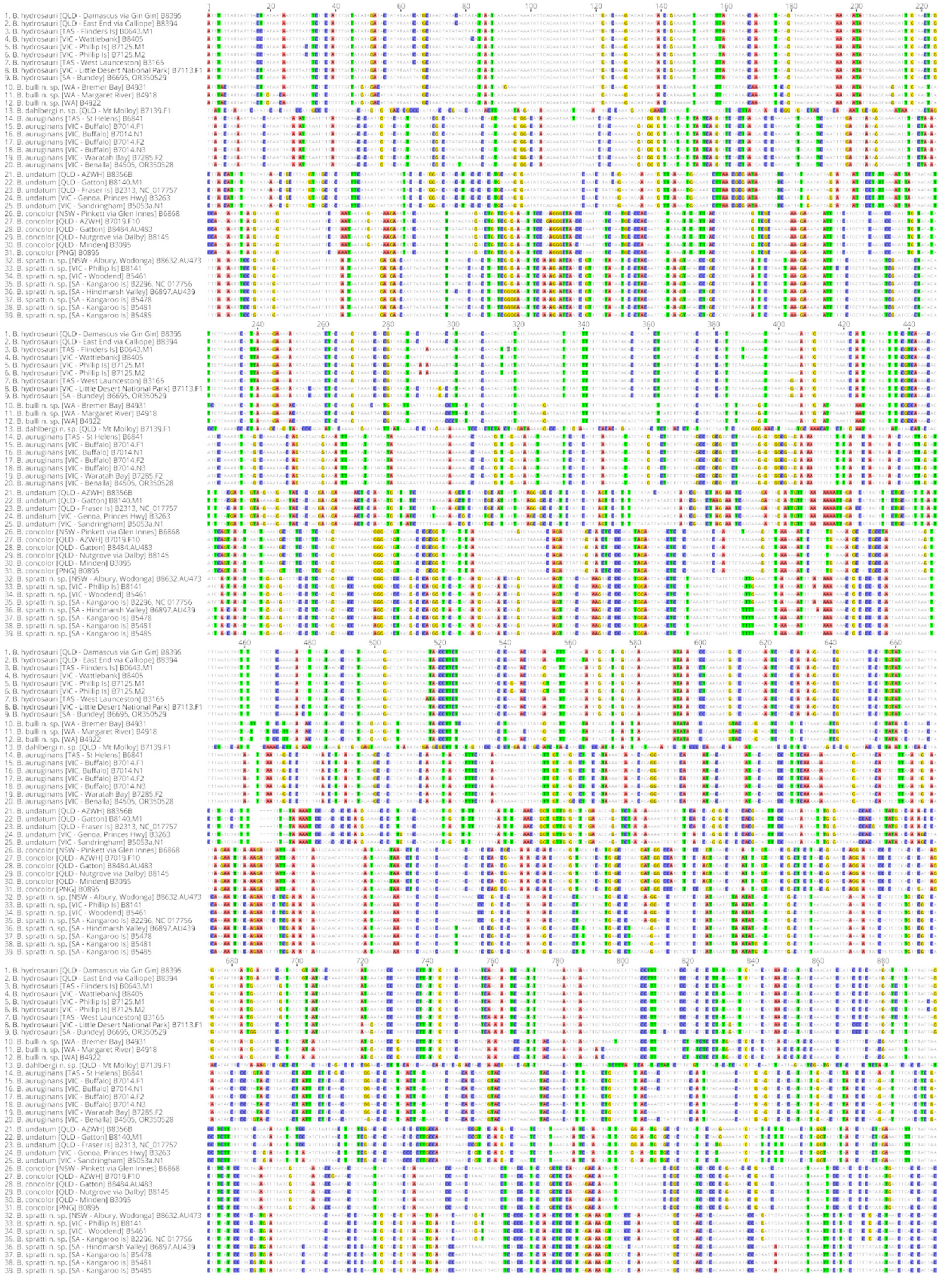
1. B. hydrosauri [QLD - Damascus via Gin Gin] B8395
2. B. hydrosauri [QLD - East End via Calliope] B8394
3. B. hydrosauri [TAS - Flinders Is] B0643.M1
4. B. hydrosauri [VIC - Wattlebank] B8405
5. B. hydrosauri [VIC - Phillip Is] B7125.M1
6. B. hydrosauri [VIC - Phillip Is] B7125.M2
7. B. hydrosauri [TAS - West Launceston] B3165
8. B. hydrosauri [VIC - Little Desert National Park] B7113.F1
9. B. hydrosauri [SA - Bundey] B6695, OR350529
10. B. bulli n. sp. [WA - Bremer Bay] B4931
11. B. bulli n. sp. [WA - Margaret River] B4918
12. B. bulli n. sp. [WA] B4922
13. B. dahlbergi n. sp. [QLD - Mt Molloy] B7139.F1
14. B. auruginans [TAS - St Helens] B6841
15. B. auruginans [VIC - Buffalo] B7014.F1
16. B. auruginans [VIC, Buffalo] B7014.N1
17. B. auruginans [VIC - Buffalo] B7014.F2
18. B. auruginans [VIC - Buffalo] B7014.N3
19. B. auruginans [VIC - Waratah Bay] B7285.F2
20. B. auruginans [VIC - Benalla] B4505, OR350528
21. B. undatum [QLD - AZWH] B8356B
22. B. undatum [QLD - Gatton] B8140.M1
23. B. undatum [QLD - Fraser Is] B2313, NC_017757
24. B. undatum [VIC - Genoa, Princes Hwy] B3263
25. B. undatum [VIC - Sandringham] B5053a.N1
26. B. concolor [NSW - Pinkett via Glen Innes] B6868
27. B. concolor [QLD - AZWH] B7019.F10
28. B. concolor [QLD - Gatton] B8484.AU483
29. B. concolor [QLD - Nutgrove via Dalby] B8145
30. B. concolor [QLD - Minden] B3095
31. B. concolor [PNG] B0895
32. B. spratti n. sp. [NSW - Albury, Wodonga] B8632.AU473
33. B. spratti n. sp. [VIC - Phillip Is] B8141
34. B. spratti n. sp. [VIC - Woodend] B5461
35. B. spratti n. sp. [SA - Kangaroo Is] B2296, NC 017756
36. B. spratti n. sp. [SA - Hindmarsh Valley] B6897.AU439
37. B. spratti n. sp. [SA - Kangaroo Is] B5478
38. B. spratti n. sp. [SA - Kangaroo Is] B5481
39. B. spratti n. sp. [SA - Kangaroo Is] B5485

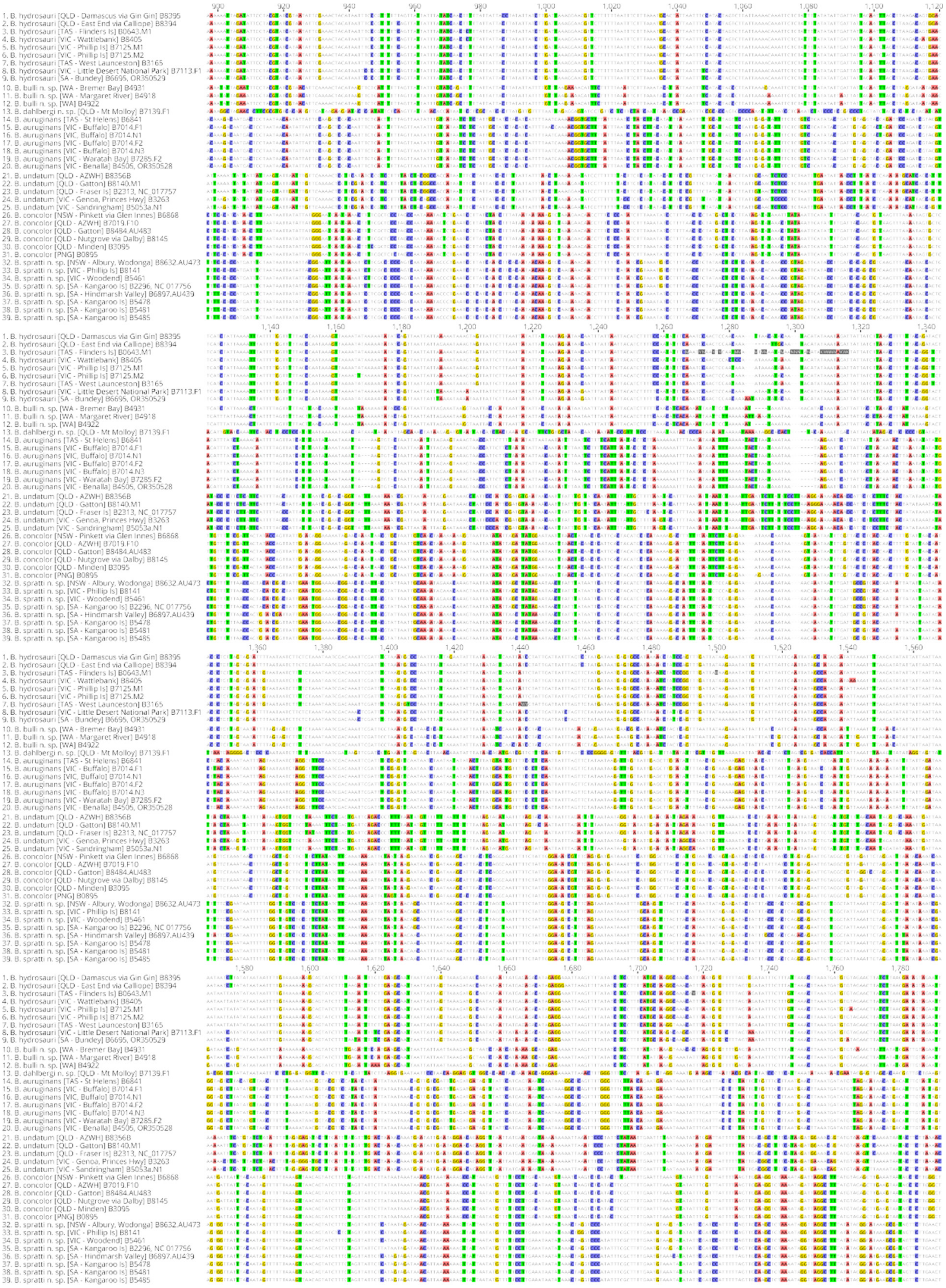
900
920
940
960
980
1,000
1,020
1,040
1,060
1,080
1,100
1,120
1. B. hydrosauri [QLD - Damascus via Gin Gin] B8395
2. B. hydrosauri [QLD - East End via Calliope] B8394
3. B. hydrosauri [TAS - Flinders Is] B0643.M1
4. B. hydrosauri [VIC - Wattlebank] B8405
5. B. hydrosauri [VIC - Phillip Is] B7125.M1
6. B. hydrosauri [VIC - Phillip Is] B7125.M2
7. B. hydrosauri [TAS - West Launceston] B3165
8. B. hydrosauri [VIC - Little Desert National Park] B7113.F1
9. B. hydrosauri [SA - Bundey] B6695, OR350529
10. B. bulli n. sp. [WA - Bremer Bay] B4931
11. B. bulli n. sp. [WA - Margaret River] B4918
12. B. bulli n. sp. [WA] B4922
13. B. dahlbergi n. sp. [QLD - Mt Molloy] B7139.F1
14. B. auruginans [TAS - St Helens] B6841
15. B. auruginans [VIC - Buffalo] B7014.F1
16. B. auruginans [VIC, Buffalo] B7014.N1
17. B. auruginans [VIC - Buffalo] B7014.F2
18. B. auruginans [VIC - Buffalo] B7014.N3
19. B. auruginans [VIC - Waratah Bay] B7285.F2
20. B. auruginans [VIC - Benalla] B4505, OR350528
21. B. undatum [QLD - AZWH] B8356B
22. B. undatum [QLD - Gatton] B8140.M1
23. B. undatum [QLD - Fraser Is] B2313, NC_017757
24. B. undatum [VIC - Genoa, Princes Hwy] B3263
25. B. undatum [VIC - Sandringham] B5053a.N1
26. B. concolor [NSW - Pinkett via Glen Innes] B6868
27. B. concolor [QLD - AZWH] B7019.F10
28. B. concolor [QLD - Gatton] B8484.AU483
29. B. concolor [QLD - Nutgrove via Dalby] B8145
30. B. concolor [QLD - Minden] B3095
31. B. concolor [PNG] B0895
32. B. spratti n. sp. [NSW - Albury, Wodonga] B8632.AU473
33. B. spratti n. sp. [VIC - Phillip Is] B8141
34. B. spratti n. sp. [VIC - Woodend] B5461
35. B. spratti n. sp. [SA - Kangaroo Is] B2296, NC 017756
36. B. spratti n. sp. [SA - Hindmarsh Valley] B6897.AU439
37. B. spratti n. sp. [SA - Kangaroo Is] B5478
38. B. spratti n. sp. [SA - Kangaroo Is] B5481
39. B. spratti n. sp. [SA - Kangaroo Is] B5485
1,140
1,160
1,180
1,200
1,220
1,240
1,260
1,280
1,300
1,320
1,340
1. B. hydrosauri [QLD - Damascus via Gin Gin] B8395
2. B. hydrosauri [QLD - East End via Calliope] B8394
3. B. hydrosauri [TAS - Flinders Is] B0643.M1
4. B. hydrosauri [VIC - Wattlebank] B8405
5. B. hydrosauri [VIC - Phillip Is] B7125.M1
6. B. hydrosauri [VIC - Phillip Is] B7125.M2
7. B. hydrosauri [TAS - West Launceston] B3165
8. B. hydrosauri [VIC - Little Desert National Park] B7113.F1
9. B. hydrosauri [SA - Bundey] B6695, OR350529
10. B. bulli n. sp. [WA - Bremer Bay] B4931
11. B. bulli n. sp. [WA - Margaret River] B4918
12. B. bulli n. sp. [WA] B4922
13. B. dahlbergi n. sp. [QLD - Mt Molloy] B7139.F1
14. B. auruginans [TAS - St Helens] B6841
15. B. auruginans [VIC - Buffalo] B7014.F1
16. B. auruginans [VIC, Buffalo] B7014.N1
17. B. auruginans [VIC - Buffalo] B7014.F2
18. B. auruginans [VIC - Buffalo] B7014.N3
19. B. auruginans [VIC - Waratah Bay] B7285.F2
20. B. auruginans [VIC - Benalla] B4505, OR350528
21. B. undatum [QLD - AZWH] B8356B
22. B. undatum [QLD - Gatton] B8140.M1
23. B. undatum [QLD - Fraser Is] B2313, NC_017757
24. B. undatum [VIC - Genoa, Princes Hwy] B3263
25. B. undatum [VIC - Sandringham] B5053a.N1
26. B. concolor [NSW - Pinkett via Glen Innes] B6868
27. B. concolor [QLD - AZWH] B7019.F10
28. B. concolor [QLD - Gatton] B8484.AU483
29. B. concolor [QLD - Nutgrove via Dalby] B8145
30. B. concolor [QLD - Minden] B3095
31. B. concolor [PNG] B0895
32. B. spratti n. sp. [NSW - Albury, Wodonga] B8632.AU473
33. B. spratti n. sp. [VIC - Phillip Is] B8141
34. B. spratti n. sp. [VIC - Woodend] B5461
35. B. spratti n. sp. [SA - Kangaroo Is] B2296, NC 017756
36. B. spratti n. sp. [SA - Hindmarsh Valley] B6897.AU439
37. B. spratti n. sp. [SA - Kangaroo Is] B5478
38. B. spratti n. sp. [SA - Kangaroo Is] B5481
39. B. spratti n. sp. [SA - Kangaroo Is] B5485
1,360
1,380
1,400
1,420
1,440
1,460
1,480
1,500
1,520
1,540
1,560
1. B. hydrosauri [QLD - Damascus via Gin Gin] B8395
2. B. hydrosauri [QLD - East End via Calliope] B8394
3. B. hydrosauri [TAS - Flinders Is] B0643.M1
4. B. hydrosauri [VIC - Wattlebank] B8405
5. B. hydrosauri [VIC - Phillip Is] B7125.M1
6. B. hydrosauri [VIC - Phillip Is] B7125.M2
7. B. hydrosauri [TAS - West Launceston] B3165
8. B. hydrosauri [VIC - Little Desert National Park] B7113.F1
9. B. hydrosauri [SA - Bundey] B6695, OR350529
10. B. bulli n. sp. [WA - Bremer Bay] B4931
11. B. bulli n. sp. [WA - Margaret River] B4918
12. B. bulli n. sp. [WA] B4922
13. B. dahlbergi n. sp. [QLD - Mt Molloy] B7139.F1
14. B. auruginans [TAS - St Helens] B6841
15. B. auruginans [VIC - Buffalo] B7014.F1
16. B. auruginans [VIC, Buffalo] B7014.N1
17. B. auruginans [VIC - Buffalo] B7014.F2
18. B. auruginans [VIC - Buffalo] B7014.N3
19. B. auruginans [VIC - Waratah Bay] B7285.F2
20. B. auruginans [VIC - Benalla] B4505, OR350528
21. B. undatum [QLD - AZWH] B8356B
22. B. undatum [QLD - Gatton] B8140.M1
23. B. undatum [QLD - Fraser Is] B2313, NC_017757
24. B. undatum [VIC - Genoa, Princes Hwy] B3263
25. B. undatum [VIC - Sandringham] B5053a.N1
26. B. concolor [NSW - Pinkett via Glen Innes] B6868
27. B. concolor [QLD - AZWH] B7019.F10
28. B. concolor [QLD - Gatton] B8484.AU483
29. B. concolor [QLD - Nutgrove via Dalby] B8145
30. B. concolor [QLD - Minden] B3095
31. B. concolor [PNG] B0895
32. B. spratti n. sp. [NSW - Albury, Wodonga] B8632.AU473
33. B. spratti n. sp. [VIC - Phillip Is] B8141
34. B. spratti n. sp. [VIC - Woodend] B5461
35. B. spratti n. sp. [SA - Kangaroo Is] B2296, NC 017756
36. B. spratti n. sp. [SA - Hindmarsh Valley] B6897.AU439
37. B. spratti n. sp. [SA - Kangaroo Is] B5478
38. B. spratti n. sp. [SA - Kangaroo Is] B5481
39. B. spratti n. sp. [SA - Kangaroo Is] B5485
1,580
1,600
1,620
1,640
1,660
1,680
1,700
1,720
1,740
1,760
1,780
1. B. hydrosauri [QLD - Damascus via Gin Gin] B8395
2. B. hydrosauri [QLD - East End via Calliope] B8394
3. B. hydrosauri [TAS - Flinders Is] B0643.M1
4. B. hydrosauri [VIC - Wattlebank] B8405
5. B. hydrosauri [VIC - Phillip Is] B7125.M1
6. B. hydrosauri [VIC - Phillip Is] B7125.M2
7. B. hydrosauri [TAS - West Launceston] B3165
8. B. hydrosauri [VIC - Little Desert National Park] B7113.F1
9. B. hydrosauri [SA - Bundey] B6695, OR350529
10. B. bulli n. sp. [WA - Bremer Bay] B4931
11. B. bulli n. sp. [WA - Margaret River] B4918
12. B. bulli n. sp. [WA] B4922
13. B. dahlbergi n. sp. [QLD - Mt Molloy] B7139.F1
14. B. auruginans [TAS - St Helens] B6841
15. B. auruginans [VIC - Buffalo] B7014.F1
16. B. auruginans [VIC, Buffalo] B7014.N1
17. B. auruginans [VIC - Buffalo] B7014.F2
18. B. auruginans [VIC - Buffalo] B7014.N3
19. B. auruginans [VIC - Waratah Bay] B7285.F2
20. B. auruginans [VIC - Benalla] B4505, OR350528
21. B. undatum [QLD - AZWH] B8356B
22. B. undatum [QLD - Gatton] B8140.M1
23. B. undatum [QLD - Fraser Is] B2313, NC_017757
24. B. undatum [VIC - Genoa, Princes Hwy] B3263
25. B. undatum [VIC - Sandringham] B5053a.N1
26. B. concolor [NSW - Pinkett via Glen Innes] B6868
27. B. concolor [QLD - AZWH] B7019.F10
28. B. concolor [QLD - Gatton] B8484.AU483
29. B. concolor [QLD - Nutgrove via Dalby] B8145
30. B. concolor [QLD - Minden] B3095
31. B. concolor [PNG] B0895
32. B. spratti n. sp. [NSW - Albury, Wodonga] B8632.AU473
33. B. spratti n. sp. [VIC - Phillip Is] B8141
34. B. spratti n. sp. [VIC - Woodend] B5461
35. B. spratti n. sp. [SA - Kangaroo Is] B2296, NC 017756
36. B. spratti n. sp. [SA - Hindmarsh Valley] B6897.AU439
37. B. spratti n. sp. [SA - Kangaroo Is] B5478
38. B. spratti n. sp. [SA - Kangaroo Is] B5481
39. B. spratti n. sp. [SA - Kangaroo Is] B5485

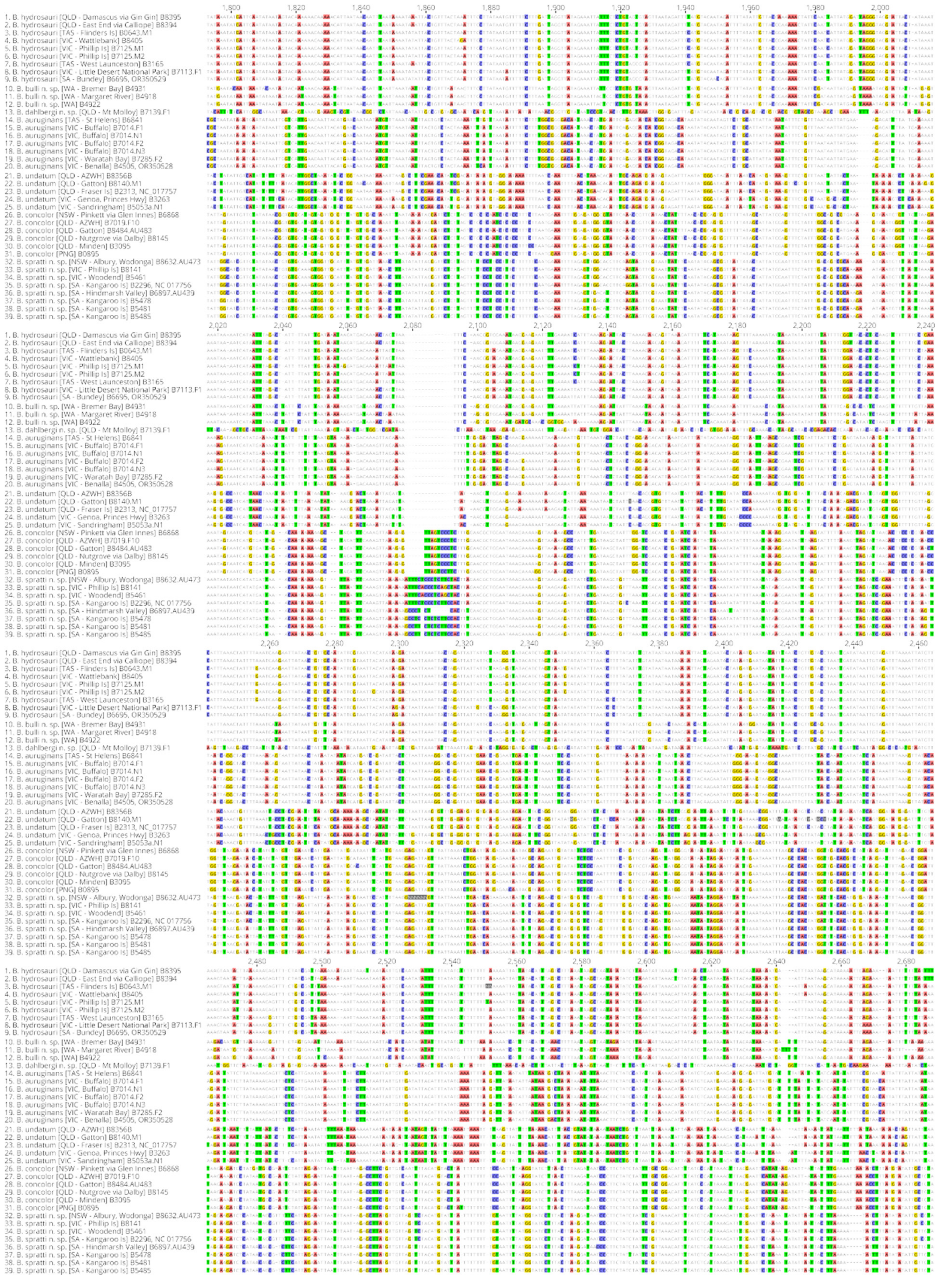

1,800
1,820
1,840
1,860
1,880
1,900
1,920
1,940
1,960
1,980
2,000
1. B. hydrosauri [QLD - Damascus via Gin Gin] B8395
2. B. hydrosauri [QLD - East End via Calliope] B8394
3. B. hydrosauri [TAS - Flinders Is] B0643.M1
4. B. hydrosauri [VIC - Wattlebank] B8405
5. B. hydrosauri [VIC - Phillip Is] B7125.M1
6. B. hydrosauri [VIC - Phillip Is] B7125.M2
7. B. hydrosauri [TAS - West Launceston] B3165
8. B. hydrosauri [VIC - Little Desert National Park] B7113.F1
9. B. hydrosauri [SA - Bundey] B6695, OR350529
10. B. bulli n. sp. [WA - Bremer Bay] B4931
11. B. bulli n. sp. [WA - Margaret River] B4918
12. B. bulli n. sp. [WA] B4922
13. B. dahlbergi n. sp. [QLD - Mt Molloy] B7139.F1
14. B. auruginans [TAS - St Helens] B6841
15. B. auruginans [VIC - Buffalo] B7014.F1
16. B. auruginans [VIC, Buffalo] B7014.N1
17. B. auruginans [VIC - Buffalo] B7014.F2
18. B. auruginans [VIC - Buffalo] B7014.N3
19. B. auruginans [VIC - Waratah Bay] B7285.F2
20. B. auruginans [VIC - Benalla] B4505, OR350528
21. B. undatum [QLD - AZWH] B8356B
22. B. undatum [QLD - Gatton] B8140.M1
23. B. undatum [QLD - Fraser Is] B2313, NC_017757
24. B. undatum [VIC - Genoa, Princes Hwy] B3263
25. B. undatum [VIC - Sandringham] B5053a.N1
26. B. concolor [NSW - Pinkett via Glen Innes] B6868
27. B. concolor [QLD - AZWH] B7019.F10
28. B. concolor [QLD - Gatton] B8484.AU483
29. B. concolor [QLD - Nutgrove via Dalby] B8145
30. B. concolor [QLD - Minden] B3095
31. B. concolor [PNG] B0895
32. B. spratti n. sp. [NSW - Albury, Wodonga] B8632.AU473
33. B. spratti n. sp. [VIC - Phillip Is] B8141
34. B. spratti n. sp. [VIC - Woodend] B5461
35. B. spratti n. sp. [SA - Kangaroo Is] B2296, NC 017756
36. B. spratti n. sp. [SA - Hindmarsh Valley] B6897.AU439
37. B. spratti n. sp. [SA - Kangaroo Is] B5478
38. B. spratti n. sp. [SA - Kangaroo Is] B5481
39. B. spratti n. sp. [SA - Kangaroo Is] B5485
2,020
2,040
2,060
2,080
2,100
2,120
2,140
2,160
2,180
2,200
2,220
2,240
2,260
2,280
2,300
2,320
2,340
2,360
2,380
2,400
2,420
2,440
2,460
2,480
2,500
2,520
2,540
2,560
2,580
2,600
2,620
2,640
2,660
2,680

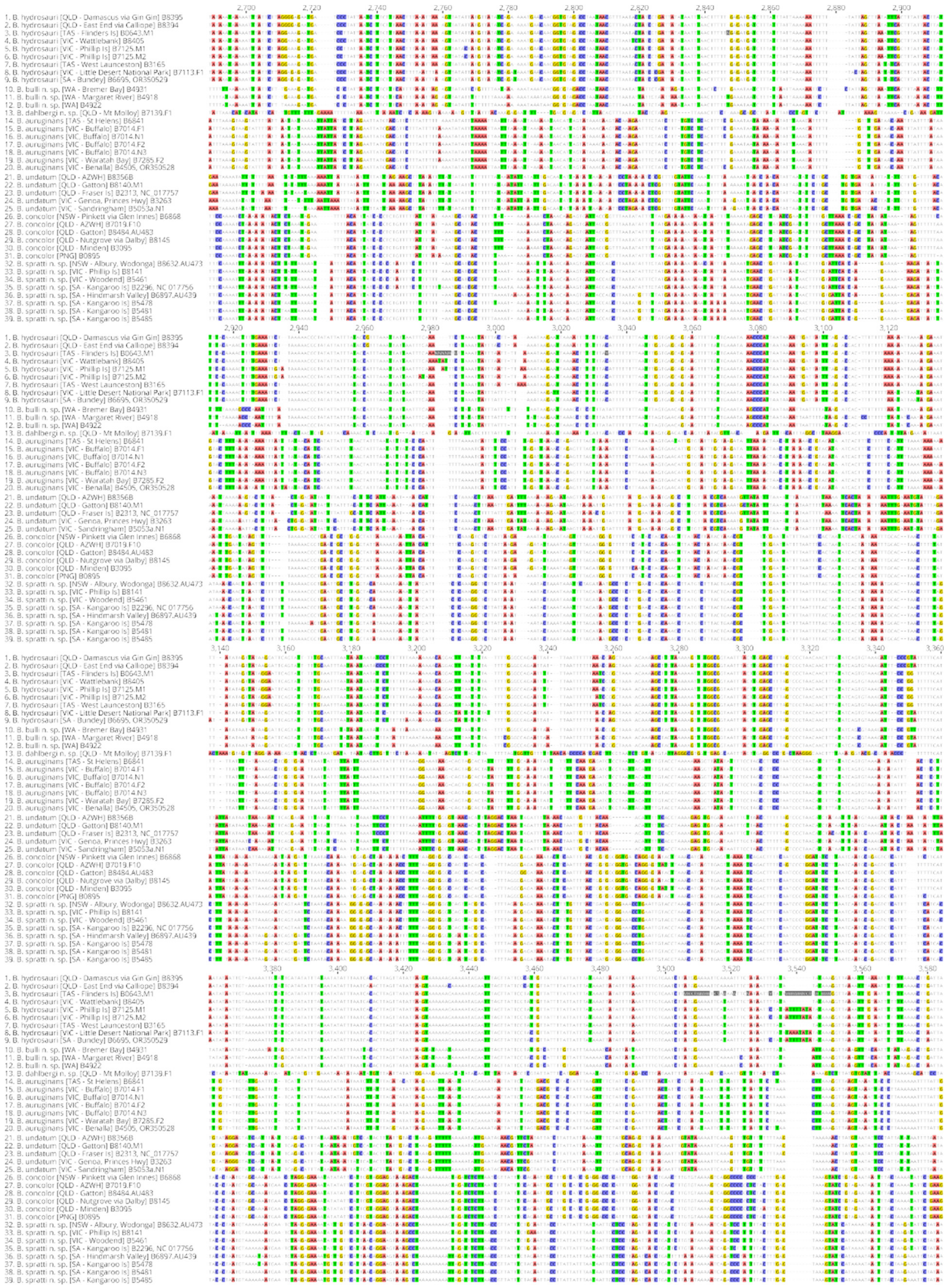
2,700
2,720
2,740
2,760
2,780
2,800
2,820
2,840
2,860
2,880
2,900
1. B. hydrosauri [QLD - Damascus via Gin Gin] B8395
2. B. hydrosauri [QLD - East End via Calliope] B8394
3. B. hydrosauri [TAS - Flinders Is] B0643.M1
4. B. hydrosauri [VIC - Wattlebank] B8405
5. B. hydrosauri [VIC - Phillip Is] B7125.M1
6. B. hydrosauri [VIC - Phillip Is] B7125.M2
7. B. hydrosauri [TAS - West Launceston] B3165
8. B. hydrosauri [VIC - Little Desert National Park] B7113.F1
9. B. hydrosauri [SA - Bundey] B6695, OR350529
10. B. bulli n. sp. [WA - Bremer Bay] B4931
11. B. bulli n. sp. [WA - Margaret River] B4918
12. B. bulli n. sp. [WA] B4922
13. B. dahlbergi n. sp. [QLD - Mt Molloy] B7139.F1
14. B. auruginans [TAS - St Helens] B6841
15. B. auruginans [VIC - Buffalo] B7014.F1
16. B. auruginans [VIC, Buffalo] B7014.N1
17. B. auruginans [VIC - Buffalo] B7014.F2
18. B. auruginans [VIC - Buffalo] B7014.N3
19. B. auruginans [VIC - Waratah Bay] B7285.F2
20. B. auruginans [VIC - Benalla] B4505, OR350528
21. B. undatum [QLD - AZWH] B8356B
22. B. undatum [QLD - Gatton] B8140.M1
23. B. undatum [QLD - Fraser Is] B2313, NC_017757
24. B. undatum [VIC - Genoa, Princes Hwy] B3263
25. B. undatum [VIC - Sandringham] B5053a.N1
26. B. concolor [NSW - Pinkett via Glen Innes] B6868
27. B. concolor [QLD - AZWH] B7019.F10
28. B. concolor [QLD - Gatton] B8484.AU483
29. B. concolor [QLD - Nutgrove via Dalby] B8145
30. B. concolor [QLD - Minden] B3095
31. B. concolor [PNG] B0895
32. B. spratti n. sp. [NSW - Albury, Wodonga] B8632.AU473
33. B. spratti n. sp. [VIC - Phillip Is] B8141
34. B. spratti n. sp. [VIC - Woodend] B5461
35. B. spratti n. sp. [SA - Kangaroo Is] B2296, NC 017756
36. B. spratti n. sp. [SA - Hindmarsh Valley] B6897.AU439
37. B. spratti n. sp. [SA - Kangaroo Is] B5478
38. B. spratti n. sp. [SA - Kangaroo Is] B5481
39. B. spratti n. sp. [SA - Kangaroo Is] B5485
2,920
2,940
2,960
2,980
3,000
3,020
3,040
3,060
3,080
3,100
3,120
1. B. hydrosauri [QLD - Damascus via Gin Gin] B8395
2. B. hydrosauri [QLD - East End via Calliope] B8394
3. B. hydrosauri [TAS - Flinders Is] B0643.M1
4. B. hydrosauri [VIC - Wattlebank] B8405
5. B. hydrosauri [VIC - Phillip Is] B7125.M1
6. B. hydrosauri [VIC - Phillip Is] B7125.M2
7. B. hydrosauri [TAS - West Launceston] B3165
8. B. hydrosauri [VIC - Little Desert National Park] B7113.F1
9. B. hydrosauri [SA - Bundey] B6695, OR350529
10. B. bulli n. sp. [WA - Bremer Bay] B4931
11. B. bulli n. sp. [WA - Margaret River] B4918
12. B. bulli n. sp. [WA] B4922
13. B. dahlbergi n. sp. [QLD - Mt Molloy] B7139.F1
14. B. auruginans [TAS - St Helens] B6841
15. B. auruginans [VIC - Buffalo] B7014.F1
16. B. auruginans [VIC, Buffalo] B7014.N1
17. B. auruginans [VIC - Buffalo] B7014.F2
18. B. auruginans [VIC - Buffalo] B7014.N3
19. B. auruginans [VIC - Waratah Bay] B7285.F2
20. B. auruginans [VIC - Benalla] B4505, OR350528
21. B. undatum [QLD - AZWH] B8356B
22. B. undatum [QLD - Gatton] B8140.M1
23. B. undatum [QLD - Fraser Is] B2313, NC_017757
24. B. undatum [VIC - Genoa, Princes Hwy] B3263
25. B. undatum [VIC - Sandringham] B5053a.N1
26. B. concolor [NSW - Pinkett via Glen Innes] B6868
27. B. concolor [QLD - AZWH] B7019.F10
28. B. concolor [QLD - Gatton] B8484.AU483
29. B. concolor [QLD - Nutgrove via Dalby] B8145
30. B. concolor [QLD - Minden] B3095
31. B. concolor [PNG] B0895
32. B. spratti n. sp. [NSW - Albury, Wodonga] B8632.AU473
33. B. spratti n. sp. [VIC - Phillip Is] B8141
34. B. spratti n. sp. [VIC - Woodend] B5461
35. B. spratti n. sp. [SA - Kangaroo Is] B2296, NC 017756
36. B. spratti n. sp. [SA - Hindmarsh Valley] B6897.AU439
37. B. spratti n. sp. [SA - Kangaroo Is] B5478
38. B. spratti n. sp. [SA - Kangaroo Is] B5481
39. B. spratti n. sp. [SA - Kangaroo Is] B5485
3,140
3,160
3,180
3,200
3,220
3,240
3,260
3,280
3,300
3,320
3,340
3,360
1. B. hydrosauri [QLD - Damascus via Gin Gin] B8395
2. B. hydrosauri [QLD - East End via Calliope] B8394
3. B. hydrosauri [TAS - Flinders Is] B0643.M1
4. B. hydrosauri [VIC - Wattlebank] B8405
5. B. hydrosauri [VIC - Phillip Is] B7125.M1
6. B. hydrosauri [VIC - Phillip Is] B7125.M2
7. B. hydrosauri [TAS - West Launceston] B3165
8. B. hydrosauri [VIC - Little Desert National Park] B7113.F1
9. B. hydrosauri [SA - Bundey] B6695, OR350529
10. B. bulli n. sp. [WA - Bremer Bay] B4931
11. B. bulli n. sp. [WA - Margaret River] B4918
12. B. bulli n. sp. [WA] B4922
13. B. dahlbergi n. sp. [QLD - Mt Molloy] B7139.F1
14. B. auruginans [TAS - St Helens] B6841
15. B. auruginans [VIC - Buffalo] B7014.F1
16. B. auruginans [VIC, Buffalo] B7014.N1
17. B. auruginans [VIC - Buffalo] B7014.F2
18. B. auruginans [VIC - Buffalo] B7014.N3
19. B. auruginans [VIC - Waratah Bay] B7285.F2
20. B. auruginans [VIC - Benalla] B4505, OR350528
21. B. undatum [QLD - AZWH] B8356B
22. B. undatum [QLD - Gatton] B8140.M1
23. B. undatum [QLD - Fraser Is] B2313, NC_017757
24. B. undatum [VIC - Genoa, Princes Hwy] B3263
25. B. undatum [VIC - Sandringham] B5053a.N1
26. B. concolor [NSW - Pinkett via Glen Innes] B6868
27. B. concolor [QLD - AZWH] B7019.F10
28. B. concolor [QLD - Gatton] B8484.AU483
29. B. concolor [QLD - Nutgrove via Dalby] B8145
30. B. concolor [QLD - Minden] B3095
31. B. concolor [PNG] B0895
32. B. spratti n. sp. [NSW - Albury, Wodonga] B8632.AU473
33. B. spratti n. sp. [VIC - Phillip Is] B8141
34. B. spratti n. sp. [VIC - Woodend] B5461
35. B. spratti n. sp. [SA - Kangaroo Is] B2296, NC 017756
36. B. spratti n. sp. [SA - Hindmarsh Valley] B6897.AU439
37. B. spratti n. sp. [SA - Kangaroo Is] B5478
38. B. spratti n. sp. [SA - Kangaroo Is] B5481
39. B. spratti n. sp. [SA - Kangaroo Is] B5485
3,380
3,400
3,420
3,440
3,460
3,480
3,500
3,520
3,540
3,560
3,580
1. B. hydrosauri [QLD - Damascus via Gin Gin] B8395
2. B. hydrosauri [QLD - East End via Calliope] B8394
3. B. hydrosauri [TAS - Flinders Is] B0643.M1
4. B. hydrosauri [VIC - Wattlebank] B8405
5. B. hydrosauri [VIC - Phillip Is] B7125.M1
6. B. hydrosauri [VIC - Phillip Is] B7125.M2
7. B. hydrosauri [TAS - West Launceston] B3165
8. B. hydrosauri [VIC - Little Desert National Park] B7113.F1
9. B. hydrosauri [SA - Bundey] B6695, OR350529
10. B. bulli n. sp. [WA - Bremer Bay] B4931
11. B. bulli n. sp. [WA - Margaret River] B4918
12. B. bulli n. sp. [WA] B4922
13. B. dahlbergi n. sp. [QLD - Mt Molloy] B7139.F1
14. B. auruginans [TAS - St Helens] B6841
15. B. auruginans [VIC - Buffalo] B7014.F1
16. B. auruginans [VIC, Buffalo] B7014.N1
17. B. auruginans [VIC - Buffalo] B7014.F2
18. B. auruginans [VIC - Buffalo] B7014.N3
19. B. auruginans [VIC - Waratah Bay] B7285.F2
20. B. auruginans [VIC - Benalla] B4505, OR350528
21. B. undatum [QLD - AZWH] B8356B
22. B. undatum [QLD - Gatton] B8140.M1
23. B. undatum [QLD - Fraser Is] B2313, NC_017757
24. B. undatum [VIC - Genoa, Princes Hwy] B3263
25. B. undatum [VIC - Sandringham] B5053a.N1
26. B. concolor [NSW - Pinkett via Glen Innes] B6868
27. B. concolor [QLD - AZWH] B7019.F10
28. B. concolor [QLD - Gatton] B8484.AU483
29. B. concolor [QLD - Nutgrove via Dalby] B8145
30. B. concolor [QLD - Minden] B3095
31. B. concolor [PNG] B0895
32. B. spratti n. sp. [NSW - Albury, Wodonga] B8632.AU473
33. B. spratti n. sp. [VIC - Phillip Is] B8141
34. B. spratti n. sp. [VIC - Woodend] B5461
35. B. spratti n. sp. [SA - Kangaroo Is] B2296, NC 017756
36. B. spratti n. sp. [SA - Hindmarsh Valley] B6897.AU439
37. B. spratti n. sp. [SA - Kangaroo Is] B5478
38. B. spratti n. sp. [SA - Kangaroo Is] B5481
39. B. spratti n. sp. [SA - Kangaroo Is] B5485

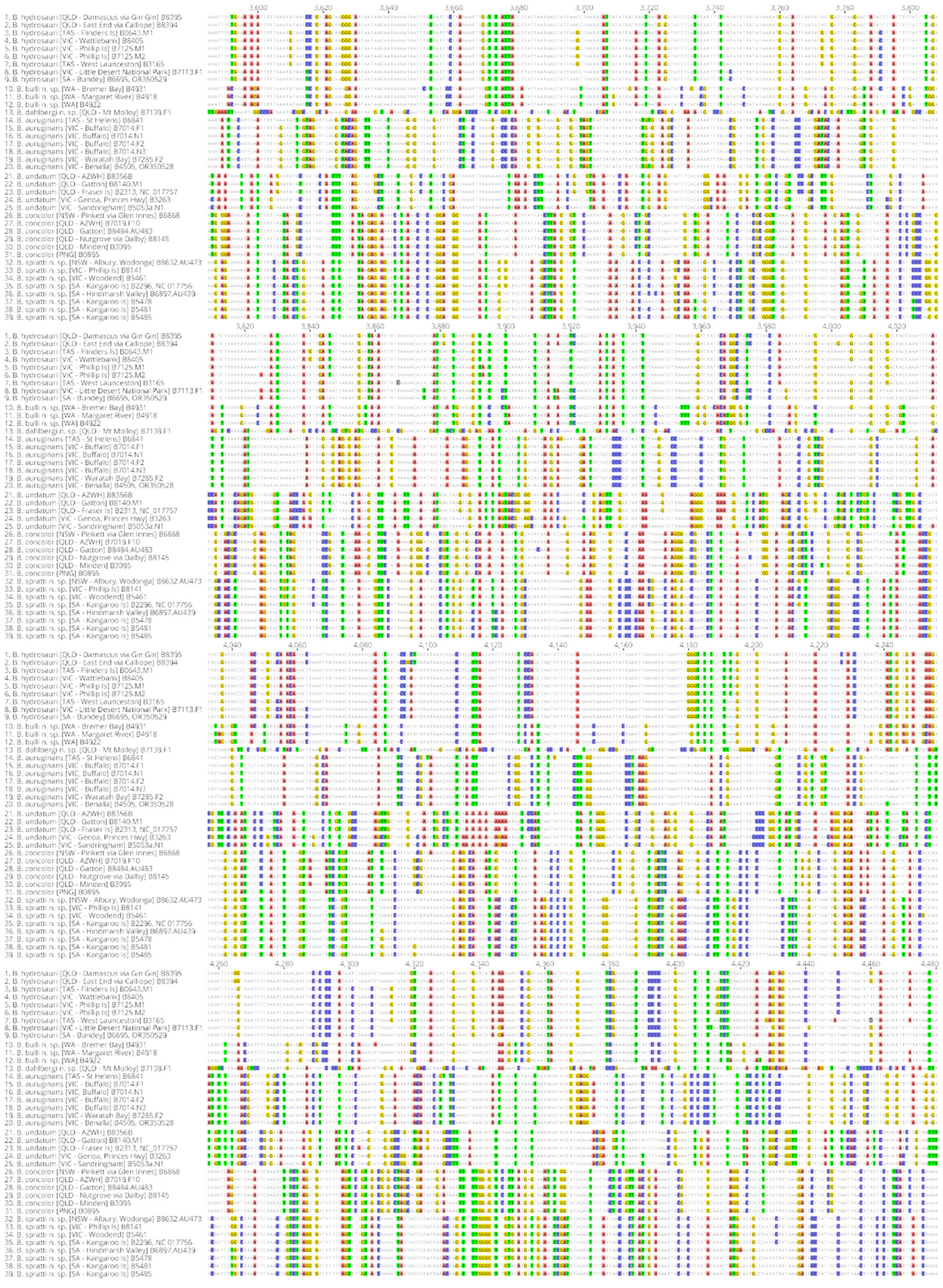
3,600 3,620 3,640 3,660 3,680 3,700 3,720 3,740 3,760 3,780 3,800
3,820 3,840 3,860 3,880 3,900 3,920 3,940 3,960 3,980 4,000 4,020
4,040 4,060 4,080 4,100 4,120 4,140 4,160 4,180 4,200 4,220 4,240
4,260 4,280 4,300 4,320 4,340 4,360 4,380 4,400 4,420 4,440 4,460 4,480
1. B. hydrosauri [QLD - Damascus via Gin Gin] B8395
2. B. hydrosauri [QLD - East End via Calliope] B8394
3. B. hydrosauri [TAS - Flinders Is] B0643.M1
4. B. hydrosauri [VIC - Wattlebank] B8405
5. B. hydrosauri [VIC - Phillip Is] B7125.M1
6. B. hydrosauri [VIC - Phillip Is] B7125.M2
7. B. hydrosauri [TAS - West Launceston] B3165
8. B. hydrosauri [VIC - Little Desert National Park] B7113.F1
9. B. hydrosauri [SA - Bundey] B6695, OR350529
10. B. bulli n. sp. [WA - Bremer Bay] B4931
11. B. bulli n. sp. [WA - Margaret River] B4918
12. B. bulli n. sp. [WA] B4922
13. B. dahlbergi n. sp. [QLD - Mt Molloy] B7139.F1
14. B. auruginans [TAS - St Helens] B6841
15. B. auruginans [VIC - Buffalo] B7014.F1
16. B. auruginans [VIC, Buffalo] B7014.N1
17. B. auruginans [VIC - Buffalo] B7014.F2
18. B. auruginans [VIC - Buffalo] B7014.N3
19. B. auruginans [VIC - Waratah Bay] B7285.F2
20. B. auruginans [VIC - Benalla] B4505, OR350528
21. B. undatum [QLD - AZWH] B8356B
22. B. undatum [QLD - Gatton] B8140.M1
23. B. undatum [QLD - Fraser Is] B2313, NC_017757
24. B. undatum [VIC - Genoa, Princes Hwy] B3263
25. B. undatum [VIC - Sandringham] B5053a.N1
26. B. concolor [NSW - Pinkett via Glen Innes] B6868
27. B. concolor [QLD - AZWH] B7019.F10
28. B. concolor [QLD - Gatton] B8484.AU483
29. B. concolor [QLD - Nutgrove via Dalby] B8145
30. B. concolor [QLD - Minden] B3095
31. B. concolor [PNG] B0895
32. B. spratti n. sp. [NSW - Albury, Wodonga] B8632.AU473
33. B. spratti n. sp. [VIC - Phillip Is] B8141
34. B. spratti n. sp. [VIC - Woodend] B5461
35. B. spratti n. sp. [SA - Kangaroo Is] B2296, NC 017756
36. B. spratti n. sp. [SA - Hindmarsh Valley] B6897.AU439
37. B. spratti n. sp. [SA - Kangaroo Is] B5478
38. B. spratti n. sp. [SA - Kangaroo Is] B5481
39. B. spratti n. sp. [SA - Kangaroo Is] B5485

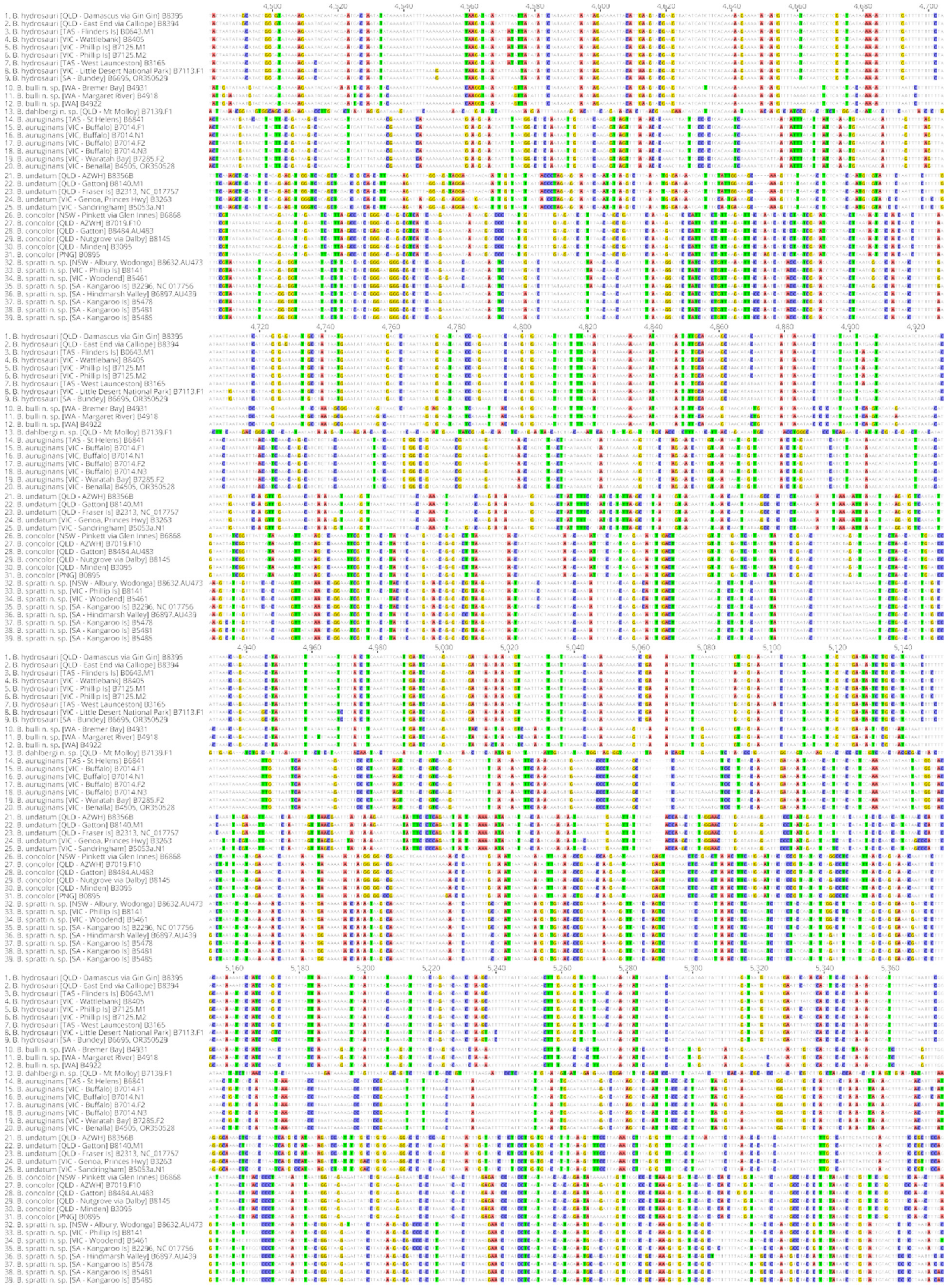

1. B. hydrosauri [QLD - Damascus via Gin Gin] B8395
2. B. hydrosauri [QLD - East End via Calliope] B8394
3. B. hydrosauri [TAS - Flinders Is] B0643.M1
4. B. hydrosauri [VIC - Wattlebank] B8405
5. B. hydrosauri [VIC - Phillip Is] B7125.M1
6. B. hydrosauri [VIC - Phillip Is] B7125.M2
7. B. hydrosauri [TAS - West Launceston] B3165
8. B. hydrosauri [VIC - Little Desert National Park] B7113.F1
9. B. hydrosauri [SA - Bundey] B6695, OR350529
10. B. bulli n. sp. [WA - Bremer Bay] B4931
11. B. bulli n. sp. [WA - Margaret River] B4918
12. B. bulli n. sp. [WA] B4922
13. B. dahlbergi n. sp. [QLD - Mt Molloy] B7139.F1
14. B. auruginans [TAS - St Helens] B6841
15. B. auruginans [VIC - Buffalo] B7014.F1
16. B. auruginans [VIC, Buffalo] B7014.N1
17. B. auruginans [VIC - Buffalo] B7014.F2
18. B. auruginans [VIC - Buffalo] B7014.N3
19. B. auruginans [VIC - Waratah Bay] B7285.F2
20. B. auruginans [VIC - Benalla] B4505, OR350528
21. B. undatum [QLD - AZWH] B8356B
22. B. undatum [QLD - Gatton] B8140.M1
23. B. undatum [QLD - Fraser Is] B2313, NC_017757
24. B. undatum [VIC - Genoa, Princes Hwy] B3263
25. B. undatum [VIC - Sandringham] B5053a.N1
26. B. concolor [NSW - Pinkett via Glen Innes] B6868
27. B. concolor [QLD - AZWH] B7019.F10
28. B. concolor [QLD - Gatton] B8484.AU483
29. B. concolor [QLD - Nutgrove via Dalby] B8145
30. B. concolor [QLD - Minden] B3095
31. B. concolor [PNG] B0895
32. B. spratti n. sp. [NSW - Albury, Wodonga] B8632.AU473
33. B. spratti n. sp. [VIC - Phillip Is] B8141
34. B. spratti n. sp. [VIC - Woodend] B5461
35. B. spratti n. sp. [SA - Kangaroo Is] B2296, NC 017756
36. B. spratti n. sp. [SA - Hindmarsh Valley] B6897.AU439
37. B. spratti n. sp. [SA - Kangaroo Is] B5478
38. B. spratti n. sp. [SA - Kangaroo Is] B5481
39. B. spratti n. sp. [SA - Kangaroo Is] B5485
4,500 4,520 4,540 4,560 4,580 4,600 4,620 4,640 4,660 4,680 4,700
4,720 4,740 4,760 4,780 4,800 4,820 4,840 4,860 4,880 4,900 4,920
4,940 4,960 4,980 5,000 5,020 5,040 5,060 5,080 5,100 5,120 5,140
5,160 5,180 5,200 5,220 5,240 5,260 5,280 5,300 5,320 5,340 5,360

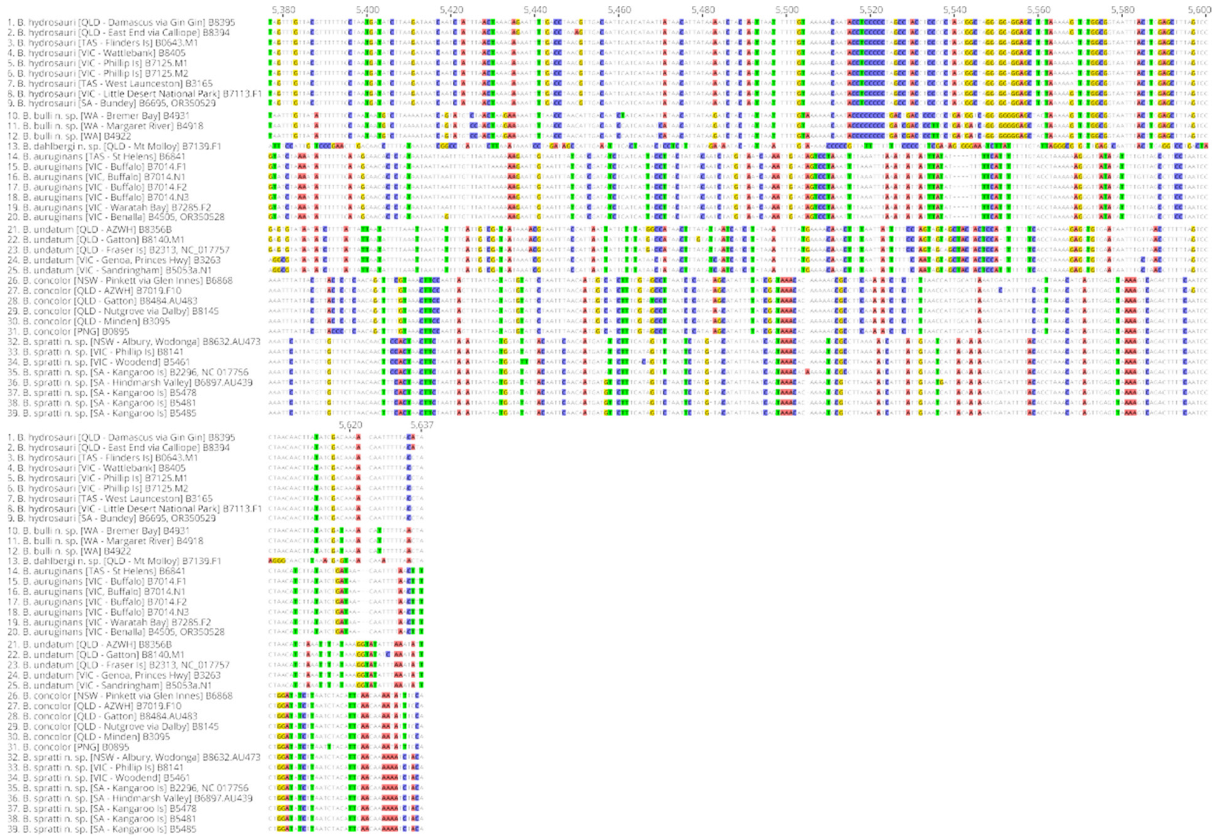

Figure S20. Alignment of 5,637 variable nucleotide sites among the 39 *Bothriocroton* mitochondrial genomes. Invariable sites are not shown. Nucleotides differing from the most common base at each position are highlighted in colour.

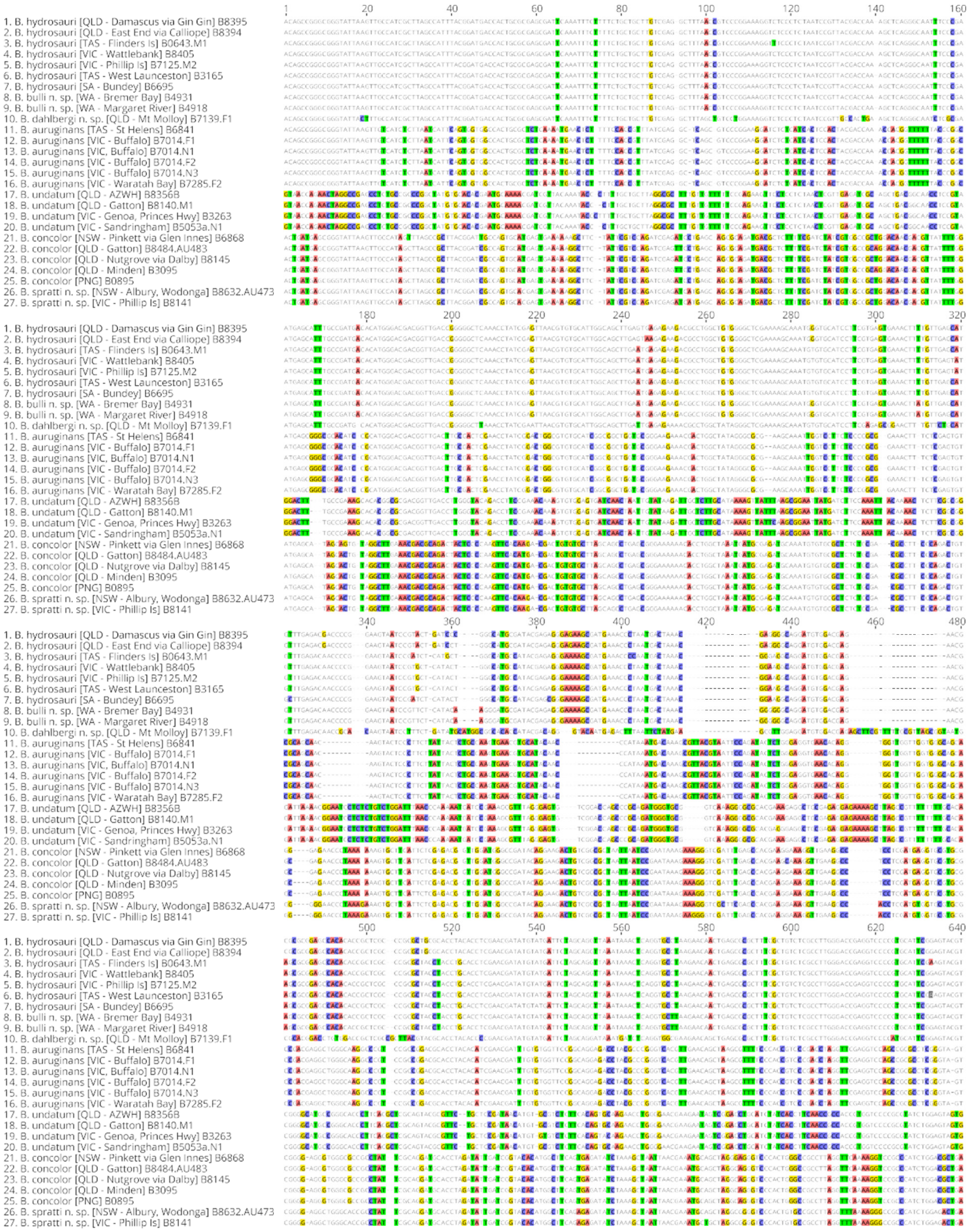
1 20 40 60 80 100 120 140 160
1. B. hydrosauri [QLD - Damascus via Gin Gin] B8395
2. B. hydrosauri [QLD - East End via Calliope] B8394
3. B. hydrosauri [TAS - Flinders Is] B0643.M1
4. B. hydrosauri [VIC - Wattlebank] B8405
5. B. hydrosauri [VIC - Phillip Is] B7125.M2
6. B. hydrosauri [TAS - West Launceston] B3165
7. B. hydrosauri [SA - Bundey] B6695
8. B. bulli n. sp. [WA - Bremer Bay] B4931
9. B. bulli n. sp. [WA - Margaret River] B4918
10. B. dahlbergi n. sp. [QLD - Mt Molloy] B7139.F1
11. B. auruginans [TAS - St Helens] B6841
12. B. auruginans [VIC - Buffalo] B7014.F1
13. B. auruginans [VIC, Buffalo] B7014.N1
14. B. auruginans [VIC - Buffalo] B7014.F2
15. B. auruginans [VIC - Buffalo] B7014.N3
16. B. auruginans [VIC - Waratah Bay] B7285.F2
17. B. undatum [QLD - AZWH] B8356B
18. B. undatum [QLD - Gatton] B8140.M1
19. B. undatum [VIC - Genoa, Princes Hwy] B3263
20. B. undatum [VIC - Sandringham] B5053a.N1
21. B. concolor [NSW - Pinkett via Glen Innes] B6868
22. B. concolor [QLD - Gatton] B8484.AU483
23. B. concolor [QLD - Nutgrove via Dalby] B8145
24. B. concolor [QLD - Minden] B3095
25. B. concolor [PNG] B0895
26. B. spratti n. sp. [NSW - Albury, Wodonga] B8632.AU473
27. B. spratti n. sp. [VIC - Phillip Is] B8141
180 200 220 240 260 280 300 320
1. B. hydrosauri [QLD - Damascus via Gin Gin] B8395
2. B. hydrosauri [QLD - East End via Calliope] B8394
3. B. hydrosauri [TAS - Flinders Is] B0643.M1
4. B. hydrosauri [VIC - Wattlebank] B8405
5. B. hydrosauri [VIC - Phillip Is] B7125.M2
6. B. hydrosauri [TAS - West Launceston] B3165
7. B. hydrosauri [SA - Bundey] B6695
8. B. bulli n. sp. [WA - Bremer Bay] B4931
9. B. bulli n. sp. [WA - Margaret River] B4918
10. B. dahlbergi n. sp. [QLD - Mt Molloy] B7139.F1
11. B. auruginans [TAS - St Helens] B6841
12. B. auruginans [VIC - Buffalo] B7014.F1
13. B. auruginans [VIC, Buffalo] B7014.N1
14. B. auruginans [VIC - Buffalo] B7014.F2
15. B. auruginans [VIC - Buffalo] B7014.N3
16. B. auruginans [VIC - Waratah Bay] B7285.F2
17. B. undatum [QLD - AZWH] B8356B
18. B. undatum [QLD - Gatton] B8140.M1
19. B. undatum [VIC - Genoa, Princes Hwy] B3263
20. B. undatum [VIC - Sandringham] B5053a.N1
21. B. concolor [NSW - Pinkett via Glen Innes] B6868
22. B. concolor [QLD - Gatton] B8484.AU483
23. B. concolor [QLD - Nutgrove via Dalby] B8145
24. B. concolor [QLD - Minden] B3095
25. B. concolor [PNG] B0895
26. B. spratti n. sp. [NSW - Albury, Wodonga] B8632.AU473
27. B. spratti n. sp. [VIC - Phillip Is] B8141
340 360 380 400 420 440 460 480
1. B. hydrosauri [QLD - Damascus via Gin Gin] B8395
2. B. hydrosauri [QLD - East End via Calliope] B8394
3. B. hydrosauri [TAS - Flinders Is] B0643.M1
4. B. hydrosauri [VIC - Wattlebank] B8405
5. B. hydrosauri [VIC - Phillip Is] B7125.M2
6. B. hydrosauri [TAS - West Launceston] B3165
7. B. hydrosauri [SA - Bundey] B6695
8. B. bulli n. sp. [WA - Bremer Bay] B4931
9. B. bulli n. sp. [WA - Margaret River] B4918
10. B. dahlbergi n. sp. [QLD - Mt Molloy] B7139.F1
11. B. auruginans [TAS - St Helens] B6841
12. B. auruginans [VIC - Buffalo] B7014.F1
13. B. auruginans [VIC, Buffalo] B7014.N1
14. B. auruginans [VIC - Buffalo] B7014.F2
15. B. auruginans [VIC - Buffalo] B7014.N3
16. B. auruginans [VIC - Waratah Bay] B7285.F2
17. B. undatum [QLD - AZWH] B8356B
18. B. undatum [QLD - Gatton] B8140.M1
19. B. undatum [VIC - Genoa, Princes Hwy] B3263
20. B. undatum [VIC - Sandringham] B5053a.N1
21. B. concolor [NSW - Pinkett via Glen Innes] B6868
22. B. concolor [QLD - Gatton] B8484.AU483
23. B. concolor [QLD - Nutgrove via Dalby] B8145
24. B. concolor [QLD - Minden] B3095
25. B. concolor [PNG] B0895
26. B. spratti n. sp. [NSW - Albury, Wodonga] B8632.AU473
27. B. spratti n. sp. [VIC - Phillip Is] B8141
500 520 540 560 580 600 620 640
1. B. hydrosauri [QLD - Damascus via Gin Gin] B8395
2. B. hydrosauri [QLD - East End via Calliope] B8394
3. B. hydrosauri [TAS - Flinders Is] B0643.M1
4. B. hydrosauri [VIC - Wattlebank] B8405
5. B. hydrosauri [VIC - Phillip Is] B7125.M2
6. B. hydrosauri [TAS - West Launceston] B3165
7. B. hydrosauri [SA - Bundey] B6695
8. B. bulli n. sp. [WA - Bremer Bay] B4931
9. B. bulli n. sp. [WA - Margaret River] B4918
10. B. dahlbergi n. sp. [QLD - Mt Molloy] B7139.F1
11. B. auruginans [TAS - St Helens] B6841
12. B. auruginans [VIC - Buffalo] B7014.F1
13. B. auruginans [VIC, Buffalo] B7014.N1
14. B. auruginans [VIC - Buffalo] B7014.F2
15. B. auruginans [VIC - Buffalo] B7014.N3
16. B. auruginans [VIC - Waratah Bay] B7285.F2
17. B. undatum [QLD - AZWH] B8356B
18. B. undatum [QLD - Gatton] B8140.M1
19. B. undatum [VIC - Genoa, Princes Hwy] B3263
20. B. undatum [VIC - Sandringham] B5053a.N1
21. B. concolor [NSW - Pinkett via Glen Innes] B6868
22. B. concolor [QLD - Gatton] B8484.AU483
23. B. concolor [QLD - Nutgrove via Dalby] B8145
24. B. concolor [QLD - Minden] B3095
25. B. concolor [PNG] B0895
26. B. spratti n. sp. [NSW - Albury, Wodonga] B8632.AU473
27. B. spratti n. sp. [VIC - Phillip Is] B8141

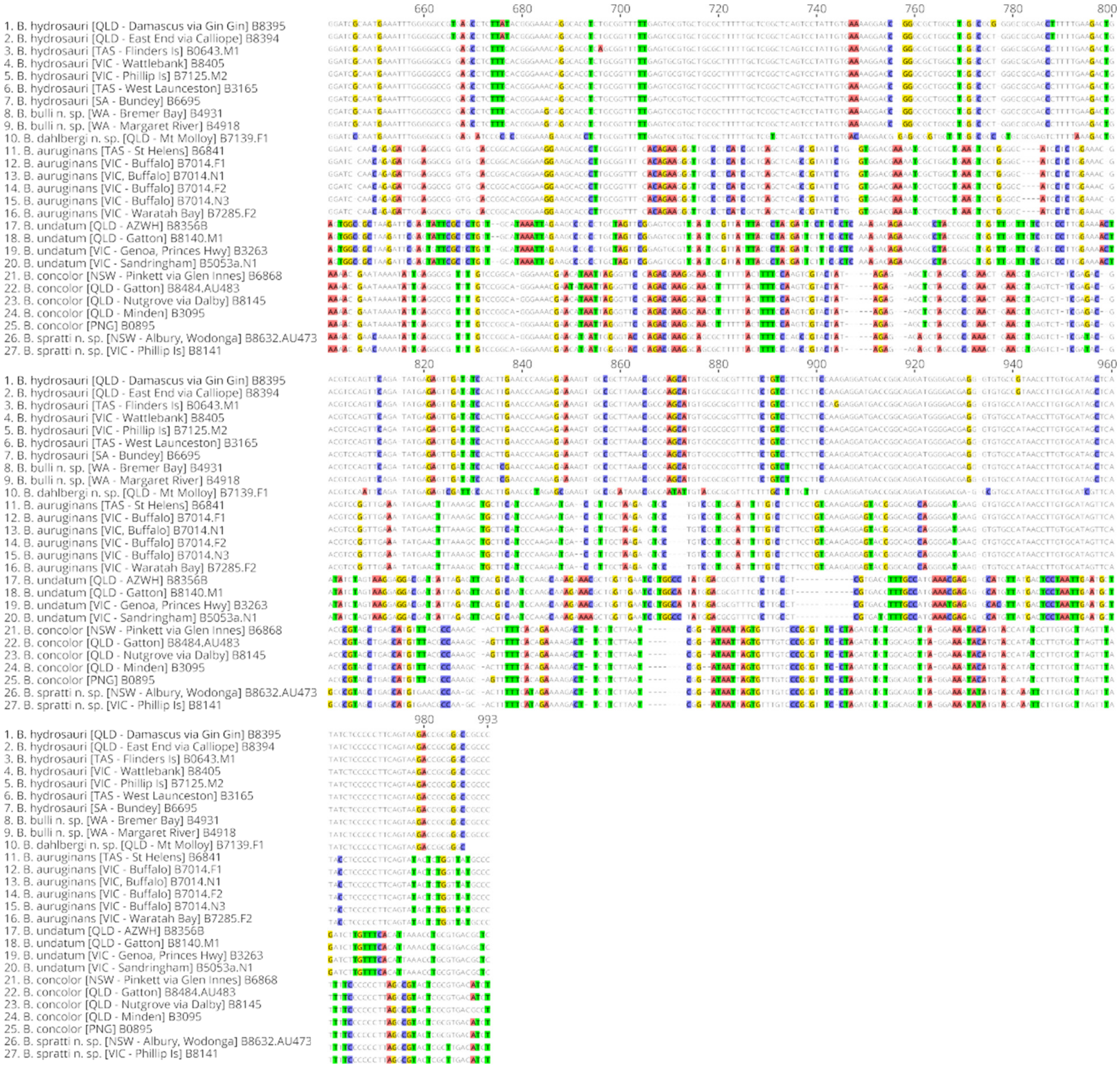

Figure S21. Alignment of 993 variable nucleotide sites among the 27 *Bothriocroton* rDNA sequences (18S rRNA, ITS1, ITS2 and 28S rRNA. Invariable sites are not shown. Nucleotides differing from the most common base at each position are highlighted in colour.

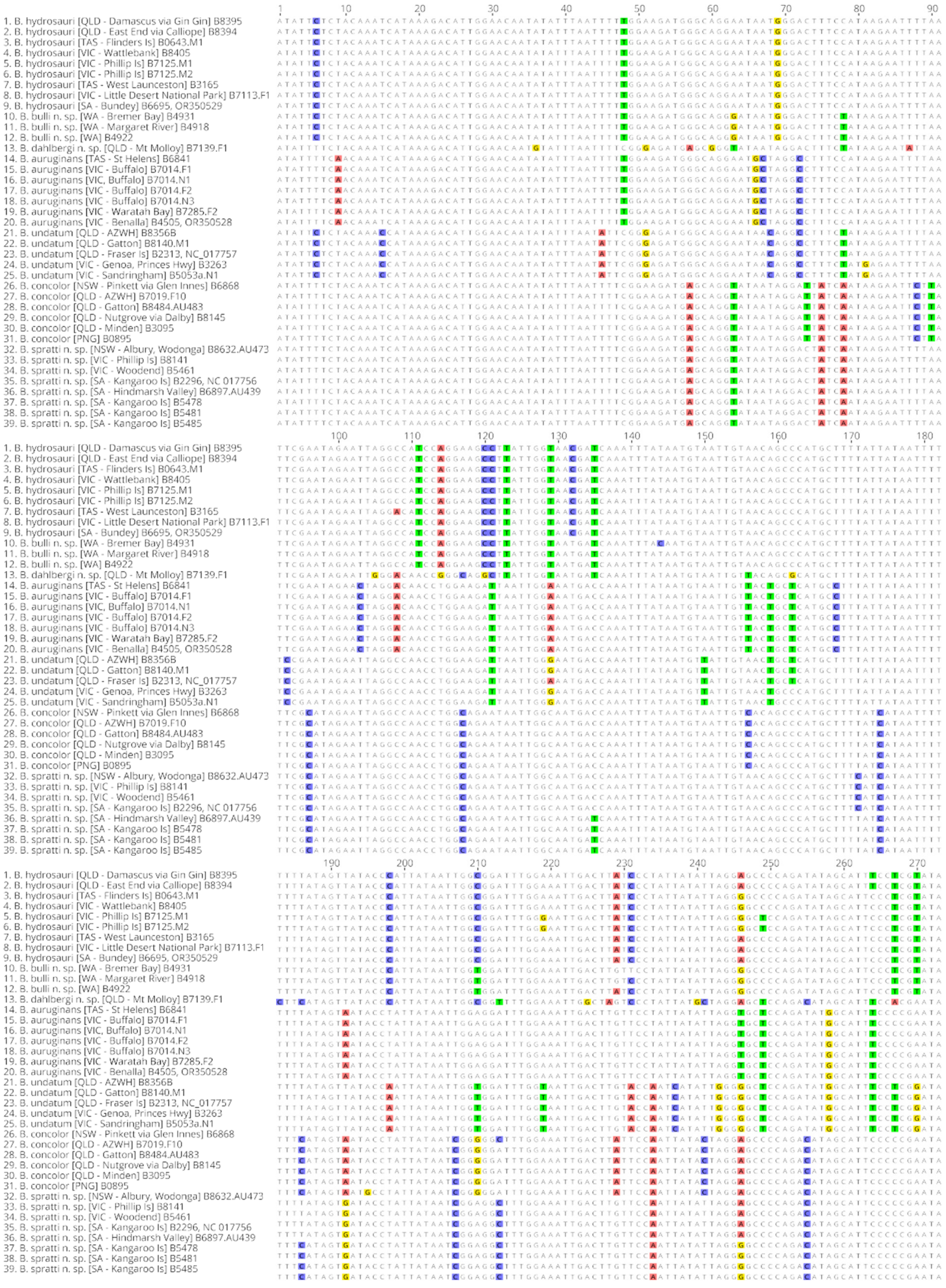
1. B. hydrosauri [QLD - Damascus via Gin Gin] B8395
2. B. hydrosauri [QLD - East End via Calliope] B8394
3. B. hydrosauri [TAS - Flinders Is] B0643.M1
4. B. hydrosauri [VIC - Wattlebank] B8405
5. B. hydrosauri [VIC - Phillip Is] B7125.M1
6. B. hydrosauri [VIC - Phillip Is] B7125.M2
7. B. hydrosauri [TAS - West Launceston] B3165
8. B. hydrosauri [VIC - Little Desert National Park] B7113.F1
9. B. hydrosauri [SA - Bundey] B6695, OR350529
10. B. bulli n. sp. [WA - Bremer Bay] B4931
11. B. bulli n. sp. [WA - Margaret River] B4918
12. B. bulli n. sp. [WA] B4922
13. B. dahlbergi n. sp. [QLD - Mt Molloy] B7139.F1
14. B. auruginans [TAS - St Helens] B6841
15. B. auruginans [VIC - Buffalo] B7014.F1
16. B. auruginans [VIC, Buffalo] B7014.N1
17. B. auruginans [VIC - Buffalo] B7014.F2
18. B. auruginans [VIC - Buffalo] B7014.N3
19. B. auruginans [VIC - Waratah Bay] B7285.F2
20. B. auruginans [VIC - Benalla] B4505, OR350528
21. B. undatum [QLD - AZWH] B8356B
22. B. undatum [QLD - Gatton] B8140.M1
23. B. undatum [QLD - Fraser Is] B2313, NC_017757
24. B. undatum [VIC - Genoa, Princes Hwy] B3263
25. B. undatum [VIC - Sandringham] B5053a.N1
26. B. concolor [NSW - Pinkett via Glen Innes] B6868
27. B. concolor [QLD - AZWH] B7019.F10
28. B. concolor [QLD - Gatton] B8484.AU483
29. B. concolor [QLD - Nutgrove via Dalby] B8145
30. B. concolor [QLD - Minden] B3095
31. B. concolor [PNG] B0895
32. B. spratti n. sp. [NSW - Albury, Wodonga] B8632.AU473
33. B. spratti n. sp. [VIC - Phillip Is] B8141
34. B. spratti n. sp. [VIC - Woodend] B5461
35. B. spratti n. sp. [SA - Kangaroo Is] B2296, NC 017756
36. B. spratti n. sp. [SA - Hindmarsh Valley] B6897.AU439
37. B. spratti n. sp. [SA - Kangaroo Is] B5478
38. B. spratti n. sp. [SA - Kangaroo Is] B5481
39. B. spratti n. sp. [SA - Kangaroo Is] B5485
1 10 20 30 40 50 60 70 80 90
100 110 120 130 140 150 160 170 180
190 200 210 220 230 240 250 260 270

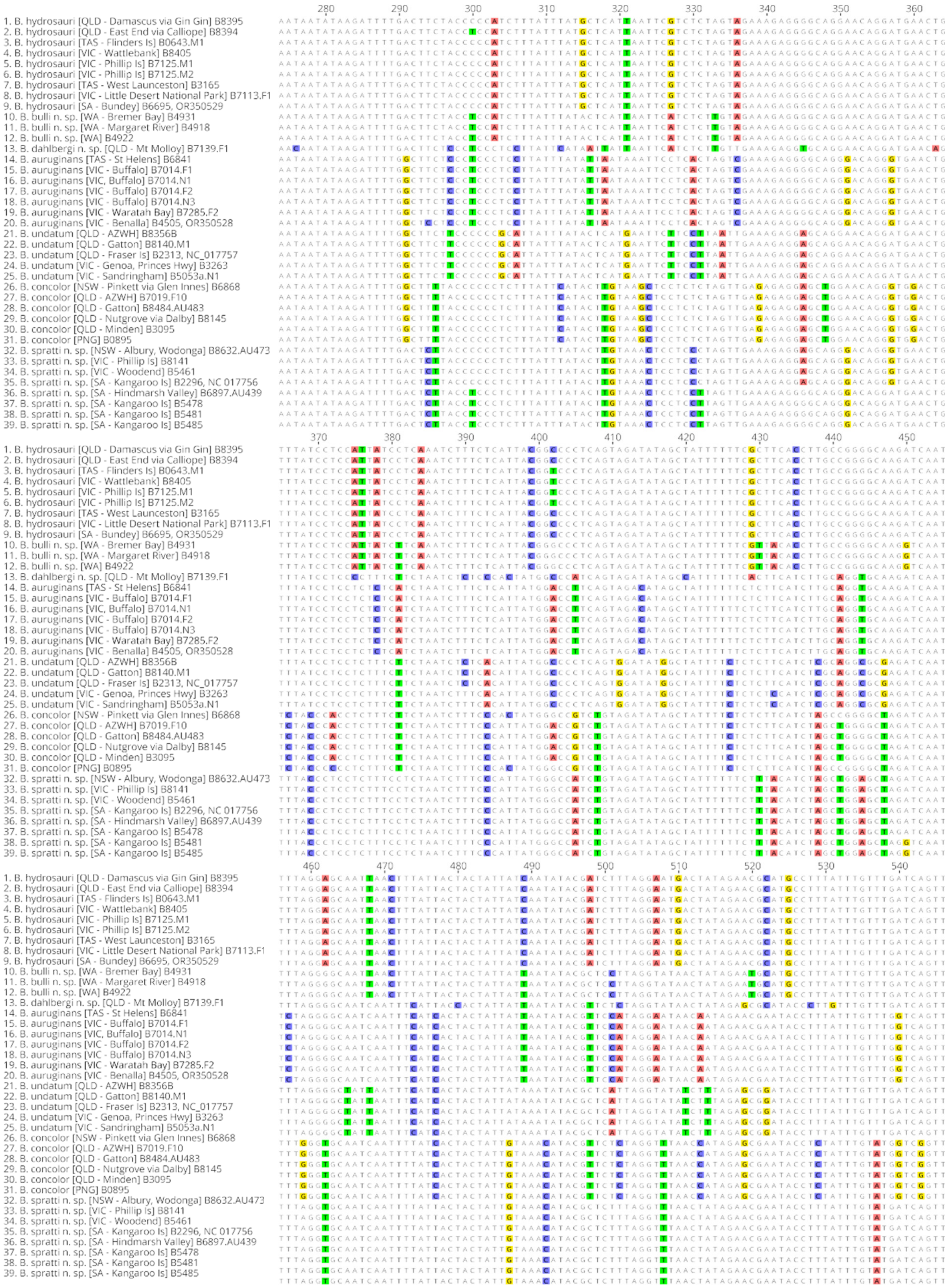
280 290 300 310 320 330 340 350 360
370 380 390 400 410 420 430 440 450
460 470 480 490 500 510 520 530 540
1. B. hydrosauri [QLD - Damascus via Gin Gin] B8395
2. B. hydrosauri [QLD - East End via Calliope] B8394
3. B. hydrosauri [TAS - Flinders Is] B0643.M1
4. B. hydrosauri [VIC - Wattlebank] B8405
5. B. hydrosauri [VIC - Phillip Is] B7125.M1
6. B. hydrosauri [VIC - Phillip Is] B7125.M2
7. B. hydrosauri [TAS - West Launceston] B3165
8. B. hydrosauri [VIC - Little Desert National Park] B7113.F1
9. B. hydrosauri [SA - Bundey] B6695, OR350529
10. B. bulli n. sp. [WA - Bremer Bay] B4931
11. B. bulli n. sp. [WA - Margaret River] B4918
12. B. bulli n. sp. [WA] B4922
13. B. dahlbergi n. sp. [QLD - Mt Molloy] B7139.F1
14. B. auruginans [TAS - St Helens] B6841
15. B. auruginans [VIC - Buffalo] B7014.F1
16. B. auruginans [VIC, Buffalo] B7014.N1
17. B. auruginans [VIC - Buffalo] B7014.F2
18. B. auruginans [VIC - Buffalo] B7014.N3
19. B. auruginans [VIC - Waratah Bay] B7285.F2
20. B. auruginans [VIC - Benalla] B4505, OR350528
21. B. undatum [QLD - AZWH] B8356B
22. B. undatum [QLD - Gatton] B8140.M1
23. B. undatum [QLD - Fraser Is] B2313, NC_017757
24. B. undatum [VIC - Genoa, Princes Hwy] B3263
25. B. undatum [VIC - Sandringham] B5053a.N1
26. B. concolor [NSW - Pinkett via Glen Innes] B6868
27. B. concolor [QLD - AZWH] B7019.F10
28. B. concolor [QLD - Gatton] B8484.AU483
29. B. concolor [QLD - Nutgrove via Dalby] B8145
30. B. concolor [QLD - Minden] B3095
31. B. concolor [PNG] B0895
32. B. spratti n. sp. [NSW - Albury, Wodonga] B8632.AU473
33. B. spratti n. sp. [VIC - Phillip Is] B8141
34. B. spratti n. sp. [VIC - Woodend] B5461
35. B. spratti n. sp. [SA - Kangaroo Is] B2296, NC 017756
36. B. spratti n. sp. [SA - Hindmarsh Valley] B6897.AU439
37. B. spratti n. sp. [SA - Kangaroo Is] B5478
38. B. spratti n. sp. [SA - Kangaroo Is] B5481
39. B. spratti n. sp. [SA - Kangaroo Is] B5485

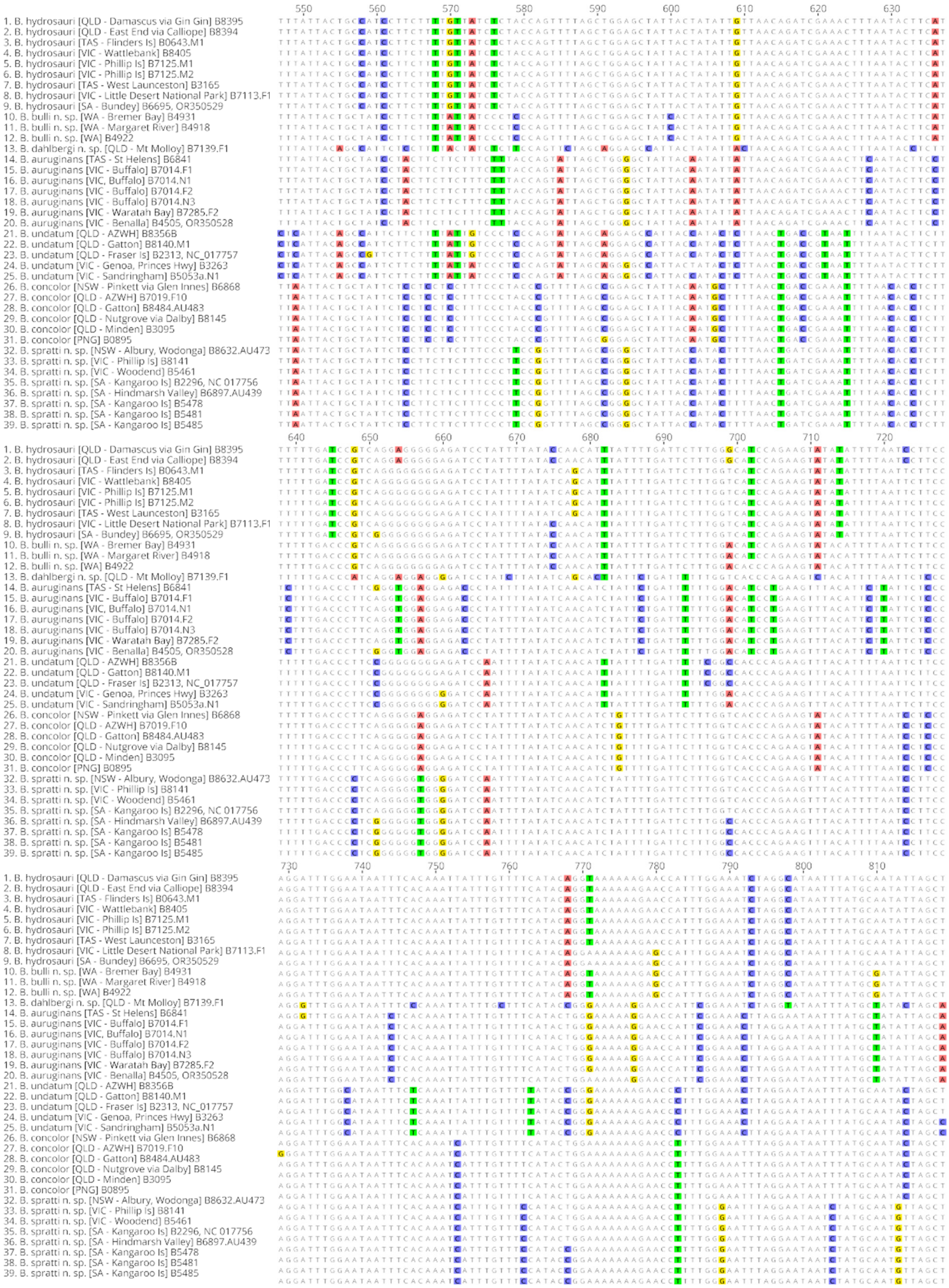
550 560 570 580 590 600 610 620 630
1. B. hydrosauri [QLD - Damascus via Gin Gin] B8395
2. B. hydrosauri [QLD - East End via Calliope] B8394
3. B. hydrosauri [TAS - Flinders Is] B0643.M1
4. B. hydrosauri [VIC - Wattlebank] B8405
5. B. hydrosauri [VIC - Phillip Is] B7125.M1
6. B. hydrosauri [VIC - Phillip Is] B7125.M2
7. B. hydrosauri [TAS - West Launceston] B3165
8. B. hydrosauri [VIC - Little Desert National Park] B7113.F1
9. B. hydrosauri [SA - Bundey] B6695, OR350529
10. B. bulli n. sp. [WA - Bremer Bay] B4931
11. B. bulli n. sp. [WA - Margaret River] B4918
12. B. bulli n. sp. [WA] B4922
13. B. dahlbergi n. sp. [QLD - Mt Molloy] B7139.F1
14. B. auruginans [TAS - St Helens] B6841
15. B. auruginans [VIC - Buffalo] B7014.F1
16. B. auruginans [VIC, Buffalo] B7014.N1
17. B. auruginans [VIC - Buffalo] B7014.F2
18. B. auruginans [VIC - Buffalo] B7014.N3
19. B. auruginans [VIC - Waratah Bay] B7285.F2
20. B. auruginans [VIC - Benalla] B4505, OR350528
21. B. undatum [QLD - AZWH] B8356B
22. B. undatum [QLD - Gatton] B8140.M1
23. B. undatum [QLD - Fraser Is] B2313, NC_017757
24. B. undatum [VIC - Genoa, Princes Hwy] B3263
25. B. undatum [VIC - Sandringham] B5053a.N1
26. B. concolor [NSW - Pinkett via Glen Innes] B6868
27. B. concolor [QLD - AZWH] B7019.F10
28. B. concolor [QLD - Gatton] B8484.AU483
29. B. concolor [QLD - Nutgrove via Dalby] B8145
30. B. concolor [QLD - Minden] B3095
31. B. concolor [PNG] B0895
32. B. spratti n. sp. [NSW - Albury, Wodonga] B8632.AU473
33. B. spratti n. sp. [VIC - Phillip Is] B8141
34. B. spratti n. sp. [VIC - Woodend] B5461
35. B. spratti n. sp. [SA - Kangaroo Is] B2296, NC 017756
36. B. spratti n. sp. [SA - Hindmarsh Valley] B6897.AU439
37. B. spratti n. sp. [SA - Kangaroo Is] B5478
38. B. spratti n. sp. [SA - Kangaroo Is] B5481
39. B. spratti n. sp. [SA - Kangaroo Is] B5485
640 650 660 670 680 690 700 710 720
730 740 750 760 770 780 790 800 810

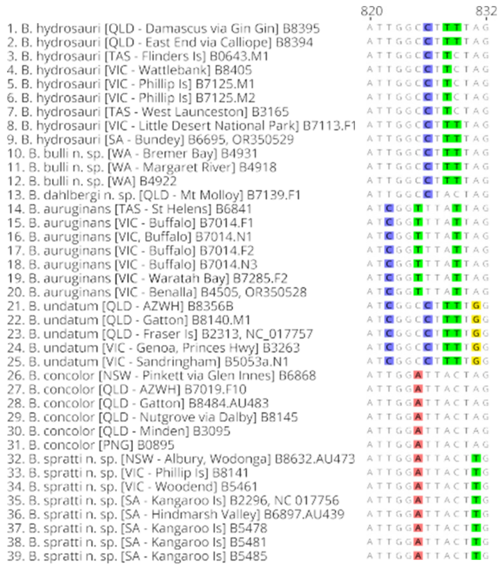

Figure S22. Alignment of 832 nucleotide sites among the 39 *Bothriocroton cox1* sequences amplified by PCR primers adapted from Song et al. (2010). Nucleotides differing from the most common base at each position are highlighted in colour. Sequences were trimmed to uniform length.
